# Supplementary material for: Anterior transversalis fascia approach versus preperitoneal space approach for inguinal hernia repair in residents in northern China: study protocol for a prospective, multicentre, randomised, controlled trial
Source: BMJ Open. 2017 Aug 31;7(8):e016481. doi: 10.1136/bmjopen-2017-016481 (PMC5588954; doi:10.1136/bmjopen-2017-016481)
Supplement: Supplementary file 1 [file bmjopen-2017-016481supp001.pdf]

Additional file 1: Trial committee organization and contributions and role.

Principal investigator (PI) and research physicians at main study site

Organization: the Fourth Affiliated Hospital of China Medical University

Contributions and role: Principal investigator, original study design: Hang-yu Li (Email:li_hangyu@126.com)

Organization of Steering committee and member appointment: Qing Fan, De-wei Zhang, Da-ye Yang, Hong-wu Li, Shi-bo Wei, Liang Yang, Hang-yu Li, Fu-quan Yang, Shao-jun Zhang, Yao-qiang Wu, Wei-de An, Zhong-shu Dai, Hui-yong Jiang, Fu-rong Wang, Shi-feng Qiao

Communication and exchange of opinion with PI at each site

Preparation of IRB documents and CRF

Trial management (randomized allocation management, AE data collection at each site, participant enrollment supervision, study site inspection and visits, budget allocation and management)

Cooperation with CRO in data collection, quality control, monitoring, and analysis

Steering committee (SC)

Organization and role: All authors of this manuscript

Contributions: Protocol revision and decision on final protocol

Organization of Trial Management Committee and member appointment

Designation of participant recruitment study sites

Inspection of study progress, and decision on protocol revision, if needed

Determination of study result publication timing and method

Decision on authorship in accordance with Authorship eligibility guidelines

Trial Management Committee

Organization: PI and investigators at each clinical trial participant enrollment site

Fu-quan Yang, Shengjing Hospital of China Medical University

Shao-jun Zhang, Fengtian Hospital of Shenyang Medical College

Yao-qiang Wu, The First Hospital of Dandong City

Wei-de An, The First Affiliated Hospital of Dalian Medical University

Zhong-shu Dai, General Hospital of Benxi Steel and Iron (Group) Co., LTD, Fifth Clinical College of China Medical University

Hui-yong Jiang, General Hospital of Shenyang Military Area

Fu-rong Wang, The 202nd Hospital of PLA

Shi-feng Qiao, The First Affiliated Hospital of Liaoning Medical University

Hang-yu Li*, The Fourth Affiliated Hospital of China Medical University

Organization and role: Submission and obtaining study protocol approval from relevant IRB of each study site

Clinical trial execution following protocol (e.g. participant recruitment, enrollment, data collection, CRF

entry)

Collection and report of AEs
