# Supplementary material for: Anterior transversalis fascia approach versus preperitoneal space approach for inguinal hernia repair in residents in northern China: study protocol for a prospective, multicentre, randomised, controlled trial
Source: BMJ Open. 2017 Aug 31;7(8):e016481. doi: 10.1136/bmjopen-2017-016481 (PMC5588954; doi:10.1136/bmjopen-2017-016481)
Supplement: Supplementary file 3 [file bmjopen-2017-016481supp003.pdf]

Additional file 3: List of ethical approval documents

Center	Name of ethical bodies	Ethical approval reference number
Department of General Surgery, the Fourth Affiliated Hospital of China Medical University	The Ethics Committee of the Fourth Affiliated Hospital of China Medical University	2015-027
Department of General Surgery, Branch 3, First Hospital of Dalian Medical University	The Ethics Committee of First Hospital of Dalian Medical University	2016-034
Department of General Surgery, the 202 Hospital of Chinese PLA	The Ethics Committee of the 202 Hospital of Chinese PLA	2015-033
Ward of Hernia, Department of General and Gastrointestinal Surgery, First Affiliated Hospital of Liaoning Medical University	The Ethics Committee of First Affiliated Hospital of Liaoning Medical University	2016-022
Department of General Surgery, General Hospital of Shenyang Military	The Ethics Committee of General Hospital of Shenyang Military	2015-015
Second Department of General Surgery, General Hospital of Benxin Iron and Steel Co., Ltd.	The Ethics Committee of General Hospital of Benxin Iron and Steel Co., Ltd.	2015-011
First Department of General	The Ethics Committee of Affiliated	2015-022

Surgery, Affiliated Central Hospital of Shenyang Medical University	Central Hospital of Shenyang Medical University	
Department of General Surgery, First Hospital of Dandong	The Ethics Committee of First Hospital of Dandong	2016-016
Shengjing Hospital of China Medical University	The Ethics Committee of Shengjing Hospital of China Medical University	2016-010
