# Supplementary material for: Anterior transversalis fascia approach versus preperitoneal space approach for inguinal hernia repair in residents in northern China: study protocol for a prospective, multicentre, randomised, controlled trial
Source: BMJ Open. 2017 Aug 31;7(8):e016481. doi: 10.1136/bmjopen-2017-016481 (PMC5588954; doi:10.1136/bmjopen-2017-016481)
Supplement: Supplementary file 4 [file bmjopen-2017-016481supp004.pdf]

Informed Consent Form for Clinical Studies

[Name of Principal Investigator] Hang-yu Li
[Name of Organization] The Fourth Affiliated Hospital of China Medical University, China
[Name of Proposal and version] Anterior transversalis fascia approach versus preperitoneal space approach for inguinal hernia repair in residents in Northern China: study protocol for a prospective, multi-center, randomized, controlled trial (Version 1.0)

This Informed Consent Form has two parts:

- **Information Sheet (to share information about the research with you)**
- **Certificate of Consent (for signatures if you agree to take part)**

You will be given a copy of the full Informed Consent Form

PART I: Information Sheet

Introduction

I am Dr. Hang-yu Li, working for the Fourth Affiliated Hospital of China Medical University. We are doing research on different surgical techniques used to repair abnormal wall defects. I am going to give you information and invite you to be part of this research. You do not have to decide today whether or not you will participate in the research. Before you decide, you can talk to anyone you feel comfortable with about the research.

There may be some words that you do not understand. Please ask me to stop as we go through the information and I will take time to explain. If you have questions later, you can ask them of me, the study doctor or the staff.

Purpose of the research

There are few reports on the effects of different inguinal hernia repair approaches on postoperative complications, particularly regarding severe postoperative complications, in patients from northern China. The incidence of severe complications after inguinal hernia repair is relatively low, but this surgery can lead to physical impairment or organ dysfunction that greatly decreases patient quality of life and places a heavy burden on the patient’s family and society. This study will compare the effectiveness of inguinal hernia repair via the anterior transversalis fascia approach versus the preperitoneal approach in patients from northern China in terms of postoperative complications, quality of life, and cost-effectiveness. This study aims to determine the optimal method for inguinal hernia repair that is suitable for the anatomic features of the inguinal region for local patients.

Type of Research Intervention

Briefly state the type of intervention that will be undertaken. This will be expanded upon in the procedures section but it may be helpful and less confusing to the participant if they

know from the very beginning whether, for example, the research involves a vaccine, an interview, a biopsy or a series of finger pricks.
You will be undergone inguinal hernia repair via the anterior transversalis fascia approach or the preperitoneal approach. Also you will be followed-up at the following time-points: at baseline (at admission), pre-discharge, and at 1 week, 1 month, 3 months (hospital visit or telephone follow-up), 1 year (telephone follow-up), and 2 years after surgery (telephone follow-up).

Participant selection
We are inviting all male adults with inguinal hernia who attend our hospital to participate in the research.

- ***Example of question to elucidate understanding:*** *Do you know why we are asking you to take part in this study? Do you know what the study is about?*

Voluntary Participation
Your participation in this research is entirely voluntary. It is your choice whether to participate or not. Whether you choose to participate or not, all the services you receive at this hospital will continue and nothing will change. If you choose not to participate in this research project, you will offered the treatment that is routinely offered in this hospital for inguinal hernia repair, and we will tell you more about it later. You may change your mind later and stop participating even if you agreed earlier.

- ***Examples of question to elucidate understanding:*** *If you decide not to take part in this research study, do you know what your options are? Do you know that you do not have to take part in this research study, if you do not wish to? Do you have any questions?*

Include the following section only if the protocol is for a clinical trial:

Information on the different approaches for inguinal hernia repair
1) What is the anterior transversalis fascia repair?
An oblique skin incision will be made parallel to the inguinal groove from the inner to the outer inguinal ring. Subcutaneous tissue will be dissected until the external oblique aponeurosis is reached. Hemostasis will be achieved by electric coagulation. A4－6 cm long skin incision will be made on the external oblique aponeurosis, starting from the pubic tubercle, to fully expose the pubic tubercle and the inner ring.
The lower part of the external oblique aponeurosis will be separated from the spermatic cord. The upper part of the external oblique aponeurosis will then be separated from the internal oblique aponeurosis and dissociated to a point 3 cm above the inguinal canal wall. The overlying spermatic cord and cremaster muscle will be lifted up and disassociated about 2 cm above the pubic tubercle. The ilioinguinal nerve, external spermatic vessels, and genital nerves concomitant with the spermatic cord will be protected while the spermatic cord is lifted.
The inguinal canal will then be dissected and the herniasac will be dissociated from the surrounding tissue. The cremaster muscle fibers will be separated longitudinally and the hernia sac will be separated from the spermatic cord. The hernia sac will be dissociated until the neck of the hernia sac is reached. All abdominal contents in the hernia sac will be returned to the abdominal cavity. In cases with a small hernia sac, the sac will also be returned to the anterior peritoneal cavity to minimize postoperative pain. In cases with a large hernia sac, the distal end will be transected and opened, and the proximal part will be ligated at a high position.
A patch designed with an arc-shaped head and a swallow tail-like end that comprises two pieces (a wide top piece and a narrow bottom piece) will be sutured and fixed with non-absorbable

polypropylene sutures. The inner side of the patch will be sutured to the pubic tubercle and the lateral boundary of the rectus abdominis muscle. The upper border of the patch will be sutured to the conjoined tendon and its border with the inguinal ligament and iliopubic tract. The wound will then be closed using layered sutures according to anatomic position.

2) What is the preperitoneal space repair?

The skin will be cut and subcutaneous tissue will be dissected. The abdominal external oblique aponeurosis will be dissected along the fibers. The abdominal internal oblique aponeurosis and transversus abdominis muscle will be bluntly separated to expose the transverse fascia. Dissociation of the hernia sac will be done as described above in the anterior transversalis fascia repair.

Transabdominal preperitoneal or totally extraperitoneal laparoscopic techniques were used to dissociate preperitoneal space, strip spermatic cord peritoneum, anatomize and expose the pubic pore. A polypropylene patch will be placed in the prevesical space and Bogros' space to achieve repair of the pubic pore-containing area.

3) Why we compare these two approaches?

Many surgical repair methods involving patches (of varying types and materials) are available for inguinal hernia repair. At present, there is a scarcity of clinical evidence from large-sample, randomized, controlled trials investigating the effectiveness of inguinal hernia repair via the anterior transversalis fascia approach versus the preperitoneal approach. Our outcomes will provide trial-based evidence for selection of rational therapeutic regimen in the clinic.

4) What are the side-effects of the repairs?

There are few reports on the effects of different inguinal hernia repair approaches on postoperative complications, particularly regarding severe postoperative complications, in patients in northern China. The incidence of severe complications after inguinal hernia repair is relatively low, but this surgery can lead to physical impairment or organ dysfunction that greatly decreases patient quality of life and places a heavy burden on the patient’s family and society.

Procedures and Protocol

You will receive the treatment of inguinal hernia repair according to national guidelines, i.e. Guidelines for the Diagnosis and Treatment of Inguinal Hernia in Adults (2014 Edition) formulated by Chinese national society and the Adult Inguinal Hernia Treatment Guidelines (Updated Edition in 2014) developed by the European Hernia Society. This means that you will be treated either by the anterior transversalis fascia approach or the preperitoneal approach during the surgery.

In strict accordance with the Consolidated Standards of Reporting Trials (CONSORT) standards, the baseline data (preoperative data), therapeutic regimen, therapeutic outcome, and medical costs during hospitalization of patients with inguinal hernia will be recorded. Patient data will be collected using an electronic data capture system (EDC).

Description of the Process

During the research, you make seven visits to the hospital.

Visit 1 (at admission, day 0):: you will be requested to

- Sign informed consent
- Recheck inclusion/exclusion criteria
- Demographic data (sex, age, body height, body mass index) and medical insurance type
- Type of hernia (indirect or direct)
- Inguinal hernia classification as type I, II, III, or IV according to the adult inguinal hernia and femoral hernia classification (revised in 2003) developed by the Hernia and Abdominal Wall Surgery Group, Society of Surgery, Chinese Medical Association [12]

- ASA classification
- History of diseases and risk factors* (diabetes mellitus, cardiovascular disease, lung disease, peripheral vascular disease, dementia, hypertension, smoking history and alcohol use history) (*optional evaluation items)
- Disease onset and admission (interval from first appearance of the lump, main symptoms)
- Imaging examination (ultrasound, CT)
- Laboratory examination (routine blood testing, coagulation testing, testing of blood glucose, lipids and electrolytes, and hepatic and renal function)
- Vital signs (body temperature, pulse, respiration rate, blood pressure)
- VAS pain score
- Electrocardiography
- Concomitant treatment

Visit 2 (Pre-discharge): you will be tested

- Vital signs (body temperature, pulse, respiration rate, blood pressure)
- VAS pain score
- Laboratory examination (routine blood testing, coagulation testing, testing of blood glucose, lipids and electrolytes, and hepatic and renal function)
- Treatment regimen (inguinal hernia-specific treatment)

Surgical approach and patches (type and fixation method); anesthesia method; drug name (generic preferred), dosage, route, start date, length of drug use will be recorded.

- Concomitant treatment: Inguinal hernia-specific treatment, maintenance therapy, and drugs used for concomitant treatment.

Visit 3 (1 week after surgery via clinic visit or telephone follow-up): you will be tested

- VAS pain score
- 36-Item Short Form Health Survey (SF-36) score
- Hernia recurrence
- Presence or absence of hematoma and/or seroma
- Wound infection

Visit 4 (1 month after surgery via clinic visit or telephone follow-up): you will be tested

- VAS pain score
- SF-36 score
- Presence or absence of hematoma and/or seroma
- Wound infection

Visit 5 (3 months after surgery via clinic visit or telephone follow-up): you will be tested

- VAS pain score
- SF-36 score
- Hernia recurrence
- Presence or absence of hematoma and/or seroma
- Wound infection

<u>Visit 6 (1 year after surgery via telephone follow-up): you will be tested</u>

- VAS pain score
- SF-36 score
- Hernia recurrence
- Presence or absence of hematoma and/or seroma
- Wound infection

<u>Visit 7 (2 years after surgery via telephone follow-up): you will be tested</u>

- VAS pain score
- SF-36 score
- Hernia recurrence
- Presence or absence of hematoma and/or seroma
- Wound infection

Duration

In total, you will be asked to come 7 times to the hospital in 2 years. At the end of 2 years, the research will be finished.

- ***<u>Examples of question to elucidate understanding:</u>*** *Can you tell me if you remember the number of times that we are asking you to come to the hospital to complete the treatment? The research project? How many injections will you be given? How many tablets? How much blood will be taken from your veins, using a syringe and needle? Over how many weeks? Etc. Do you have any other questions? Do you want me to go through the procedures again?*

Side Effects

The incidence of severe complications after inguinal hernia repair is relatively low, any adverse medical events occurring after herniorrhaphy in patients or clinical subjects. This surgery could lead to physical impairment or organ dysfunction that decreases patient quality of life.
Serious adverse postoperative medical events involving one or more of the following criteria:

- Death, irrespective of the cause
- Life-threatening event
- Severe or permanent disability or organ dysfunction
- Hemorrhage
- Malformation, birth defect
- Hospitalization or extended hospital stay
- Re-admission to hospital
- Recurrence (symptomatic) of inguinal hernia

Risks

History of diabetes mellitus, cardiovascular disease, lung disease, peripheral vascular disease, dementia, hypertension, smoking history and alcohol use history could cause possible risks, including wound infection, rates of hematoma, seroma, hernia recurrence, intestinal obstruction, intestinal fistula, entocele, mesenteric thrombosis, femoral vein thrombosis, ischemic orchitis, painful ejaculation, varicocele, scrotal edema and etc.

While the possibility of this happening is very low, you should still be aware of the possibility. We will try to decrease the chances of this event occurring, but if something unexpected happens

- ***Examples of question to elucidate understanding:*** *Do you understand that these risks can happen whether or not you are in the research study? Etc. Do you have any other questions?*

Benefits
There may not be any benefit for you but your participation is likely to help us find the answer to the research question.

Reimbursements
We will waive the medical cost associated during your follow-up visit. You will not be given any other money or gifts to take part in this research.

- ***Examples of question to elucidate understanding:*** *Can you tell me if you have understood correctly the benefits that you will have if you take part in the study? Do you have any other questions?*

Confidentiality
We will not be sharing the identity of those participating in the research. The information that we collect from this research project will be kept confidential. Information about you that will be collected during the research will be put away and no-one but the researchers will be able to see it. Any information about you will have a number on it instead of your name. Only the researchers will know what your number is and we will lock that information up in the computer. It will not be shared with or given to anyone except your clinician.

- ***Example of question to elucidate understanding:*** *Did you understand the procedures that we will be using to make sure that any information that we as researchers collect about you will remain confidential? Do you have any questions about them?*

Sharing the Results
The results of this study will be disseminated via peer-reviewed publications and conference presentations. No data are available at the moment. Confidential information will not be shared.

Right to Refuse or Withdraw
You do not have to take part in this research if you do not wish to do so and refusing to participate will not affect your treatment at this hospital in any way. You will still have all the benefits that you would otherwise have at this hospital. You may stop participating in the research at any time that you wish without losing any of your rights as a patient here. Your treatment at this hospital will not be affected in any way.

Alternatives to Participating
If you do not wish to take part in the research, you will be provided with the anterior transversalis fascia repair at the hospital.

Who to Contact
If you have any questions you may ask them now or later, even after the study has started. If you wish to ask questions later, you may contact any of the following:
Name: Dr. Yang
Address: 4# Chong-shan East Road, Shenyang
Telephone number: (024)62255001

E-mail: 529687607@qq.com

This proposal has been reviewed and approved by Ethics Committee of the Fourth Affiliated Hospital of China Medical University, which is a committee whose task it is to make sure that research participants are protected from harm. If you wish to find about more about the IRB, contact [Dr. Yuan-zhe Jin, 4# Chong-shan East Road, Shenyang, 024-62043027.]).

- ***Example of question to elucidate understanding:*** *Do you know that you do not have to take part in this study if you do not wish to? You can say No if you wish to? Do you know that you can ask me questions later, if you wish to? Do you know that I have given the contact details of the person who can give you more information about the study?*

You can ask me any more questions about any part of the research study, if you wish to. Do you have any questions?

PART II: Certificate of Consent

I have read the foregoing information, or it has been read to me. I have had the opportunity to ask questions about it and any questions that I have asked have been answered to my satisfaction. I consent voluntarily to participate as a participant in this research.

Print Name of Participant__________________

Signature of Participant ___________________

Date ___________________________
Day/month/year

If illiterate

A literate witness must sign (if possible, this person should be selected by the participant and should have no connection to the research team). Participants who are illiterate should include their thumb-print as well.

I have witnessed the accurate reading of the consent form to the potential participant, and the individual has had the opportunity to ask questions. I confirm that the individual has given consent freely.

Print name of witness_____________________ **OR** **Thumb print of participant**

Signature of witness ______________________

Date ________________________
Day/month/year

Statement by the researcher/person taking consent

I have accurately read out the information sheet to the potential participant, and to the best of my ability made sure that the participant understands that the inguinal hernia repair will be performed and a few follow-up visits requested to finish the study.

I confirm that the participant was given an opportunity to ask questions about the study, and all the questions asked by the participant have been answered correctly and to the best of my ability. I confirm that the individual has not been coerced into giving consent, and the consent has been given freely and voluntarily.

A copy of this ICF has been provided to the participant.

Print Name of Researcher/person taking the consent________________________

Signature of Researcher /person taking the consent__________________________

Date ___________________________
Day/month/year
