# Supplementary material for: Anterior transversalis fascia approach versus preperitoneal space approach for inguinal hernia repair in residents in northern China: study protocol for a prospective, multicentre, randomised, controlled trial
Source: BMJ Open. 2017 Aug 31;7(8):e016481. doi: 10.1136/bmjopen-2017-016481 (PMC5588954; doi:10.1136/bmjopen-2017-016481)
Supplement: Author's manuscript [file bmjopen-2017-016481.draft_revisions.pdf]

BMJ Open

BMJ Open is committed to open peer review. As part of this commitment we make the peer review history of every article we publish publicly available.

When an article is published we post the peer reviewers' comments and the authors' responses online. We also post the versions of the paper that were used during peer review. These are the versions that the peer review comments apply to.

The versions of the paper that follow are the versions that were submitted during the peer review process. They are not the versions of record or the final published versions. They should not be cited or distributed as the published version of this manuscript.

BMJ Open is an open access journal and the full, final, typeset and author-corrected version of record of the manuscript is available on our site with no access controls, subscription charges or pay-per-view fees (http://bmjopen.bmj.com).

If you have any questions on BMJ Open's open peer review process please email info.bmjopen@bmj.com

BMJ Open

Anterior transversalis fascia approach versus preperitoneal space approach for inguinal hernia repair in residents in Northern China: study protocol for a prospective, multi-center, randomized, controlled trial

Journal:	*BMJ Open*
Manuscript ID	bmjopen-2017-016481
Article Type:	Protocol
Date Submitted by the Author:	18-Feb-2017

Complete List of Authors:	Fan, Qing; The Fourth Affiliated Hospital of China Medical University<br>Zhang, Dewei; The Fourth Affiliated Hospital of China Medical University<br>Yang, Daye; The Fourth Affiliated Hospital of China Medical University<br>Li, Hongwu; The Fourth Affiliated Hospital of China Medical University<br>Wei , Shibo; The Fourth Affiliated Hospital of China Medical University<br>Yang, Liang ; The Fourth Affiliated Hospital of China Medical University<br>Yang, Fuquan ; Shengjing Hospital of China Medical University<br>Zhang, Shaojun ; Fengtian Hospital of Shenyang Medical College<br>Wu, Yaoqiang ; The First Hospital of Dandong City<br>An, Weide ; The First Affiliated Hospital of Dalian Medical University<br>Dai, Zhongshu ; General Hospital of Benxi Steel and Iron (Group) Co., LTD, Fifth Clinical College of China Medical University<br>Jiang, Huiyong ; General Hospital of Shenyang Military Area<br>Wang, Furong ; The 202nd Hospital of PLA<br>Qiao, Shifeng ; The First Affiliated Hospital of Liaoning Medical University<br>Li, Hangyu; The Fourth Affiliated Hospital of China Medical University

**Primary Subject Heading**:	Surgery
Secondary Subject Heading:	Renal medicine
Keywords:	inguinal hernia, anterior transversalis fascia repair, preperitoneal repair, quality of life, cost-utility analysis, randomized

SCHOLARONE™
Manuscripts

Anterior transversalis fascia approach versus preperitoneal space approach for inguinal hernia repair in residents in Northern China: study protocol for a prospective, multi-center, randomized, controlled trial

For peer review only

Qing Fan[1],De-wei Zhang[1],Da-ye Yang[1],Hong-wu Li[1],Shi-bo Wei[1],Liang Yang[1],Fu-quan Yang[2],Shao-jun Zhang[3],Yao-qiang Wu[4],De-wei An[5], Zhong-shu Dai[6], Hui-yong Jiang[7], Fu-rong Wang[8], Shi-feng Qiao[9], Hang-yu Li[1*]

Qing Fan The Fourth Affiliated Hospital of China Medical University, Shenyang,Liaoning Province, China Email: 18900913745@163.com

De-wei Zhang The Fourth Affiliated Hospital of China Medical University, Shenyang,Liaoning Province, China Email: syzhangdewei@sohu.com

Da-ye Yang The Fourth Affiliated Hospital of China Medical University, Shenyang,Liaoning Province, China Email:yangdaye73@126.com

Hong-wu Li The Fourth Affiliated Hospital of China Medical University, Shenyang,Liaoning Province, China Email: 13998201180@163.com

Shi-bo Wei The Fourth Affiliated Hospital of China Medical University, Shenyang,Liaoning Province, China Email: 13898111626@163.com

For peer review only - http://bmjopen.bmj.com/site/about/guidelines.xhtml

Liang Yang The Fourth Affiliated Hospital of China Medical University, Shenyang,Liaoning Province, China Email: 529687607@qq.com

Fu-quan Yang Shengjing Hospital of China Medical University, Shenyang, Liaoning Province, China Email: yangfq@sj-hospital.org

Shao-jun Zhang Fengtian Hospital of Shenyang Medical College, Shenyang, Liaoning Province, China Email: shaojunzhang@163.com

Yao-qiang Wu The First Hospital of Dandong City, Dandong, Liaoning Province, China Email:wuyaoqiang@sina.com

Wei-de An The First Affiliated Hospital of Dalian Medical University, Dalian, Liaoning Province, ChinaEmail: anweide@sina.com

Zhong-shu Dai General Hospital of Benxi Steel and Iron (Group) Co., LTD, Fifth Clinical College of China Medical University, Benxi, Liaoning Province, ChinaEmail: dzs20092009@163.com

Hui-yong Jiang General Hospital of Shenyang Military Area, Shenyang, Liaoning Province, China Email: huiyongj@126.com

Fu-rong Wang The 202nd Hospital of PLA, Shenyang, Liaoning Province, China Email: furong_jiyu@126.com

For peer review only - http://bmjopen.bmj.com/site/about/guidelines.xhtml

Shi-feng Qiao The First Affiliated Hospital of Liaoning Medical University, Jinzhou, Liaoning Province, China Email: shifengqiao2013@163.com

Hang-yu Li* The Fourth Affiliated Hospital of China Medical University, Shenyang,Liaoning Province, China

***Correspondence to**: Hang-yu Li, li_hangyu@126.com.

For peer review only

For peer review only

For peer review only - http://bmjopen.bmj.com/site/about/guidelines.xhtml

Abstract

Introduction: Many surgical techniques have been used to repair abdominal wall defects in the inguinal region based on the anatomic characteristics of this region, and can be categorized as 'tension' repair or 'tension-free' repair. Tension-free repair is the preferred technique for inguinal hernia repair. Tension-free repair of inguinal hernia can be performed through either the anterior transversalis fascia approach or the preperitoneal space approach. There are few large-sample, randomized, controlled trials investigating the curative effects of the anterior transversalis fascia approach versus the preperitoneal space approach for inguinal hernia repair in patients in northern China.

Methods and analysis: This will be a prospective, large-sample, multicenter, randomized, controlled trial. A cohort of over 2,000 patients with inguinal hernias will be recruited from 9 institutions in Liaoning Province, China. Patient randomization will be stratified by center to undergo inguinal hernia repair via the anterior transversalis fascia approach or the preperitoneal approach. Primary and secondary outcome assessments will be performed at baseline (prior to surgery), pre-discharge, and at postoperative 1 week, 1 month, 3 months, 1 year and 2 years. The primary outcome is the percentage of patients with postoperative pain. The secondary outcome is

For peer review only - http://bmjopen.bmj.com/site/about/guidelines.xhtml

For peer review only

postoperative complications (including rates of wound infection, hematoma, seroma, and hernia recurrence). Other outcome measures include quality of life (evaluated by the 36-Item Short Form Health Survey) and cost-utility analysis.

Ethics and dissemination: This trial will be conducted in accordance with the Declaration of Helsinki and supervised by the institutional review board of the Fourth Affiliated Hospital of China Medical University (approval number 2015-027). All patients will receive information about the trial in verbal and written forms and will give informed consent before enrollment. The results will be published in peer-reviewed journals or disseminated through conference presentations.

Trial registration number NCT02984917; Pre-results.

Keywords: inguinal hernia; anterior transversalis fascia repair; preperitoneal repair; quality of life; cost-utility analysis; randomized controlled trial

Article summary

Strengths and limitations of this study

● Based on study results, we aim to effectively reduce physical and psychological pain, ensure high quality medical care (including safety), and achieve the best rehabilitation

For peer review only - http://bmjopen.bmj.com/site/about/guidelines.xhtml

in the treatment of inguinal hernia.

● We aim to determine how to reduce medical resources (including shortening treatment time and reducing labor service strength) and medical costs, and improve the efficiency of medical work and other issues.

● This study will provide important clinical guidance as to the method of inguinal hernia repair thatis most suitable for the anatomic characteristics of patients in northern China and adaptive to the regional economic situation.

● This is a multi-center (over nine research centers), large-sample study; therefore, study conduction in each research center based on the establishment of strict regulations is key to accurate results.

For peer review only

For peer review only - http://bmjopen.bmj.com/site/about/guidelines.xhtml

BACKGROUND

History and current related studies

Inguinal hernia is a common surgical disease that manifests as protrusion of abdominal cavity contents through the inguinal canal because of an abdominal wall defect. It is more common in males than in females, with an overall incidence of 5–10%[1]. Methods for surgical repair of abdominal wall defects in the inguinal region are classified as either 'tension'repairs or 'tension-free' repairs. Herniorrhaphy through repair of the posterior wall of the inguinal canal was first described by Bassini in 1887, and is regarded as a classic surgical method[2].

As understanding of the anatomic location and pathophysical characteristics of inguinal hernia developed, the American surgeon Lichtenstein proposed a new concept of tension-free herniorrhaphy[3]. This technique was quickly adopted worldwide because of its advantages including minimal invasion, technical ease, effectiveness, low complication rate, low recurrence rate, and allowance of resumption of unrestricted physical activity. The most common technique is open tension-free herniorrhaphy.

Many surgical repair methods involving patches (of varying types and materials) are available for inguinal hernia repair.

For peer review only - http://bmjopen.bmj.com/site/about/guidelines.xhtml

Tension-free herniorrhaphy methods include anterior transversalis fascia repair, preperitoneal repair, abdominal cavity patch repair and combined repair approaches[4-6]. Lichtenstein herniorrhaphy is the representative technique of anterior transversalis fascia repair. Preperitoneal repair techniques include transabdominal preperitoneal, total extraperitoneal, and Kugel repair techniques. The combined repair approaches refer to tension-free herniorrhaphy using a modified Kugel patch and the Ultrapro hernia system[7 8].

There are few reports on the effects of different inguinal hernia repair approaches on postoperative complications, particularly regarding severe postoperative complications, in patients in northern China. The incidence of severe complications after inguinal hernia repair is relatively low, but this surgery can lead to physical impairment or organ dysfunction that greatly decreases patient quality of life and places a heavy burden on the patient's family and society. At present, there is a scarcity of clinical evidence from large-sample, randomized, controlled trials investigating the effectiveness of inguinal hernia repair via the anterior transversalis fascia approach versus the preperitoneal approach.

Main objectives

For peer review only - http://bmjopen.bmj.com/site/about/guidelines.xhtml

In this study, we will investigate the advantages and disadvantages of the anterior transversalis fascia approach versus the preperitoneal approach for inguinal hernia repair in residents from northern China regarding common postoperative complications (including acute and chronic pain, wound infection, rates of wound infection, hematoma, seroma, and hernia recurrence) and severe postoperative complications. These outcomes will provide trial-based evidence for selection of rational therapeutic regimen in the clinic.

Distinguishing features from related studies

This study will use center-based stratification to compare the effects of different surgical repair approaches (involving various patch types and materials) on postoperative complications, in an attempt to determine the optimal surgical hernia repair approach that is suitable for the anatomic characteristics of the inguinal region of residents in northern China and corresponds to the regional economic conditions. Cost-utility analysis will be analyzed using center-based stratification. In addition, this study will investigate the effects of different surgical repair approaches on postoperative quality of life.

For peer review only - http://bmjopen.bmj.com/site/about/guidelines.xhtml

Methods/Design

Study design

This is a prospective, large-sample, multi-center, randomized, controlled trial that will include a cohort of over 2,000 patients with inguinal hernia. In strict accordance with the Consolidated Standards of Reporting Trials (CONSORT) standards, the baseline data (preoperative data), therapeutic regimen, therapeutic outcome, and medical costs during hospitalization of patients with inguinal hernia will be recorded. Patient data will be collected using an electronic data capture system (EDC).

According to recommendations for treatment and follow-up of inguinal hernia repair in adults in the Guidelines for the Diagnosis and Treatment of Inguinal Hernia in Adults (2014 Edition) formulated by Chinese scholars[10] and the Adult Inguinal Hernia Treatment Guidelines (Updated Edition in 2014) developed by the European Hernia Society[11], patients who undergo herniorrhaphy will be followed-up at seven time-points: at baseline (at admission, visit 1), pre-discharge, and at 1 week (visit 2), 1 month (visit 3), 3 months (visit 4, clinic visit or telephone follow-up), 1 year (visit 5, telephone follow-up), and 2 years after surgery (visit 6, telephone follow-up). The flow chart of the study protocol is shown in Figure 1. Prior to surgery: patients will be re-screened against inclusion and exclusion criteria. Signed informed consent will be obtained.

For peer review only - http://bmjopen.bmj.com/site/about/guidelines.xhtml

Patient’s demographic data, history of disease and medication, and admission condition and vital signs will be recorded. Clinical examination data will be collected from each center, including history of disease, physical examination, laboratory testing results, imaging findings, preoperative VAS pain score, intraoperative findings, and details of occurrence and management methods of intraoperative injury to the intestinal tract and bladder, spermatic cord, and vascular system.

· Pre-discharge, and 1 week, 1 and 3 months, 1 and 2 years after surgery: pain, wound infection, hematoma and seroma in the inguinal region, and hernia recurrence will be recorded. Medical costs during hospitalization and patient quality of life after discharge will be also recorded. The flow chart of study protocol is shown in Figure 1.

Patients

Patients with inguinal hernia will be recruited from nine trial centers in northern China: the Department of General Surgery, the Fourth Affiliated Hospital of China Medical University; Department of General Surgery, Branch 3, First Hospital of Dalian Medical University; Department of General Surgery, the 202 Hospital of Chinese PLA; Ward of Hernia, Department of General and Gastrointestinal Surgery, First Affiliated Hospital of Liaoning Medical University; Department of General Surgery, General Hospital of

For peer review only - http://bmjopen.bmj.com/site/about/guidelines.xhtml

Shenyang Military; Second Department of General Surgery, General Hospital of Benxin Iron and Steel Co., Ltd.; First Department of General Surgery, Affiliated Central Hospital of Shenyang Medical University; Department of General Surgery, First Hospital of Dandong; Shengjing Hospital of China Medical University.

For peer review only

Inclusion criteria

Male patients presenting with all of the following conditions will be considered for study admission:

· Diagnosed with primary unilateral inguinal hernia

· Aged 18–80 years

· American Society of Anesthesiologists (ASA) classification I–II

· Provision of informed consent

Exclusion criteria

Patients with any one or more of the following will be excluded from this study:

· Severe organ dysfunction or inability to tolerate surgery

For peer review only - http://bmjopen.bmj.com/site/about/guidelines.xhtml

· Hernia recurrence

· Giant hernia (inner size of the hernia > 4 cm)

· Scrotal hernia

· Incarcerated inguinal hernia

· Inability to complete follow-up or questionnaire because of mental disorder or other reasons

· History of preperitoneal surgery, such as radical prostatectomy

Randomization and blinding

Randomization of patients will be stratified by center. Before surgery, each patient will be assigned a serial number by a researcher who will not be involved in the trial using SPSS 19.0 software, and each trial center will be informed of the assignment outcomes. Operation room nurses will record the designated surgical regimen for each patient according to patient's serial number and inform the surgeon. Outcome assessors will be blinded to the surgical records in the electronic case report form (eCRF). Patients will not know the surgical regimen until after the surgery.

For peer review only - http://bmjopen.bmj.com/site/about/guidelines.xhtml

Interventions

Based on recommendations for treatment and follow-up of inguinal hernias in adults made by the Chinese Medical Association and Chinese Medical Doctor Association[10] and the European Hernia Society[11].

Anterior transversalis fascia repair

An oblique skin incision will be made parallel to the inguinal groove from the inner to the outer inguinal ring. Subcutaneous tissue will be dissected until the external oblique aponeurosis is reached. Hemostasis will be achieved by electric coagulation. A4–6 cm long skin incision will be made on the external oblique aponeurosis, starting from the pubic tubercle, to fully expose the pubic tubercle and the inner ring.

The lower part of the external oblique aponeurosis will be separated from the spermatic cord. The upper part of the external oblique aponeurosis will then be separated from the internal oblique aponeurosis and dissociated to a point 3 cm above the inguinal canal wall. The overlying spermatic cord and cremaster muscle will be lifted up and disassociated about 2 cm above the pubic tubercle. The ilioinguinal nerve, external

For peer review only - http://bmjopen.bmj.com/site/about/guidelines.xhtml

spermatic vessels, and genital nerves concomitant with the spermatic cord will be protected while the spermatic cord is lifted.

The inguinal canal will then be dissected and the herniasac will be dissociated from the surrounding tissue. The cremaster muscle fibers will be separated longitudinally and the hernia sac will be separated from the spermatic cord. The hernia sac will be dissociated until the neck of the hernia sac is reached. All abdominal contents in the hernia sac will be returned to the abdominal cavity. In cases with a small hernia sac, the sac will also be returned to the anterior peritoneal cavity to minimize postoperative pain. In cases with a large hernia sac, the distal end will be transected and opened, and the proximal part will be ligated at a high position.

A patch designed with an arc-shaped head and a swallow tail-like end that comprises two pieces (a wide top piece and a narrow bottom piece) will be sutured and fixed with non-absorbable polypropylene sutures. The inner side of the patch will be sutured to the pubic tubercle and the lateral boundary of the rectus abdominis muscle. The upper border of the patch will be sutured to the conjoined tendon and its border with the inguinal ligament and iliopubic tract. The wound will then be closed using layered sutures according to anatomic position.

For peer review only - http://bmjopen.bmj.com/site/about/guidelines.xhtml

Preperitoneal space repair

The skin will be cut and subcutaneous tissue will be dissected. The abdominal external oblique aponeurosis will be dissected along the fibers. The abdominal internal oblique aponeurosis and transversus abdominis muscle will be bluntly separated to expose the transverse fascia. Dissociation of the hernia sac will be done as described above in the anterior transversalis fascia repair.

Transabdominal preperitoneal or totally extraperitoneal laparoscopic techniques were used to dissociate preperitoneal space, strip spermatic cord peritoneum, anatomize and expose the pubic pore. A polypropylene patch will be placed in the prevesical space and Bogros' space to achieve repair of the pubic pore-containing area.

Concomitant treatment

Any medications, with the exception of inguinal hernia-specific treatments, administered during hospitalization will be recorded. Before starting the trial (i.e., at the first visit), detailed information will be recorded regarding concomitant diseases, combined medication and measures to be taken. At discharge, changes in medications and measures to be taken will be recorded. For every combination of medication and

For peer review only - http://bmjopen.bmj.com/site/about/guidelines.xhtml

measures to be taken, a minimum of the following information will be recorded: drug name (generic preferred), dosage, start date, stop date or continuing use, and indications.

Study flowchart

Before surgery

Visit 1 (at admission, day 0)

· Sign informed consent

· Recheck inclusion/exclusion criteria

·Demographic data (sex, age, body height, body mass index) and medical insurance type

· Type of hernia (indirect or direct)

· Inguinal hernia classification as type I, II, III, or IV according to the adult inguinal hernia and femoral hernia classification (revised in 2003) developed by the Hernia and Abdominal Wall Surgery Group, Society of Surgery, Chinese Medical Association[12]

· ASA classification

For peer review only - http://bmjopen.bmj.com/site/about/guidelines.xhtml

· History of diseases and risk factors* (diabetes mellitus, cardiovascular disease, lung disease, peripheral vascular disease, dementia, hypertension, smoking history and alcohol use history) (*optional evaluation items)

·Disease onset and admission (interval from first appearance of the lump, main symptoms)

· Imaging examination (ultrasound, CT)

· Laboratory examination (routine blood testing, coagulation testing, testing of blood glucose, lipids and electrolytes, and hepatic and renal function)

· Vital signs (body temperature, pulse, respiration rate, blood pressure)

·VAS pain score

· Electrocardiography

· Concomitant treatment

The baseline information of patients with inguinal hernia included in this study is shown in Table 1.

Pre-discharge

For peer review only - http://bmjopen.bmj.com/site/about/guidelines.xhtml

Visit 2

· Vital signs (body temperature, pulse, respiration rate, blood pressure)

· VAS pain score

· Laboratory examination (routine blood testing, coagulation testing, testing of blood glucose, lipids and electrolytes, and hepatic and renal function)

· Treatment regimen (inguinal hernia-specific treatment)

Surgical approach and patches (type and fixation method); anesthesia method; drug name (generic preferred), dosage, route, start date, length of drug use will be recorded.

· Medical costs

·Concomitant treatment

Inguinal hernia-specific treatment, maintenance therapy, and drugs used for concomitant treatment.

· Medical costs because of adverse events (AE)

Follow-up

For peer review only - http://bmjopen.bmj.com/site/about/guidelines.xhtml

Visit 3 (1 week after surgery via clinic visit or telephone follow-up)

· VAS pain score

·36-Item Short Form Health Survey (SF-36) score

· Medical costs

· Hernia recurrence (date and severity of recurrence).Recurrence will be diagnosed by the medical institutions, after being alerted by patients that symptoms are present.

· Presence or absence of hematoma and/or seroma

· Wound infection (date and severity of wound infection)

Visit 4 (1 month after surgery via clinic visit or telephone follow-up)

· VAS pain score

·SF-36 score

· Medical costs· Hernia recurrence (date and severity of recurrence). Recurrence will be diagnosed by the medical institutions, after being alerted by patients that symptoms are present.

For peer review only - http://bmjopen.bmj.com/site/about/guidelines.xhtml

· Presence or absence of hematoma and/or seroma

· Wound infection (date and severity of wound infection)

Visit 5 (3 months after surgery via clinic visit or telephone follow-up)

· VAS pain score

·SF-36 score

· Medical costs

· Hernia recurrence (date and severity of recurrence). Recurrence will be diagnosed by the medical institutions, after being alerted by patients that symptoms are present.

· Presence or absence of hematoma and/or seroma

· Wound infection (date and severity of wound infection)

Visit 6 (1 year after surgery via telephone follow-up)

· VAS pain score

·SF-36 score

For peer review only - http://bmjopen.bmj.com/site/about/guidelines.xhtml

· Medical costs· Hernia recurrence (date and severity of recurrence). Recurrence will be diagnosed by the medical institutions, after being alerted by patients that symptoms are present.

· Presence or absence of hematoma and/or seroma

· Wound infection (date and severity of wound infection)

Visit 7 (2 years after surgery via telephone follow-up)

· VAS pain score

·SF-36 score

· Medical costs

· Hernia recurrence (date and severity of recurrence). Recurrence will be diagnosed by the medical institutions, after being alerted by patients that symptoms are present.

· Presence or absence of hematoma and/or seroma

· Wound infection (date and severity of wound infection)

For peer review only - http://bmjopen.bmj.com/site/about/guidelines.xhtml

Outcome measures

·Primary outcome measure

Percentage of patients presenting with postoperative pain (VAS pain score ≥ 1) at baseline (prior to surgery), pre-discharge, and at postoperative 1 week, 1 and 3 months, and 1 and 2 years.VAS is one of the most commonly used tools for assessing pain [13]. It comprises a 10 cm line with 0 at one end, representing no pain, and 10 at the other end, representing the worst pain imaginable; the patient places a mark on the line to indicate the degree of pain that they are experiencing.

·Secondary outcome measures

Postoperative complications including wound infection, rates of hematoma, seroma, hernia recurrence, intestinal obstruction, intestinal fistula, entocele, mesenteric thrombosis, femoral vein thrombosis, ischemic orchitis, painful ejaculation, varicocele and scrotal edema at postoperative 1 week, 1 and 3 months, and 1 and 2 years.

·Other outcome measures

For peer review only - http://bmjopen.bmj.com/site/about/guidelines.xhtml

Quality of life as evaluated by the SF-36[14]. The SF-36 is a 36-item, patient-reported survey of patient health. It consists of eight scaled scores, including vitality, physical functioning, bodily pain, general health perceptions, physical functioning, emotional functioning, social functioning, and mental health. The score of each scale is summed and then standardized according to the formula: standardized score = (actual raw score- lowest possible raw score)/possible raw score range ×100. The total SF-36 score is the standardized score based on the sum of the eight scaled scores. A higher score indicates better quality of life.

The cost-utility analysis of therapeutic regimens involving different surgical approaches will be analyzed. Medical costs consist of direct medical and non-medical costs. The direct medical costs include drug charges, inspection fees, laboratory fees, treatment fees, nursing fees, and bed charges. The direct medical costs during hospitalization will be calculated according to the hospital information system. Direct medical costs during the follow-up period will be reported by patients and/or their relatives. Direct non-medical costs include payments for transportation to receive medical care, cost of nutritional supplementation, and the costs for accompanying family members during the treatment period.

All outcome evaluations will be independently performed by an experienced assessor

For peer review only - http://bmjopen.bmj.com/site/about/guidelines.xhtml

blinded to the treatment regimens. The schedule of outcome measurement assessment is shown in Table 2.

Adverse events and serious adverse events (SAE)

According to the study protocol and clinical judgment, AE/SAE occurring after herniorrhaphy will be reported to the Department of General Surgery, the Fourth Affiliated Hospital of China Medical University.

AE refer to any adverse medical events occurring after herniorrhaphy in patients or clinical subjects. SAE refer to any adverse postoperative medical events involving one or more of the following criteria:

○ Death, irrespective of the cause

○ Life-threatening event

○ Severe or permanent disability or organ dysfunction

○ Hemorrhage

○ Malformation, birth defect

○Hospitalization or extended hospital stay

For peer review only - http://bmjopen.bmj.com/site/about/guidelines.xhtml

○ Re-admission to hospital

○ Recurrence (symptomatic) of inguinal hernia

Causal relationship between surgery and adverse events

The causal relationship between the drugs used and AE will be evaluated by the researchers as: certainly relevant, probably relevant, likely relevant, unlikely relevant and irrelevant (Table 3).

Evaluation criteria for the severity of adverse events

Mild: The patient is aware of symptoms, but symptoms can be tolerated. Symptoms are causing mild discomfort, but not interfering with daily activities.

Moderate: Not affecting daily activities.

Severe: Very painful, causing significant functional impairment or loss of self-care ability, and prohibiting the patient from carrying out daily activities.

The researcher will evaluate the severity of AE according to clinical indices such as laboratory and inspection outcomes, not just based on the subject's direct feelings.

For peer review only - http://bmjopen.bmj.com/site/about/guidelines.xhtml

Adverse event reporting

Reporting time limit for AE will be within 24 hours of onset. In the clinical research period (from the time of signing informed consent to 1–2 years postoperatively), any AE occurring in any patient who received either surgical approach will be properly treated. According to the requirements of this study protocol and clinical evaluation, researchers will fill in the table of *Adverse Events After Herniorrhaphy* and submit this within 24 hours to the Clinical Research Center of Abdominal Wall Hernia, the Fourth Affiliated Hospital of China Medical University, China.

Serious adverse event emergency reporting

Reporting time limit for SAE will be within 24 hours of onset. Any SAE will be continuously monitored and reported until it is healed, stabilizes or recovers to near baseline conditions, irrespective of whether patients have terminated or completed treatment. Any follow-up information regarding SAE will be reported within 24 hours.

Patient completion/withdrawal from clinical study

For peer review only - http://bmjopen.bmj.com/site/about/guidelines.xhtml

Patients for whom the whole clinical data of at least 1 year are collected will be considered as complete cases. Patients with any one or more of the following criteria will be considered withdrawn from the study: mistakenly recruited, withdrawal of informed consent, upon the request of the sponsor for safety reasons or patient conflicts, or lost to follow-up. The date and reasons for withdrawal will be recorded on the eCRF. After termination of the study, the data collected at the last visit will be evaluated, except for data from those lost to follow-up.

Statistical analysis

Descriptive statistics will be used to analyze the data. For measurement data, the number of patients (number of missing patients), mean, median, standard deviation, first quartile, third quartile, and maximum and minimum will be described, and 95% confidence intervals will be calculated. For counted data, the frequency and relative numbers will be described, and 95% confidence intervals will be calculated. According to whether the differences between scores before and after treatments are normally distributed, the paired *t*-test or Wilcoxon signed rank test will be used for comparison of measurement data in the same group. The chi-squared test or Fisher's exact test will be used for analysis of counted data, and the Wilcoxon signed rank test will be used for

For peer review only - http://bmjopen.bmj.com/site/about/guidelines.xhtml

analysis of ranked data. The last-observation-carried-forward imputation method will be used when the effects-related visit data at the last visit are missing.

Full analysis set

According to the intention-to-treat principle, patients who receive at least one treatment according to study protocol and undergo at least one evaluation regarding curative effects after baseline evaluation will be included in the full analysis set.

Curative effects analysis

Full analysis set analysis of curative effects will be performed.

The proportions of patients with no complications, patients with common complications, and patients with severe complications within postoperative 3 weeks will be analyzed using descriptive statistics. The chi-squared test or Fisher's exact test will be used for comparison of differences between groups. If statistical differences exist between groups, the Bonferroni method will be used to adjust the α value, and pairwise comparisons will be made.

For peer review only - http://bmjopen.bmj.com/site/about/guidelines.xhtml

If the change in VAS pain score after treatment meets the normality and homogeneity of variance, analysis of variance will be performed. If the statistical analysis of variance results are significant, in-depth statistical analysis will be performed, and the Bonferroni method will be used for pairwise comparisons. Otherwise, the Kruskal-Wallis test will be performed. If the statistical results of the Kruskal-Wallis test are significant, in-depth statistical analysis will be performed, and the independent Wilcoxon signed rank test will be performed. The Bonferroni method will be used to adjust *P* values for pairwise comparisons.

Safety evaluation

Safety analysis and analysis of the incidence of postoperative complications will be performed using descriptive statistics. The chi-squared test or Fisher's exact test will be performed for comparisons between groups. If significant differences exist, the Bonferroni method will be used to adjust the α value, and in-depth pairwise comparisons will be made between groups. Vital signs and laboratory outcomes will be analyzed using descriptive statistics. The paired *t*-test or Wilcoxon signed rank test will be used to analyze the differences between values before and after surgery. Imaging data and electrocardiograms will be analyzed using cross tabulations.

For peer review only - http://bmjopen.bmj.com/site/about/guidelines.xhtml

Economic analysis

Cost-utility analysis will be used for economic evaluation, and sensitivity analysis of cost and utility will be performed.

Interim analysis

When an adequate number of patients are enrolled and followed-up, interim analysis will be performed. When data are included for the full analysis set and recorded in the database, the first interim analysis during the management period will be performed to check whether the core data collected are suitable for preliminary significant data analysis. According to research progression, subsequent interim analysis of all data included in the database will then be designated. After acquiring approval from the Department of General Surgery and Scientific Construction Committee, the Fourth Affiliated Hospital of China Medical University, the data collected in the Department of General Surgery, the Fourth Affiliated Hospital of China Medical University are likely to be analyzed together with the data collected from the other research centers. In accordance with applicable laws and regulations, the information on the subjects in the

For peer review only - http://bmjopen.bmj.com/site/about/guidelines.xhtml

study will be kept confidential. The data for interim analysis will be precisely described in a statistical analysis plan. The interim analysis results will be submitted to the sponsor and the project manager in the form of a statistical analysis report, and as slides by the co-sponsors.

Sample size

We hypothesized that anterior transversalis fascia repair can reduce the percentage of patients with postoperative pain by 15% compared with the percentage of patients with pain before surgery. Taking $\alpha = 0.05$ and power = 80%, the final effective sample size of n = 300 was calculated. Assuming a patient loss rate of 20%, we require 360 patients. This study is planned to be performed in more than nine institutes to investigate the effectiveness of inguinal hernia repair via the anterior transversalis fascia approach versus the preperitoneal approach in patients in northern China. We plan to include at least 2,000 patients.

Ethical Approval

Before study commencement, the following files will be provided to the Independent

For peer review only - http://bmjopen.bmj.com/site/about/guidelines.xhtml

ethics committee (IEC):

○ Final draft of study protocol (and supplements)

○ Sponsor-approved informed consent and other documents provided to the subjects (such as participation card and diary card)

○ Materials assisting patients to be included

○ Materials regarding study-related injury compensations or rewards for patient participation in the study

○ Researcher résumé or equivalent (unless the IEC states that this is not needed)

○ Sponsor name, funds, potential competing interests, and information that affects patient participation in the study

○Any other documents required by the IEC

Trials cannot be started until the IEC completely approves the study protocol, informed consent, materials assisting patients to be included, and compensation measures for the patients, and the sponsor receives a copy of the IEC approval document. The IEC approval document should include the trial title (registration number), name of the study

For peer review only - http://bmjopen.bmj.com/site/about/guidelines.xhtml

file (including edition code) and date of approval.

During the study period, it is likely that researchers will submit the following files to the IEC for approval at appropriate time-points:

○ Supplement of study protocol

○ Informed consent forms and documents regarding rewards for patient participation in the study

○ New information that is likely to negatively affect participant safety and study progression

○ Files regarding bias and alterations of the study protocol made to avoid immediate injury to patients

○ Reports regarding dead patients

○ Notification of change of project manager

○ Other requirements of the IEC

If the supplemented study protocol increases the risk to patients, the supplemented

For peer review only - http://bmjopen.bmj.com/site/about/guidelines.xhtml

study protocol and corresponding modified informed consent form will be submitted to the IEC for consideration. The supplemented study protocol will not be performed until approval from the IEC is obtained. The major study protocol was approved by the IEC, the Fourth Affiliated Hospital of China Medical University (approval number 2015-027) on November 27, 2015.The study protocol should be reviewed by the IEC at least once every year, and the reviewed suggestion will be recorded on paper. At the end of the study, the researchers should inform the IEC of its completion.

Informed Consent to participate

Each patient (or his/her legal representative) will provide signed and dated informed consent before surgery after fully understanding the objective and contents of the study. The researcher or his/her authorized staff members will fully explain the objective, methods, possible benefits, potential risks and any possible discomforts of the study to the potential patients before inclusion. Participants will be informed that participation in the study is voluntary and that they can withdraw from the study at any time. The participants will know that their identifying information will be recorded for long-term follow-up, and will be read by personnel from the related institutions and the sponsor within the permit of relevant laws and regulations. The right to privacy of the participant

For peer review only - http://bmjopen.bmj.com/site/about/guidelines.xhtml

will be protected.

Confidentiality

○ Only data required to investigate the effectiveness and safety of herniorrhaphy will be collected and analyzed.

○ Data collection and use will not be disclosed to any non-authorized persons, and will be performed in accordance with the laws and regulations regarding protection of the participant's privacy.

○ The process of data collection will be fair and lawful.

○ The purpose of data collection will be specific, identified and legitimate, and the collected data will not be used for other unrelated objectives.

○ The data collected will be adequate, related and not redundant relative to the study objective.

○ The data collected will be accurate and updated when necessary.

○ Before collection of personal data, researchers will obtain participant consent, which should include lay emphasis on the transfer of data to other institutional entities and

For peer review only - http://bmjopen.bmj.com/site/about/guidelines.xhtml

countries.

○ The participants have the right to obtain their data and can request to modify mistaken or incomplete data.

○ During the study period, participant's personal information will not be obtained or disclosed to non-authorized persons, and will not be illegally destroyed, lost or altered unexpectedly. During the entire study period, the sponsors who have the right to read the participant's personal information will keep the data confidential.

Data management

Protocol modification

Study protocol modification will be signed, dated, and published by the Department of General Surgery, the Fourth Affiliated Hospital of China Medical University. The study protocol will not be put into clinical practice until IEC approval is received, unless this is necessary to avoid risk to participants, or to modify the study protocol regarding logistics and administration (for example, typographical errors and contradictions).

The study protocol should not be deviated from during clinical practice. When deviation exists, corresponding management will be performed. The causes and deviated

For peer review only - http://bmjopen.bmj.com/site/about/guidelines.xhtml

contents will be recorded in the eCRF and original medical case notes. The study protocol deviation table and eCRF will be preserved in the research center and sponsor institute.

Participant identity registration and screening records

Participants must agree to fill in identification registration to enable individual identification of each participant. The monitor will recheck the integrity of this registration. The participant identity registration form will be confidential, and will be preserved in the research center. To ensure confidentiality, duplication of participant identity registration will be not permitted. All reports and letters relating to this study will be tagged with the relevant acronyms and serial number. The participant screening record form will be completed by doctors. The doctors will determine whether participants are eligible for admission to this study.

Electronic case report form

In this study, the EDC will be used for data collection and management. All data relating to this study will be recorded on the eCRF provided by the sponsor. The

For peer review only - http://bmjopen.bmj.com/site/about/guidelines.xhtml

researchers will fill in the eCRF after each participant visit, unless some clinical results cannot be acquired immediately. This ensures that the information recorded on the eCRF reflects the participant's latest outcome. Data accuracy will be performed by the researcher. Data recording, alteration and substitution will be performed by researchers or other authorized persons. All data will be inputted into the EDC, and data queries will be made by researchers online via the EDC. The final data will not be altered, and will be password-protected.

Data quality assurance

To ensure data accuracy and reliability, eligible researchers and appropriate research centers will be selected before study commencement. The monitor in the co-sponsor research center will monitor the study progression periodically. The co-sponsor will advise the researchers how to fill in the eCRF. The monitor in the co-sponsor research center will visit the EDC to check the integrity and accuracy of the eCRF. Data recorded in the eCRF that is inconsistent with original data recorded will be altered by researchers or authorized persons.

For peer review only - http://bmjopen.bmj.com/site/about/guidelines.xhtml

Auditing

Regular on-site inspection visits will be made by the co-sponsors. The co-sponsors monitor will date the inspection on an inspection form, which will then be preserved in the research center. After study commencement, the first on-site inspection visit will be performed as soon as possible after participant recruitment. During on-site inspection, the monitor will check the consistency of data recorded in the eCRF with original data recorded in medical notes from the research center. The nature and preservation place of original data documents will be confirmed to enable clinical researchers to know the source of all original data required in the eCRF, thus the monitor of the co-sponsor can recheck these data.

If original data are electronically preserved, the monitor of the co-sponsor will discuss the recheck method with clinical researchers. The original data document will include participant identity, eligibility for inclusion, informed consent, dates of visits, execution of study protocol, curative effects, safety index, AE reporting and follow-up, medication, and date of study completion.

The monitor of the co-sponsor will discuss the detailed requirements for original data recording with clinical researchers. To recheck whether the data recorded in eCRF is consistent with original data, the monitor of the co-sponsor will be provided with the

For peer review only - http://bmjopen.bmj.com/site/about/guidelines.xhtml

required original data. The monitor will discuss any problems found during rechecking of data consistency with clinical researchers. The clinical researchers will regularly discuss the information feedback.

Study completion/termination

Study completion

When the last visit of the last participant is completed, the research center will inform the sponsor, and study completion will be designated. The sponsor will inform all research centers of the time of study completion. Further research after this time must be approved by the sponsor and can then be performed without protocol supplements.

Study termination

The sponsor will have the right to terminate the study at any research center at any time possibly because of, but not limited to, the following criteria:

○ The number of patients recruited reaches the predetermined requirements

○ Research cannot abide by the study protocol or GCP guidelines

For peer review only - http://bmjopen.bmj.com/site/about/guidelines.xhtml

○Insufficient numbers of participants are recruited

Audit and inspection

A representative of the department of clinical quality assurance of the co-sponsor may visit any of the research centers to determine whether the study protocol follows the laws and regulations. All study records, including original medical notes, will be disclosed to the representative. However, the privacy of the subject will be respected. The research center will be informed about this visit in advance to allow sufficient time for appropriate preparation.

Data use and publication

Any unpublished information provided by the sponsors, and all unpublished data relating to this study will be kept confidential and will be owned by the sponsor. This information or data will be not be used for other purposes unless written approval is acquired from the sponsor. The clinical researchers will be informed that study results will be used for further study. Therefore, the study results may be provided to other clinical researchers or related administrative departments. The study results will be

For peer review only - http://bmjopen.bmj.com/site/about/guidelines.xhtml

disclosed in the form of a Clinical Study Report, including data collected from any research center involved. If clinical researchers publish the study outcomes, they will provide the original manuscript to the sponsor for online review 60 days before submission or presentation. A summary, posters or other promotional materials will be created to facilitate the review. The sponsor will discuss scientific and regulatory compliance issues with clinical researchers. The sponsor will not mandatorily require the clinical researchers to modify the scientific contents, and has no right to hide information. The clinical researchers should consider the integrity of this multi-center study. The data from one research center can be published only under the following circumstances: the articles involving the outcomes from all research centers have been published; study in all research centers has been accomplished, abandoned or terminated for 12 months; the sponsor has stated that they will not publish the study outcomes from multiple research centers. Assignment of the author listings in articles related to this study will be performed based on the author's contribution guidelines, such as the guidelines of Uniform Requirements for Manuscripts Submitted to medical journals.

DISCUSSION

For peer review only - http://bmjopen.bmj.com/site/about/guidelines.xhtml

Significance of this study

This study will be the first large sample (over 2,000 patients), multi-center, randomized, controlled, clinical trial to investigate the effectiveness of inguinal hernia repair via the anterior transversalis fascia approach versus the preperitoneal approach in patients from northern China. In this study, postoperative complications will be used as the primary outcome measure, and patient quality of life and cost-utility analysis will be the secondary outcome measures.

Trial status

Recruitment of patients is ongoing at the time of submission.

Abbreviations

AE	adverse event

For peer review only - http://bmjopen.bmj.com/site/about/guidelines.xhtml

eCRF	electronic case report form
EDC	electronic data capture
IEC	independent ethics committee
SAE	serious adverse event
CUA	Cost utility analysis

For peer review only

For peer review only - http://bmjopen.bmj.com/site/about/guidelines.xhtml

References

1. Beddy P, Ridgway PF, Geoghegan T, *et al*. Inguinal hernia repair protects testicular function: a prospective study of open and laparoscopic herniorraphy. *J Am Coll Surg*. 2006;203:17-23.

2. Schumpelick V, Treutner KH, Arlt G. Inguinal hernia repair in adults. *Lancet*. 1994;344:375-379.

3. Lichtenstein IL, Shulman AG, Amid PK, *et al*. The tension-free hernioplasty. *Am J Surg* 1989;157:188–193.

4. Bittner R, Arregui ME, Bisgaard T, *et al*. Guidelines for laparoscopic (TAPP) and endoscopic (TEP) treatment of inguinal hernia [International Endohernia Society (IEHS)]. *Surg Endosc*. 2011;25:2773-843.

5. Carter J, Duh QY. Laparoscopic repair of inguinal hernias. *World J Surg*. 2011;35:1519-25.

6. Greater China Endo Hernia Institute; Laparoscopic and Endoscopic Group, Society of Surgery, Chinese Medical Association; Hernia and Abdominal Wall Surgery Group, Society of Surgery, Chinese Medical Association.Guidelines for Standardization of Laparoscopic Surgery for Inguinal Hernia.*Chin J Hernia Abdominal Wall Surg*

For peer review only - http://bmjopen.bmj.com/site/about/guidelines.xhtml

For peer review only

(Electronic). 2013;7:505-512..

7. Karateke F, Ozyazici S, Menekse E, *et al*. ULTRAPRO Hernia System versus lichtenstein repair in treatment of primary inguinal hernias: a prospective randomized controlled study. *Int Surg*. 2014;99:391-397.

8. Kugel RD. The Kugel repair for groin hernias. *Surg Clin North Am*. 2003;83:1119-1139.

9. CONSORT Group (2008) CONSORT: Consolidated Standards of Reporting Trials.

10. Abdominal Wall Surgery Group, Society of Surgery, Chinese Medical Association. Guidelines for the Diagnosis and Treatment of Inguinal Hernia in Adults (2014 Edition).*Chin J Prac Surg*. 2014;52:484-486.

11. Miserez M, Peeters E, Aufenacker T, *et al*. Update with level 1 studies of the European Hernia Society guidelines on the treatment of inguinal hernia in adult patients. *Hernia*. 2014;18:151-63.

12. Hernia Abdominal Wall Surgery Group, Society of Surgery, Chinese Medical Association.Adult inguinal hernia and femoral hernia classification (revised in 2003). *Chin J Gen Surg*. 2004;19:126.

13. Fujita F, Lahmann B, Otsuka K, *et al*. Quantification of pain and satisfaction

For peer review only - http://bmjopen.bmj.com/site/about/guidelines.xhtml

following laparoscopic and open hernia repair. *Arch Surg* 2004,139:596–600.

14. Ware JE Jr, Sherbourne CD. The MOS 36-item short-form health survey (SF-36). I. Conceptual framework and item selection. *Med Care*. 1992,30:473–483.

For peer review only

For peer review only - http://bmjopen.bmj.com/site/about/guidelines.xhtml

Authors' contributions

HYL,FQY, SJZ, YQW, WDA,ZSD,HYJ,FRW and SFQ conceived the study and participated in its design and coordination. LY drafted the manuscript. SBW and QF participated in the design of the study and performed the statistical analysis. DWZ and DYY participated in the study design and coordination and helped draft the manuscript. HWL participated in the design of the study and wrote the protocol for the analysis. All authors read, revised and approved the final manuscript.

Funding Statement

This research was funded by National Natural Science Foundation NO.81472302.

Competing interests Statement

The authors declare that they have no competing interests.

Data sharing statement

The results of this study will be disseminated via peer-reviewed publications and conference presentations. No data are available at the moment.

For peer review only - http://bmjopen.bmj.com/site/about/guidelines.xhtml

For peer review only

For peer review only - http://bmjopen.bmj.com/site/about/guidelines.xhtml

Figure 1 Flow chart of study protocol.

VAS: Visual Analogue Scale; SF-36: 36-Item Short Form Health Survey.

Tables

Table 1 Baseline information of patients with inguinal hernia

Table 2 Timing of outcome measurement assessment

Table 3 Causal relationship between surgery and adverse events

Additional file 1: SPIRIT Checklist

Additional file 2. Trial committee organization and contributions and role. Description of data: trial committee organization and contributions and role in accordance with SPIRIT (Standard Protocol Items: Recommendations for Interventional Trials) 2013 Checklist.

Additional file 3: Informed Consent to participate

Additional file 4: List of ethical approval documents

For peer review only - http://bmjopen.bmj.com/site/about/guidelines.xhtml

For peer review only

For peer review only - http://bmjopen.bmj.com/site/about/guidelines.xhtml

Table 1 Baseline information of patients with inguinal hernia

Sex	Smoking history
Age	History of alcohol use
Body height	Disease attack and admission
Body mass index	Laboratory examination
Medical insurance type	Imaging examination
Type of hernia	Vital sign
Inguinal hernia classification	Visual Analogue Scale (VAS) pain score
Treatment time	Electrocardiography
American Society of Anesthesiologist	Concomitant therapy
Classification	
History of diseases	
Diabetes mellitus	
Cardiovascular disease	
Lung disease	
Peripheral vascular disease	
Dementia	
Hypertension	

For peer review only

Table 2 Timing of outcome measurement assessment

	Before surgery		During surgery		Follow up	
	Visit 1 (at admission, day 0)	Visit 2 (at discharge)	Visit 3 (1 week after surgery)	Visit 4 (1 month after surgery)	Visit 5 (3 months after surgery)	Visit 6 (1 year after surgery)	Visit 7 (3 years after surgery)
Signed informed consent	X						
Inclusion/exclusion criteria	X						
Demographic data	X						
Medical insurance type	X						
Delayed visit and admission	X						
Previous history of diseases	X						
Previous history of drug	X						
Risk factors	X						
Disease attack and admission [a]	X						
Type of hernia (indirect hernia, direct hernia)	X						
Vital sign[b]	X						
Laboratory examination [c]	X	X					
Imaging	X						

For peer review only

examination [d]							
Electrocardiography	X						
Treatment regimen [e]		X					
VAS score [f]	X	X	X	X	X	X	X
SF-36 score			X	X	X	X	X
Medical cost		X	X	X	X	X	X
Concomitant treatment	X	X					
Adverse events<br>Wound infection<br>Hematoma<br>Seroma<br>Hernia recurrence		X	X	X	X	X	X

[a] indicates the interval from the first appearance of the lump or main symptoms; [b] indicates body temperature, pulse, respiration rate, and blood pressure; [c] indicates routine blood testing, coagulation testing, testing of blood glucose, lipids, and electrolytes, and hepatic and renal function; [d] indicates ultrasound, CT examination; [e] indicates inguinal hernia-specific treatment; f indicates the VAS pain score.

For peer review only

Table 3 Causal relationship between surgery and adverse events

	Certainly relevant	Probably relevant	Likely relevant	Unlikely relevant	Irrelevant
Adverse events are obviously caused by external factors	-	-	-	-	+
Adverse events are correlated with surgical treatment at rational time	+	+	+	-	-
Adverse events are correlated with patient diseases	-	-	+	+	+
Adverse events are correlated with suspected postoperative response patterns	+	+/-	+	-	-
After relief of related surgical factors, adverse events alleviate or disappear	+	+	-	-	-
After surgery-related factors worsen, adverse events recur	+	+	-	-	-

Note: To minimize the surgical risk and meet the requirements of laws and regulations, the sponsor will manage the correlations as follows: “Irrelevant” belongs to the irrelevant category, and “certainly relevant”, “probably relevant”, “likely relevant” and “unlikely relevant” belong to the relevant category.

For peer review only

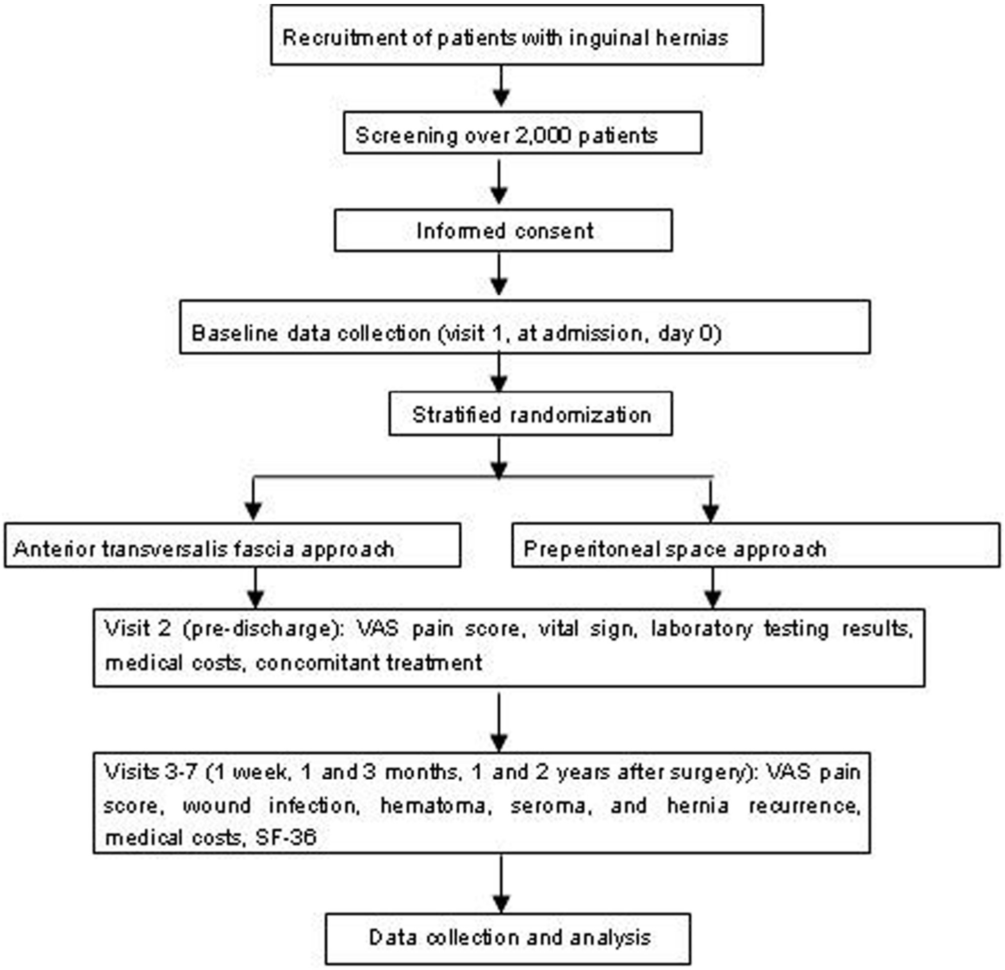

Flow chart of study protocol

116x112mm (300 x 300 DPI)

STANDARD PROTOCOL ITEMS: RECOMMENDATIONS FOR INTERVENTIONAL TRIALS

SPIRIT 2013 Checklist: Recommended items to address in a clinical trial protocol and related documents*

Section/item	Item No	Description	Addressed on page number
**Administrative information**			
Title	1	Descriptive title identifying the study design, population, interventions, and, if applicable, trial acronym	p. 1
Trial registration	2a	Trial identifier and registry name. If not yet registered, name of intended registry	p. 5
	2b	All items from the World Health Organization Trial Registration Data Set	N/A
Protocol version	3	Date and version identifier	p. 33
Funding	4	Sources and types of financial, material, and other support	p. 43
Roles and responsibilities	5a	Names, affiliations, and roles of protocol contributors	p. 43
	5b	Name and contact information for the trial sponsor	p. 43
	5c	Role of study sponsor and funders, if any, in study design; collection, management, analysis, and interpretation of data; writing of the report; and the decision to submit the report for publication, including whether they will have ultimate authority over any of these activities	p. 43
	5d	Composition, roles, and responsibilities of the coordinating centre, steering committee, endpoint adjudication committee, data management team, and other individuals or groups overseeing the trial, if applicable (see Item 21a for data monitoring committee)	Additional file 2
**Introduction**			

For peer review only - http://bmjopen.bmj.com/site/about/guidelines.xhtml

For peer review only

Background and rationale	6a	Description of research question and justification for undertaking the trial, including summary of relevant studies (published and unpublished) examining benefits and harms for each intervention	p. 7
	6b	Explanation for choice of comparators	p. 9
Objectives	7	Specific objectives or hypotheses	p. 8-9
Trial design	8	Description of trial design including type of trial (eg, parallel group, crossover, factorial, single group), allocation ratio, and framework (eg, superiority, equivalence, noninferiority, exploratory)	p. 9-11
**Methods: Participants, interventions, and outcomes**			
Study setting	9	Description of study settings (eg, community clinic, academic hospital) and list of countries where data will be collected. Reference to where list of study sites can be obtained	p. 9
Eligibility criteria	10	Inclusion and exclusion criteria for participants. If applicable, eligibility criteria for study centres and individuals who will perform the interventions (eg, surgeons, psychotherapists)	p. 11-12
Interventions	11a	Interventions for each group with sufficient detail to allow replication, including how and when they will be administered	p. 13
	11b	Criteria for discontinuing or modifying allocated interventions for a given trial participant (eg, drug dose change in response to harms, participant request, or improving/worsening disease)	p. 13
	11c	Strategies to improve adherence to intervention protocols, and any procedures for monitoring adherence (eg, drug tablet return, laboratory tests)	p. 13
	11d	Relevant concomitant care and interventions that are permitted or prohibited during the trial	p. 15-16
Outcomes	12	Primary, secondary, and other outcomes, including the specific measurement variable (eg, systolic blood pressure), analysis metric (eg, change from baseline, final value, time to event), method of aggregation (eg, median, proportion), and time point for each outcome. Explanation of the clinical relevance of chosen efficacy and harm outcomes is strongly recommended	p. 21-22
Participant timeline	13	Time schedule of enrolment, interventions (including any run-ins and washouts), assessments, and visits for participants. A schematic diagram is highly recommended (see Figure)	p. 16-20, Figure 1

For peer review only - http://bmjopen.bmj.com/site/about/guidelines.xhtml

Sample size	14	Estimated number of participants needed to achieve study objectives and how it was determined, including clinical and statistical assumptions supporting any sample size calculations	p. 29
Recruitment	15	Strategies for achieving adequate participant enrolment to reach target sample size	p. 11
**Methods: Assignment of interventions (for controlled trials)**			
Allocation:			
Sequence generation	16a	Method of generating the allocation sequence (eg, computer-generated random numbers), and list of any factors for stratification. To reduce predictability of a random sequence, details of any planned restriction (eg, blocking) should be provided in a separate document that is unavailable to those who enrol participants or assign interventions	p. 12
Allocation concealment mechanism	16b	Mechanism of implementing the allocation sequence (eg, central telephone; sequentially numbered, opaque, sealed envelopes), describing any steps to conceal the sequence until interventions are assigned	p. 12
Implementation	16c	Who will generate the allocation sequence, who will enrol participants, and who will assign participants to interventions	p.12
Blinding (masking)	17a	Who will be blinded after assignment to interventions (eg, trial participants, care providers, outcome assessors, data analysts), and how	p. 13
	17b	If blinded, circumstances under which unblinding is permissible, and procedure for revealing a participant's allocated intervention during the trial	p. 13
**Methods: Data collection, management, and analysis**			
Data collection methods	18a	Plans for assessment and collection of outcome, baseline, and other trial data, including any related processes to promote data quality (eg, duplicate measurements, training of assessors) and a description of study instruments (eg, questionnaires, laboratory tests) along with their reliability and validity, if known. Reference to where data collection forms can be found, if not in the protocol	p. 33-34
	18b	Plans to promote participant retention and complete follow-up, including list of any outcome data to be collected for participants who discontinue or deviate from intervention protocols	p. 32

For peer review only - http://bmjopen.bmj.com/site/about/guidelines.xhtml

Data management	19	Plans for data entry, coding, security, and storage, including any related processes to promote data quality (eg, double data entry; range checks for data values). Reference to where details of data management procedures can be found, if not in the protocol	p. 33-34
Statistical methods	20a	Statistical methods for analysing primary and secondary outcomes. Reference to where other details of the statistical analysis plan can be found, if not in the protocol	p. 25-26
	20b	Methods for any additional analyses (eg, subgroup and adjusted analyses)	p. 26
	20c	Definition of analysis population relating to protocol non-adherence (eg, as randomised analysis), and any statistical methods to handle missing data (eg, multiple imputation)	p. 26
**Methods: Monitoring**			
Data monitoring	21a	Composition of data monitoring committee (DMC); summary of its role and reporting structure; statement of whether it is independent from the sponsor and competing interests; and reference to where further details about its charter can be found, if not in the protocol. Alternatively, an explanation of why a DMC is not needed	p. 32
	21b	Description of any interim analyses and stopping guidelines, including who will have access to these interim results and make the final decision to terminate the trial	p. 27
Harms	22	Plans for collecting, assessing, reporting, and managing solicited and spontaneously reported adverse events and other unintended effects of trial interventions or trial conduct	p. 24
Auditing	23	Frequency and procedures for auditing trial conduct, if any, and whether the process will be independent from investigators and the sponsor	p. 36
**Ethics and dissemination**			
Research ethics approval	24	Plans for seeking research ethics committee/institutional review board (REC/IRB) approval	p. 29-31, Additional file 4
Protocol amendments	25	Plans for communicating important protocol modifications (eg, changes to eligibility criteria, outcomes, analyses) to relevant parties (eg, investigators, REC/IRBs, trial participants, trial registries, journals, regulators)	p. 33-34

For peer review only - http://bmjopen.bmj.com/site/about/guidelines.xhtml

Consent or assent	26a	Who will obtain informed consent or assent from potential trial participants or authorised surrogates, and how (see Item 32)	p. 32
	26b	Additional consent provisions for collection and use of participant data and biological specimens in ancillary studies, if applicable	N/A
Confidentiality	27	How personal information about potential and enrolled participants will be collected, shared, and maintained in order to protect confidentiality before, during, and after the trial	p. 32
Declaration of interests	28	Financial and other competing interests for principal investigators for the overall trial and each study site	p. 43
Access to data	29	Statement of who will have access to the final trial dataset, and disclosure of contractual agreements that limit such access for investigators	p. 43
Ancillary and post-trial care	30	Provisions, if any, for ancillary and post-trial care, and for compensation to those who suffer harm from trial participation	p. 25
Dissemination policy	31a	Plans for investigators and sponsor to communicate trial results to participants, healthcare professionals, the public, and other relevant groups (eg, via publication, reporting in results databases, or other data sharing arrangements), including any publication restrictions	p. 37-38
	31b	Authorship eligibility guidelines and any intended use of professional writers	N/A
	31c	Plans, if any, for granting public access to the full protocol, participant-level dataset, and statistical code	N/A
**Appendices**			
Informed consent materials	32	Model consent form and other related documentation given to participants and authorised surrogates	Additional file 3
Biological specimens	33	Plans for collection, laboratory evaluation, and storage of biological specimens for genetic or molecular analysis in the current trial and for future use in ancillary studies, if applicable	p. 9

*It is strongly recommended that this checklist be read in conjunction with the SPIRIT 2013 Explanation & Elaboration for important clarification on the items. Amendments to the protocol should be tracked and dated. The SPIRIT checklist is copyrighted by the SPIRIT Group under the Creative Commons "Attribution-NonCommercial-NoDerivs 3.0 Unported" license.

For peer review only - http://bmjopen.bmj.com/site/about/guidelines.xhtml

Principal investigator (PI) and research physicians at main study site

Organization: the Fourth Affiliated Hospital of China Medical University

Contributions and role: Principal investigator, original study design: Hang-yu Li (Email:li_hangyu@126.com)

Organization of Steering committee and member appointment: Qing Fan, De-wei Zhang, Da-ye Yang, Hong-wu Li, Shi-bo Wei, Liang Yang, Hang-yu Li, Fu-quan Yang, Shao-jun Zhang, Yao-qiang Wu, De-wei An, Zhong-shu Dai, Hui-yong Jiang, Fu-rong Wang, Shi-feng Qiao

Communication and exchange of opinion with PI at each site

Preparation of IRB documents and CRF

Trial management (randomized allocation management, AE data collection at each site, participant enrollment supervision, study site inspection and visits, budget allocation and management)

Cooperation with CRO in data collection, quality control, monitoring, and analysis

Steering committee (SC)

Organization and role: All authors of this manuscript

Contributions: Protocol revision and decision on final protocol

Organization of Trial Management Committee and member appointment

Designation of participant recruitment study sites

Inspection of study progress, and decision on protocol revision, if needed

Determination of study result publication timing and method

Decision on authorship in accordance with Authorship eligibility guidelines

Trial Management Committee

Organization: PI and investigators at each clinical trial participant enrollment site

Fu-quan Yang, Shengjing Hospital of China Medical University

For peer review only - http://bmjopen.bmj.com/site/about/guidelines.xhtml

Shao-jun Zhang, Fengtian Hospital of Shenyang Medical College

Yao-qiang Wu, The First Hospital of Dandong City

Wei-de An, The First Affiliated Hospital of Dalian Medical University

Zhong-shu Dai, General Hospital of Benxi Steel and Iron (Group) Co., LTD, Fifth Clinical College of China Medical University

Hui-yong Jiang, General Hospital of Shenyang Military Area

Fu-rong Wang, The 202nd Hospital of PLA

Shi-feng Qiao, The First Affiliated Hospital of Liaoning Medical University

Hang-yu Li*, The Fourth Affiliated Hospital of China Medical University

Organization and role: Submission and obtaining study protocol approval from relevant IRB of each study site

Clinical trial execution following protocol (e.g. participant recruitment, enrollment, data collection, CRF entry)

Collection and report of AEs

For peer review only

For peer review only - http://bmjopen.bmj.com/site/about/guidelines.xhtml

Ethics Committee of the Fourth Affiliated Hospital of China Medical University

4# CHONG-SHAN EAST ROAD, SHENYANG, P.R.CHINA 110032

Informed Consent Form for Clinical Studies

[Name of Principal Investigator] Hang-yu Li
[Name of Organization] The Fourth Affiliated Hospital of China Medical University, China
[Name of Proposal and version] Anterior transversalis fascia approach versus preperitoneal space approach for inguinal hernia repair in residents in Northern China: study protocol for a prospective, multi-center, randomized, controlled trial (Version 1.0)

This Informed Consent Form has two parts:

- **Information Sheet (to share information about the research with you)**
- **Certificate of Consent (for signatures if you agree to take part)**

You will be given a copy of the full Informed Consent Form

PART I: Information Sheet

Introduction

I am Dr. Hang-yu Li, working for the Fourth Affiliated Hospital of China Medical University. We are doing research on different surgical techniques used to repair abnormal wall defects. I am going to give you information and invite you to be part of this research. You do not have to decide today whether or not you will participate in the research. Before you decide, you can talk to anyone you feel comfortable with about the research.

There may be some words that you do not understand. Please ask me to stop as we go through the information and I will take time to explain. If you have questions later, you can ask them of me, the study doctor or the staff.

Purpose of the research

There are few reports on the effects of different inguinal hernia repair approaches on postoperative complications, particularly regarding severe postoperative complications, in patients from northern China. The incidence of severe complications after inguinal hernia repair is relatively low, but this surgery can lead to physical impairment or organ dysfunction that greatly decreases patient quality of life and places a heavy burden on the patient's family and society. This study will compare the effectiveness of inguinal hernia repair via the anterior transversalis fascia approach versus the preperitoneal approach in patients from northern China in terms of postoperative complications, quality of life, and cost-effectiveness. This study aims to determine the optimal method for inguinal hernia repair that is suitable for the anatomic features of the inguinal region for local patients.

Type of Research Intervention

Briefly state the type of intervention that will be undertaken. This will be expanded upon in the procedures section but it may be helpful and less confusing to the participant if they

For peer review only - http://bmjopen.bmj.com/site/about/guidelines.xhtml

know from the very beginning whether, for example, the research involves a vaccine, an interview, a biopsy or a series of finger pricks.
You will be undergone inguinal hernia repair via the anterior transversalis fascia approach or the preperitoneal approach. Also you will be followed-up at the following time-points: at baseline (at admission), pre-discharge, and at 1 week, 1 month, 3 months (hospital visit or telephone follow-up), 1 year (telephone follow-up), and 2 years after surgery (telephone follow-up).

Participant selection
We are inviting all male adults with inguinal hernia who attend our hospital to participate in the research.

- ***Example of question to elucidate understanding:*** *Do you know why we are asking you to take part in this study? Do you know what the study is about?*

Voluntary Participation
Your participation in this research is entirely voluntary. It is your choice whether to participate or not. Whether you choose to participate or not, all the services you receive at this hospital will continue and nothing will change. If you choose not to participate in this research project, you will offered the treatment that is routinely offered in this hospital for inguinal hernia repair, and we will tell you more about it later. You may change your mind later and stop participating even if you agreed earlier.

- ***Examples of question to elucidate understanding:*** *If you decide not to take part in this research study, do you know what your options are? Do you know that you do not have to take part in this research study, if you do not wish to? Do you have any questions?*

Include the following section only if the protocol is for a clinical trial:

Information on the different approaches for inguinal hernia repair
1) What is the anterior transversalis fascia repair?
An oblique skin incision will be made parallel to the inguinal groove from the inner to the outer inguinal ring. Subcutaneous tissue will be dissected until the external oblique aponeurosis is reached. Hemostasis will be achieved by electric coagulation. A4 – 6 cm long skin incision will be made on the external oblique aponeurosis, starting from the pubic tubercle, to fully expose the pubic tubercle and the inner ring.
The lower part of the external oblique aponeurosis will be separated from the spermatic cord. The upper part of the external oblique aponeurosis will then be separated from the internal oblique aponeurosis and dissociated to a point 3 cm above the inguinal canal wall. The overlying spermatic cord and cremaster muscle will be lifted up and disassociated about 2 cm above the pubic tubercle. The ilioinguinal nerve, external spermatic vessels, and genital nerves concomitant with the spermatic cord will be protected while the spermatic cord is lifted.
The inguinal canal will then be dissected and the herniasac will be dissociated from the surrounding tissue. The cremaster muscle fibers will be separated longitudinally and the hernia sac will be separated from the spermatic cord. The hernia sac will be dissociated until the neck of the hernia sac is reached. All abdominal contents in the hernia sac will be returned to the abdominal cavity. In cases with a small hernia sac, the sac will also be returned to the anterior peritoneal cavity to minimize postoperative pain. In cases with a large hernia sac, the distal end will be transected and opened, and the proximal part will be ligated at a high position.
A patch designed with an arc-shaped head and a swallow tail-like end that comprises two pieces (a wide top piece and a narrow bottom piece) will be sutured and fixed with non-absorbable

For peer review only - http://bmjopen.bmj.com/site/about/guidelines.xhtml

polypropylene sutures. The inner side of the patch will be sutured to the pubic tubercle and the lateral boundary of the rectus abdominis muscle. The upper border of the patch will be sutured to the conjoined tendon and its border with the inguinal ligament and iliopubic tract. The wound will then be closed using layered sutures according to anatomic position.

2) What is the preperitoneal space repair?

The skin will be cut and subcutaneous tissue will be dissected. The abdominal external oblique aponeurosis will be dissected along the fibers. The abdominal internal oblique aponeurosis and transversus abdominis muscle will be bluntly separated to expose the transverse fascia. Dissociation of the hernia sac will be done as described above in the anterior transversalis fascia repair.

Transabdominal preperitoneal or totally extraperitoneal laparoscopic techniques were used to dissociate preperitoneal space, strip spermatic cord peritoneum, anatomize and expose the pubic pore. A polypropylene patch will be placed in the prevesical space and Bogros' space to achieve repair of the pubic pore-containing area.

3) Why we compare these two approaches?

Many surgical repair methods involving patches (of varying types and materials) are available for inguinal hernia repair. At present, there is a scarcity of clinical evidence from large-sample, randomized, controlled trials investigating the effectiveness of inguinal hernia repair via the anterior transversalis fascia approach versus the preperitoneal approach. Our outcomes will provide trial-based evidence for selection of rational therapeutic regimen in the clinic.

4) What are the side-effects of the repairs?

There are few reports on the effects of different inguinal hernia repair approaches on postoperative complications, particularly regarding severe postoperative complications, in patients in northern China. The incidence of severe complications after inguinal hernia repair is relatively low, but this surgery can lead to physical impairment or organ dysfunction that greatly decreases patient quality of life and places a heavy burden on the patient's family and society.

Procedures and Protocol

You will receive the treatment of inguinal hernia repair according to national guidelines, i.e. Guidelines for the Diagnosis and Treatment of Inguinal Hernia in Adults (2014 Edition) formulated by Chinese national society and the Adult Inguinal Hernia Treatment Guidelines (Updated Edition in 2014) developed by the European Hernia Society. This means that you will be treated either by the anterior transversalis fascia approach or the preperitoneal approach during the surgery.

In strict accordance with the Consolidated Standards of Reporting Trials (CONSORT) standards, the baseline data (preoperative data), therapeutic regimen, therapeutic outcome, and medical costs during hospitalization of patients with inguinal hernia will be recorded. Patient data will be collected using an electronic data capture system (EDC).

Description of the Process

During the research, you make seven visits to the hospital.

Visit 1 (at admission, day 0):: you will be requested to

- Sign informed consent
- Recheck inclusion/exclusion criteria
- Demographic data (sex, age, body height, body mass index) and medical insurance type
- Type of hernia (indirect or direct)
- Inguinal hernia classification as type I, II, III, or IV according to the adult inguinal hernia and femoral hernia classification (revised in 2003) developed by the Hernia and Abdominal Wall Surgery Group, Society of Surgery, Chinese Medical Association [12]

For peer review only - http://bmjopen.bmj.com/site/about/guidelines.xhtml

- ASA classification
- History of diseases and risk factors* (diabetes mellitus, cardiovascular disease, lung disease, peripheral vascular disease, dementia, hypertension, smoking history and alcohol use history) (*optional evaluation items)
- Disease onset and admission (interval from first appearance of the lump, main symptoms)
- Imaging examination (ultrasound, CT)
- Laboratory examination (routine blood testing, coagulation testing, testing of blood glucose, lipids and electrolytes, and hepatic and renal function)
- Vital signs (body temperature, pulse, respiration rate, blood pressure)
- VAS pain score
- Electrocardiography
- Concomitant treatment

Visit 2 (Pre-discharge): you will be tested

- Vital signs (body temperature, pulse, respiration rate, blood pressure)
- VAS pain score
- Laboratory examination (routine blood testing, coagulation testing, testing of blood glucose, lipids and electrolytes, and hepatic and renal function)
- Treatment regimen (inguinal hernia-specific treatment)

Surgical approach and patches (type and fixation method); anesthesia method; drug name (generic preferred), dosage, route, start date, length of drug use will be recorded.

- Concomitant treatment: Inguinal hernia-specific treatment, maintenance therapy, and drugs used for concomitant treatment.

Visit 3 (1 week after surgery via clinic visit or telephone follow-up): you will be tested

- VAS pain score
- 36-Item Short Form Health Survey (SF-36) score
- Hernia recurrence
- Presence or absence of hematoma and/or seroma
- Wound infection

Visit 4 (1 month after surgery via clinic visit or telephone follow-up): you will be tested

- VAS pain score
- SF-36 score
- Presence or absence of hematoma and/or seroma
- Wound infection

Visit 5 (3 months after surgery via clinic visit or telephone follow-up): you will be tested

- VAS pain score
- SF-36 score
- Hernia recurrence
- Presence or absence of hematoma and/or seroma
- Wound infection

For peer review only - http://bmjopen.bmj.com/site/about/guidelines.xhtml

Visit 6 (1 year after surgery via telephone follow-up): you will be tested

- VAS pain score
- SF-36 score
- Hernia recurrence
- Presence or absence of hematoma and/or seroma
- Wound infection

Visit 7 (2 years after surgery via telephone follow-up): you will be tested

- VAS pain score
- SF-36 score
- Hernia recurrence
- Presence or absence of hematoma and/or seroma
- Wound infection

Duration

In total, you will be asked to come 7 times to the hospital in 2 years. At the end of 2 years, the research will be finished.

➢ ***Examples of question to elucidate understanding:*** *Can you tell me if you remember the number of times that we are asking you to come to the hospital to complete the treatment? The research project? How many injections will you be given? How many tablets? How much blood will be taken from your veins, using a syringe and needle? Over how many weeks? Etc. Do you have any other questions? Do you want me to go through the procedures again?*

Side Effects

The incidence of severe complications after inguinal hernia repair is relatively low, any adverse medical events occurring after herniorrhaphy in patients or clinical subjects. This surgery could lead to physical impairment or organ dysfunction that decreases patient quality of life.

Serious adverse postoperative medical events involving one or more of the following criteria:

- Death, irrespective of the cause
- Life-threatening event
- Severe or permanent disability or organ dysfunction
- Hemorrhage
- Malformation, birth defect
- Hospitalization or extended hospital stay
- Re-admission to hospital
- Recurrence (symptomatic) of inguinal hernia

Risks

History of diabetes mellitus, cardiovascular disease, lung disease, peripheral vascular disease, dementia, hypertension, smoking history and alcohol use history could cause possible risks, including wound infection, rates of hematoma, seroma, hernia recurrence, intestinal obstruction, intestinal fistula, entocele, mesenteric thrombosis, femoral vein thrombosis, ischemic orchitis, painful ejaculation, varicocele, scrotal edema and etc.

While the possibility of this happening is very low, you should still be aware of the possibility. We will try to decrease the chances of this event occurring, but if something unexpected happens

For peer review only - http://bmjopen.bmj.com/site/about/guidelines.xhtml

- ***<u>Examples of question to elucidate understanding:</u>*** *Do you understand that these risks can happen whether or not you are in the research study? Etc. Do you have any other questions?*

Benefits
There may not be any benefit for you but your participation is likely to help us find the answer to the research question.

Reimbursements
We will waive the medical cost associated during your follow-up visit. You will not be given any other money or gifts to take part in this research.

- ***<u>Examples of question to elucidate understanding:</u>*** *Can you tell me if you have understood correctly the benefits that you will have if you take part in the study? Do you have any other questions?*

Confidentiality
We will not be sharing the identity of those participating in the research. The information that we collect from this research project will be kept confidential. Information about you that will be collected during the research will be put away and no-one but the researchers will be able to see it. Any information about you will have a number on it instead of your name. Only the researchers will know what your number is and we will lock that information up in the computer. It will not be shared with or given to anyone except your clinician.

- ***<u>Example of question to elucidate understanding:</u>*** *Did you understand the procedures that we will be using to make sure that any information that we as researchers collect about you will remain confidential? Do you have any questions about them?*

Sharing the Results
The results of this study will be disseminated via peer-reviewed publications and conference presentations. No data are available at the moment. Confidential information will not be shared.

Right to Refuse or Withdraw
You do not have to take part in this research if you do not wish to do so and refusing to participate will not affect your treatment at this hospital in any way. You will still have all the benefits that you would otherwise have at this hospital. You may stop participating in the research at any time that you wish without losing any of your rights as a patient here. Your treatment at this hospital will not be affected in any way.

Alternatives to Participating
If you do not wish to take part in the research, you will be provided with the anterior transversalis fascia repair at the hospital.

Who to Contact
If you have any questions you may ask them now or later, even after the study has started. If you wish to ask questions later, you may contact any of the following:
Name: Dr. Yang
Address: 4# Chong-shan East Road, Shenyang
Telephone number: (024)62255001

For peer review only

For peer review only - http://bmjopen.bmj.com/site/about/guidelines.xhtml

E-mail: 529687607@qq.com

This proposal has been reviewed and approved by Ethics Committee of the Fourth Affiliated Hospital of China Medical University, which is a committee whose task it is to make sure that research participants are protected from harm. If you wish to find about more about the IRB, contact [Dr. Yuan-zhe Jin, 4# Chong-shan East Road, Shenyang, 024-62043027.]).

- ***Example of question to elucidate understanding:*** *Do you know that you do not have to take part in this study if you do not wish to? You can say No if you wish to? Do you know that you can ask me questions later, if you wish to? Do you know that I have given the contact details of the person who can give you more information about the study?*

You can ask me any more questions about any part of the research study, if you wish to. Do you have any questions?

PART II: Certificate of Consent

I have read the foregoing information, or it has been read to me. I have had the opportunity to ask questions about it and any questions that I have asked have been answered to my satisfaction. I consent voluntarily to participate as a participant in this research.

Print Name of Participant__________________

Signature of Participant ___________________

Date ___________________________
Day/month/year

If illiterate

A literate witness must sign (if possible, this person should be selected by the participant and should have no connection to the research team). Participants who are illiterate should include their thumb-print as well.

I have witnessed the accurate reading of the consent form to the potential participant, and the individual has had the opportunity to ask questions. I confirm that the individual has given consent freely.

Print name of witness_____________________ **OR** **Thumb print of participant**

Signature of witness ______________________

Date _________________________
Day/month/year

Statement by the researcher/person taking consent

I have accurately read out the information sheet to the potential participant, and to the best of my ability made sure that the participant understands that the inguinal hernia repair will be performed and a few follow-up visits requested to finish the study.

For peer review only - http://bmjopen.bmj.com/site/about/guidelines.xhtml

I confirm that the participant was given an opportunity to ask questions about the study, and all the questions asked by the participant have been answered correctly and to the best of my ability. I confirm that the individual has not been coerced into giving consent, and the consent has been given freely and voluntarily.

A copy of this ICF has been provided to the participant.

Print Name of Researcher/person taking the consent________________________

Signature of Researcher /person taking the consent__________________________

Date ___________________________
Day/month/year

For peer review only

For peer review only - http://bmjopen.bmj.com/site/about/guidelines.xhtml

Additional file 4: List of ethical approval documents

Center	Name of ethical bodies	Ethical approval reference number
Department of General Surgery, the Fourth Affiliated Hospital of China Medical University	The Ethics Committee of the Fourth Affiliated Hospital of China Medical University	2015-027
Department of General Surgery, Branch 3, First Hospital of Dalian Medical University	The Ethics Committee of First Hospital of Dalian Medical University	2016-034
Department of General Surgery, the 202 Hospital of Chinese PLA	The Ethics Committee of the 202 Hospital of Chinese PLA	2015-033
Ward of Hernia, Department of General and Gastrointestinal Surgery, First Affiliated Hospital of Liaoning Medical University	The Ethics Committee of First Affiliated Hospital of Liaoning Medical University	2016-022
Department of General Surgery, General Hospital of Shenyang Military	The Ethics Committee of General Hospital of Shenyang Military	2015-015
Second Department of General Surgery, General Hospital of	The Ethics Committee of General Hospital of Benxin Iron and Steel	2015-011

For peer review only

Benxin Iron and Steel Co., Ltd.	Co., Ltd.	
First Department of General Surgery, Affiliated Central Hospital of Shenyang Medical University	The Ethics Committee of Affiliated Central Hospital of Shenyang Medical University	2015-022
Department of General Surgery, First Hospital of Dandong	The Ethics Committee of First Hospital of Dandong	2016-016
Shengjing Hospital of China Medical University	The Ethics Committee of Shengjing Hospital of China Medical University	2016-010

For peer review only

BMJ Open

Anterior transversalis fascia approach versus preperitoneal space approach for inguinal hernia repair in residents in Northern China: study protocol for a prospective, multi-center, randomized, controlled trial

Journal:	*BMJ Open*
Manuscript ID	bmjopen-2017-016481.R1
Article Type:	Protocol
Date Submitted by the Author:	07-Jun-2017

Complete List of Authors:	Fan, Qing; The Fourth Affiliated Hospital of China Medical University<br>Zhang, Dewei; The Fourth Affiliated Hospital of China Medical University<br>Yang, Daye; The Fourth Affiliated Hospital of China Medical University<br>Li, Hongwu; The Fourth Affiliated Hospital of China Medical University<br>Wei , Shibo; The Fourth Affiliated Hospital of China Medical University<br>Yang, Liang ; The Fourth Affiliated Hospital of China Medical University<br>Yang, Fuquan ; Shengjing Hospital of China Medical University<br>Zhang, Shaojun ; Fengtian Hospital of Shenyang Medical College<br>Wu, Yaoqiang ; The First Hospital of Dandong City<br>An, Weide ; The First Affiliated Hospital of Dalian Medical University<br>Dai, Zhongshu ; General Hospital of Benxi Steel and Iron (Group) Co., LTD, Fifth Clinical College of China Medical University<br>Jiang, Huiyong ; General Hospital of Shenyang Military Area<br>Wang, Furong ; The 202nd Hospital of PLA<br>Qiao, Shifeng ; The First Affiliated Hospital of Liaoning Medical University<br>Li, Hangyu; The Fourth Affiliated Hospital of China Medical University

**Primary Subject Heading**:	Surgery
Secondary Subject Heading:	Renal medicine
Keywords:	inguinal hernia, anterior transversalis fascia repair, preperitoneal repair, quality of life, cost-utility analysis, randomized

SCHOLARONE™
Manuscripts

Anterior transversalis fascia approach versus preperitoneal space approach for inguinal hernia repair in residents in Northern China: study protocol for a prospective, multi-center, randomized, controlled trial

Qing Fan[1], **De-wei Zhang**[1], **Da-ye Yang**[1], **Hong-wu Li**[1], **Shi-bo Wei**[1], **Liang Yang**[1], **Fu-quan Yang**[2], **Shao-jun Zhang**[3], **Yao-qiang Wu**[4], **Wei-de An**[5], **Zhong-shu Dai**[6], **Hui-yong Jiang**[7], **Fu-rong Wang**[8], **Shi-feng Qiao**[9], **Hang-yu Li**[1*]

Qing Fan The Fourth Affiliated Hospital of China Medical University, Shenyang, Liaoning Province, China Email: 18900913745@163.com

De-wei Zhang The Fourth Affiliated Hospital of China Medical University, Shenyang, Liaoning Province, China Email: syzhangdewei@sohu.com

Da-ye Yang The Fourth Affiliated Hospital of China Medical University, Shenyang, Liaoning Province, China Email:yangdaye73@126.com

Hong-wu Li The Fourth Affiliated Hospital of China Medical University, Shenyang, Liaoning Province, China Email: 13998201180@163.com

Shi-bo Wei The Fourth Affiliated Hospital of China Medical University, Shenyang, Liaoning Province, China Email: 13898111626@163.com

Liang Yang The Fourth Affiliated Hospital of China Medical University, Shenyang, Liaoning Province, China Email: 529687607@qq.com

For peer review only - http://bmjopen.bmj.com/site/about/guidelines.xhtml

Fu-quan Yang Shengjing Hospital of China Medical University, Shenyang, Liaoning Province, China Email: yangfq@sj-hospital.org

Shao-jun Zhang Fengtian Hospital of Shenyang Medical College, Shenyang, Liaoning Province, China Email: shaojunzhang@163.com

Yao-qiang Wu The First Hospital of Dandong City, Dandong, Liaoning Province, China Email: wuyaoqiang@sina.com

Wei-de An The First Affiliated Hospital of Dalian Medical University, Dalian, Liaoning Province, China Email: anweide@sina.com

Zhong-shu Dai General Hospital of Benxi Steel and Iron (Group) Co., LTD, Fifth Clinical College of China Medical University, Benxi, Liaoning Province, China Email: dzs20092009@163.com

Hui-yong Jiang General Hospital of Shenyang Military Area, Shenyang, Liaoning Province, China Email: huiyongj@126.com

Fu-rong Wang The 202nd Hospital of PLA, Shenyang, Liaoning Province, China Email: furong_jiyu@126.com

Shi-feng Qiao The First Affiliated Hospital of Liaoning Medical University, Jinzhou, Liaoning Province, China Email: shifengqiao2013@163.com

Hang-yu Li[*] The Fourth Affiliated Hospital of China Medical University, Shenyang, Liaoning Province, China Email: li_hangyu@126.com

For peer review only - http://bmjopen.bmj.com/site/about/guidelines.xhtml

*Correspondence to: Hang-yu Li, li_hangyu@126.com

For peer review only

For peer review only - http://bmjopen.bmj.com/site/about/guidelines.xhtml

Abstract

Introduction: Many surgical techniques have been used to repair abdominal wall defects in the inguinal region based on the anatomic characteristics of this region, and can be categorized as 'tension' repair or 'tension-free' repair. Tension-free repair is the preferred technique for inguinal hernia repair. Tension-free repair of inguinal hernia can be performed through either the anterior transversalis fascia approach or the preperitoneal space approach. There are few large-sample, randomized, controlled trials investigating the curative effects of the anterior transversalis fascia approach versus the preperitoneal space approach for inguinal hernia repair in patients in northern China.

Methods and analysis: This will be a prospective, large-sample, multicenter, randomized, controlled trial. Registration date is December 1, 2016. Actual Study Start Date is February 6, 2017. Estimated Study Completion Date is June 2020. A cohort of over 720 patients with inguinal hernias will be recruited from 9 institutions in Liaoning Province, China. Patient randomization will be stratified by center to undergo inguinal hernia repair via the anterior transversalis fascia approach or the preperitoneal approach. Primary and secondary outcome assessments will be performed at baseline (prior to surgery), pre-discharge, and at postoperative 1 week, 1 month, 3 months, 1 year and 2 years. The primary outcome is the incidence of postoperative chronic

For peer review only - http://bmjopen.bmj.com/site/about/guidelines.xhtml

inguinal pain. The secondary outcome is postoperative complications (including rates of wound infection, hematoma, seroma, and hernia recurrence).

Ethics and dissemination: This trial will be conducted in accordance with the Declaration of Helsinki and supervised by the institutional review board of the Fourth Affiliated Hospital of China Medical University (approval number 2015-027). All patients will receive information about the trial in verbal and written forms and will give informed consent before enrollment. The results will be published in peer-reviewed journals or disseminated through conference presentations.

Trial registration number NCT02984917; Pre-results.

Keywords: inguinal hernia; anterior transversalis fascia repair; preperitoneal repair; quality of life; cost-utility analysis; randomized controlled trial

Article summary

Strengths and limitations of this study

● Based on study results, we aim to effectively reduce physical and psychological pain, ensure high quality medical care (including safety), and achieve the best rehabilitation in the treatment of inguinal hernia.

● We aim to determine how to reduce medical resources (including shortening treatment time and reducing labor service strength) and medical costs, and improve the

For peer review only - http://bmjopen.bmj.com/site/about/guidelines.xhtml

efficiency of medical work and other issues.

● This study will provide important clinical guidance as to the method of inguinal hernia repair that is most suitable for the anatomic characteristics of patients in northern China and adaptive to the regional economic situation.

● This is a multi-center (over nine research centers), large-sample study; therefore, study conduction in each research center based on the establishment of strict regulations is key to accurate results.

For peer review only

For peer review only - http://bmjopen.bmj.com/site/about/guidelines.xhtml

BACKGROUND

History and current related studies

Inguinal hernia is a common surgical disease that manifests as protrusion of abdominal cavity contents through the inguinal canal because of an abdominal wall defect. It is more common in males than in females, with an overall incidence of 5–10%[1]. Methods for surgical repair of abdominal wall defects in the inguinal region are classified as either ‘tension’ repairs or ‘tension-free’ repairs. Herniorrhaphy through repair of the posterior wall of the inguinal canal was first described by Bassini in 1887, and is regarded as a classic surgical method[2].

As understanding of the anatomic location and pathophysical characteristics of inguinal hernia developed, the American surgeon Lichtenstein proposed a new concept of tension-free herniorrhaphy[3]. This technique was quickly adopted worldwide because of its advantages including minimal invasion, technical ease, effectiveness, low complication rate, low recurrence rate, and allowance of resumption of unrestricted physical activity. The most common technique is open tension-free herniorrhaphy.

Tension-free herniorrhaphy methods include anterior transversalis fascia repair, preperitoneal repair, abdominal cavity patch repair and combined repair approaches[4-6]. Lichtenstein herniorrhaphy is the representative technique of anterior transversalis fascia repair. Preperitoneal repair techniques include transabdominal preperitoneal,

For peer review only - http://bmjopen.bmj.com/site/about/guidelines.xhtml

total extraperitoneal, and Kugel repair techniques. The combined repair approaches refer to tension-free herniorrhaphy using a modified Kugel patch and the Ultrapro hernia system[7-8].

Many surgical repair methods involving patches (of varying types and materials) are available for inguinal hernia repair. Zhu et al.[9] performed a Meta analysis regarding open extraperitoneal approach and extraperitoneal laparoscopic hernioplasty for inguinal hernia repair. They found that these two approaches exhibited basically similar clinical outcomes. Patients receiving extraperitoneal laparoscopic hernioplasty needed shorter hospital stays and exhibited lower incidence of postoperative complications. Patients receiving open extraperitoneal approach exhibited lower incidence of peritoneal tears. Pisanu et al.[10] analyzed the clinical efficacy of laparoscopic and Lichtenstein techniques in recurrent inguinal hernia repair. They found that laparoscopicy showed lower indicience of chronic inguinal pain and an earlier return to normal daily activities but greatly longer operative time. There are many randomized controlled trials[11-15] on the clinical efficacy of inguinal hernia repair approaches (Table 1), but little is reported on anterior transversalis fascia approach and preperitoneal space approach for inguinal hernia repair in residents in Northern China.

Table 1 Randomozed controlled trials (RCTs) regarding inguinal hernia repair approaches

For peer review only - http://bmjopen.bmj.com/site/about/guidelines.xhtml

Study	Design	Subjects	Disease	Follow-up time	Outcome measures	Conclusion
Akhtar et al. [11]	RCT	TAPP (n = 30) Lichtenstein (n = 50)	Unilateral inguinal hernia	6 months	Average operation, pain score, analgesics, admission days, days required to return to work	Laparoscopic hernia surgery is better than Lichtenstein repair in terms of postoperative pain, hospital stay and return to daily activity.
Sarhan et al.[12]	RCT	A total of 200 patients scheduled for unilateral inguinal hernia repair were randomly divided into two groups to undergo either laparoscopic TAPP (group A) or open modified Kugel procedure	Unilateral inguinal hernia	32 months	Recurrence and short-term and long-term complications	Both open modified Kugel and laparoscopic TAPP preperitoneal repair techniques for inguinal hernia are safe and effective with low recurrence rates. Laparoscopic approach has better outcome in terms of chronic pain, short operative time, and short duration of hospital stays.
Kargar et al.[13]	RCT	TAPP (n = 60) Lichtenstein (n = 60)	Inguinal hernia	Follow-up occurred within 6 weeks.	Pain score (VAS), hematoma/seroma, urinary retention, wound infection, hospital stay	The laparoscopic TAPP repair is safer and less complicated approach for inguinal hernia repair. The two main short-term advantages of the laparoscopic TAPP repair with the tension free Lichtenstein repair were less postoperative pain and earlier return to the normal life activities. No difference was seen in overall complications.
Salma et al.[14]	RCT	TAPP (n = 30) Lichtenstein (n = 30)	Direct inguinal hernia	Post-operative pain intensity assessed by VAS and hospital stay measured in hours.	Hospital stay, immediate post-operative pain	There is less postoperative pain after laparoscopic repair but hospital stay is same in both the procedures but laparoscopic procedure does increase the cost.
Bahram[15]	RCT	TAPP (n = 150) Lichtenstein (n = 150)	Inguinal hernia	Three hundred patients with inguinal hernia were enrolled in this study, divided into two equal groups: Group	Operative time, intra-operative visceral injury, ileus, hospital stay or wound complications, post-operative pain, groin	TAPP technique is an excellent approach for treatment of inguinal hernia in comparison to LR either unilateral or bilateral, primary or

For peer review only - http://bmjopen.bmj.com/site/about/guidelines.xhtml

				managed by trans-abdominal pre-peritoneal laparoscopic repair (TAPP) and Group II managed by open lichtenstein repair.	hypothesia, return to activities, recurrence	recurrent inguinal hernia with low morbidity and recurrence comparable to that of lichtenstein repair with advantages of less post-operative pain and early return to activities.

TAPP: transabdominal preperitoneal.

There are few reports on the effects of different inguinal hernia repair approaches on postoperative complications, particularly regarding severe postoperative complications, in patients in northern China. The incidence of severe complications after inguinal hernia repair is relatively low, but this surgery can lead to physical impairment or organ dysfunction that greatly decreases patient quality of life and places a heavy burden on the patient's family and society. At present, there is a scarcity of clinical evidence from large-sample, randomized, controlled trials investigating the effectiveness of inguinal hernia repair via the anterior transversalis fascia approach versus the preperitoneal approach.

Main objectives

In this study, we will investigate the advantages and disadvantages of the anterior transversalis fascia approach versus the preperitoneal approach for inguinal hernia repair in residents from northern China regarding common postoperative complications

For peer review only - http://bmjopen.bmj.com/site/about/guidelines.xhtml

(including acute and chronic pain, wound infection, rates of wound infection, hematoma, seroma, and hernia recurrence) and severe postoperative complications. These outcomes will provide trial-based evidence for selection of rational therapeutic regimen in the clinic.

Distinguishing features from related studies

(1) This study will use center-based stratification to investigate the effects of anterior transversalis fascia approach versus preperitoneal space approach for inguinal hernia repair on postoperative chronic inguigal pain and other common complications. This study will determine the optimal surgical hernia repair approach that is suitable for the anatomic characteristics of the inguinal region of residents in northern China and corresponds to the regional economic conditions. (2) Cost-utility analysis will be analyzed using center-based stratification. (3) To analyze the effects of different surgical repair approaches (involving various patch types and materials) on postoperative quality of life.

Methods/Design

Study design

This is a prospective, large-sample, multi-center, randomized, controlled trial that will

For peer review only - http://bmjopen.bmj.com/site/about/guidelines.xhtml

include a cohort of over 720 patients with inguinal hernia. In strict accordance with the Consolidated Standards of Reporting Trials (CONSORT) standards[16], the baseline data (preoperative data), therapeutic regimen, therapeutic outcome, and medical costs during hospitalization of patients with inguinal hernia will be recorded. Patient data will be collected using an electronic data capture system (EDC).

According to recommendations for treatment and follow-up of inguinal hernia repair in adults in the Guidelines for the Diagnosis and Treatment of Inguinal Hernia in Adults (2014 Edition) formulated by Chinese scholars[17] and the Adult Inguinal Hernia Treatment Guidelines (Updated Edition in 2014) developed by the European Hernia Society[18], patients who undergo herniorrhaphy will be followed-up at seven time-points: at baseline (at admission, visit 1), pre-discharge, and at 1 week (visit 2), 1 month (visit 3), 3 months (visit 4, clinic visit or telephone follow-up), 1 year (visit 5, telephone follow-up), and 2 years after surgery (visit 6, telephone follow-up). The flow chart of the study protocol is shown in Figure 1. Prior to surgery: patients will be re-screened against inclusion and exclusion criteria. Signed informed consent will be obtained. Patient's demographic data, history of disease and medication, and admission condition and vital signs will be recorded. Clinical examination data will be collected from each center, including history of disease, physical examination, laboratory testing results, imaging findings, preoperative VAS pain score, intraoperative findings, and

For peer review only - http://bmjopen.bmj.com/site/about/guidelines.xhtml

details of occurrence and management methods of intraoperative injury to the intestinal tract and bladder, spermatic cord, and vascular system.

· Pre-discharge, and 1 week, 1 and 3 months, 1 and 2 years after surgery: pain, wound infection, hematoma and seroma in the inguinal region, and hernia recurrence will be recorded. Medical costs during hospitalization and patient quality of life after discharge will be also recorded. The flow chart of study protocol is shown in Figure 1.

Patients

Patients with inguinal hernia will be recruited from nine trial centers in northern China: the Department of General Surgery, the Fourth Affiliated Hospital of China Medical University; Department of General Surgery, Branch 3, First Hospital of Dalian Medical University; Department of General Surgery, the 202 Hospital of Chinese PLA; Ward of Hernia, Department of General and Gastrointestinal Surgery, First Affiliated Hospital of Liaoning Medical University; Department of General Surgery, General Hospital of Shenyang Military; Second Department of General Surgery, General Hospital of Benxin Iron and Steel Co., Ltd.; First Department of General Surgery, Affiliated Central Hospital of Shenyang Medical University; Department of General Surgery, First Hospital of Dandong; Shengjing Hospital of China Medical University.

For peer review only - http://bmjopen.bmj.com/site/about/guidelines.xhtml

Inclusion criteria

Male patients presenting with all of the following conditions will be considered for study admission:

· Diagnosed with primary unilateral inguinal hernia

· Aged 18–80 years

· American Society of Anesthesiologists (ASA) classification I–II

· Provision of informed consent

Exclusion criteria

Patients with any one or more of the following will be excluded from this study:

· Severe organ dysfunction or inability to tolerate surgery

· Hernia recurrence

· Giant hernia (inner size of the hernia > 4 cm)

· Scrotal hernia

· Incarcerated inguinal hernia

· Inability to complete follow-up or questionnaire because of mental disorder or other reasons

· History of preperitoneal surgery, such as radical prostatectomy

For peer review only - http://bmjopen.bmj.com/site/about/guidelines.xhtml

Randomization and blinding

This study is a multi-center trial, so stratified block randomization will be performed in each center. A randomization sequence table will be generated by a statician who will not be involved in the trial using Statistical Analysis System (SAS 9.1). The serial numbers assigned to each patient will be preserved in opaque sealed envelopes. The sealed envelopes will be subsequently given to the trial center. All patients will not know the surgical regimen until after the surgery. The surgeons will not be blinded to the surgical regimen. Outcome assessors will be blinded to the surgical records in the electronic case report form (eCRF).

Interventions

Based on recommendations for treatment and follow-up of inguinal hernias in adults made by the Chinese Medical Association and Chinese Medical Doctor Association[17] and the European Hernia Society[18].

Anterior transversalis fascia repair

An oblique skin incision will be made parallel to the inguinal groove from the inner to the outer inguinal ring. Subcutaneous tissue will be dissected until the external oblique aponeurosis is reached. Hemostasis will be achieved by electric coagulation. A4–6 cm

For peer review only - http://bmjopen.bmj.com/site/about/guidelines.xhtml

long skin incision will be made on the external oblique aponeurosis, starting from the pubic tubercle, to fully expose the pubic tubercle and the inner ring.

The lower part of the external oblique aponeurosis will be separated from the spermatic cord. The upper part of the external oblique aponeurosis will then be separated from the internal oblique aponeurosis and dissociated to a point 3 cm above the inguinal canal wall. The overlying spermatic cord and cremaster muscle will be lifted up and disassociated about 2 cm above the pubic tubercle. The ilioinguinal nerve, external spermatic vessels, and genital nerves concomitant with the spermatic cord will be protected while the spermatic cord is lifted.

The inguinal canal will then be dissected and the herniasac will be dissociated from the surrounding tissue. The cremaster muscle fibers will be separated longitudinally and the hernia sac will be separated from the spermatic cord. The hernia sac will be dissociated until the neck of the hernia sac is reached. All abdominal contents in the hernia sac will be returned to the abdominal cavity. In cases with a small hernia sac, the sac will also be returned to the anterior peritoneal cavity to minimize postoperative pain. In cases with a large hernia sac, the distal end will be transected and opened, and the proximal part will be ligated at a high position.

A patch designed with an arc-shaped head and a swallow tail-like end that comprises two pieces (a wide top piece and a narrow bottom piece) will be sutured and fixed with

For peer review only - http://bmjopen.bmj.com/site/about/guidelines.xhtml

non-absorbable polypropylene sutures. The inner side of the patch will be sutured to the pubic tubercle and the lateral boundary of the rectus abdominis muscle. The upper border of the patch will be sutured to the conjoined tendon and its border with the inguinal ligament and iliopubic tract. The wound will then be closed using layered sutures according to anatomic position.

Preperitoneal space repair

The skin will be cut and subcutaneous tissue will be dissected. The abdominal external oblique aponeurosis will be dissected along the fibers. The abdominal internal oblique aponeurosis and transversus abdominis muscle will be bluntly separated to expose the transverse fascia. Dissociation of the hernia sac will be done as described above in the anterior transversalis fascia repair.

Transabdominal preperitoneal or totally extraperitoneal laparoscopic techniques were used to dissociate preperitoneal space, strip spermatic cord peritoneum, anatomize and expose the pubic pore. A polypropylene patch will be placed in the prevesical space and Bogros' space to achieve repair of the pubic pore-containing area.

Concomitant treatment

Any medications, with the exception of inguinal hernia-specific treatments,

For peer review only - http://bmjopen.bmj.com/site/about/guidelines.xhtml

administered during hospitalization will be recorded. Before starting the trial (i.e., at the first visit), detailed information will be recorded regarding concomitant diseases, combined medication and measures to be taken. At discharge, changes in medications and measures to be taken will be recorded. For every combination of medication and measures to be taken, a minimum of the following information will be recorded: drug name (generic preferred), dosage, start date, stop date or continuing use, and indications.

Study flowchart

Before surgery

Visit 1 (at admission, day 0)

· Sign informed consent

· Recheck inclusion/exclusion criteria

·Demographic data (sex, age, body height, body mass index) and medical insurance type

· Type of hernia (indirect or direct)

· Inguinal hernia classification as type I, II, III, or IV according to the adult inguinal hernia and femoral hernia classification (revised in 2003) developed by the Hernia and Abdominal Wall Surgery Group, Society of Surgery, Chinese Medical Association[19]

For peer review only - http://bmjopen.bmj.com/site/about/guidelines.xhtml

· ASA classification

· History of diseases and risk factors* (diabetes mellitus, cardiovascular disease, lung disease, peripheral vascular disease, dementia, hypertension, smoking history and alcohol use history) (*optional evaluation items)

·Disease onset and admission (interval from first appearance of the lump, main symptoms)

· Imaging examination (ultrasound, CT)

· Laboratory examination (routine blood testing, coagulation testing, testing of blood glucose, lipids and electrolytes, and hepatic and renal function)

· Vital signs (body temperature, pulse, respiration rate, blood pressure)

·VAS pain score

· Electrocardiography

· Concomitant treatment

The baseline information of patients with inguinal hernia included in this study is shown in Table 2.

For peer review only

Table 2 Baseline information of patients with inguinal hernia

Sex	Smoking history
Age	History of alcohol use
Body height	Disease attack and admission

For peer review only - http://bmjopen.bmj.com/site/about/guidelines.xhtml

Body mass index	Laboratory examination
Medical insurance type	Imaging examination
Type of hernia	Vital sign
Inguinal hernia classification	Visual Analogue Scale (VAS) pain score
Treatment time	Electrocardiography
American Society of Anesthesiologist Classification	Concomitant therapy
History of diseases	
Diabetes mellitus	
Cardiovascular disease	
Lung disease	
Peripheral vascular disease	
Dementia	
Hypertension	

Pre-discharge

Visit 2

· Vital signs (body temperature, pulse, respiration rate, blood pressure)

· Laboratory examination (routine blood testing, coagulation testing, testing of blood glucose, lipids and electrolytes, and hepatic and renal function)

· Treatment regimen (inguinal hernia-specific treatment)

Surgical approach and patches (type and fixation method); anesthesia method; drug name (generic preferred), dosage, route, start date, length of drug use will be recorded.

For peer review only - http://bmjopen.bmj.com/site/about/guidelines.xhtml

· Medical costs

·Concomitant treatment

Inguinal hernia-specific treatment, maintenance therapy, and drugs used for concomitant treatment.

· Medical costs because of adverse events (AE)

Follow-up

Visit 3 (1 week after surgery via clinic visit or telephone follow-up)

· 36-Item Short Form Health Survey (SF-36) score

· Medical costs

· Hernia recurrence (date and severity of recurrence).Recurrence will be diagnosed by the medical institutions, after being alerted by patients that symptoms are present.

· Presence or absence of hematoma and/or seroma

· Wound infection (date and severity of wound infection)

Visit 4 (1 month after surgery via clinic visit or telephone follow-up)

· SF-36 score

· Medical costs

· Hernia recurrence (date and severity of recurrence). Recurrence will be diagnosed by

For peer review only - http://bmjopen.bmj.com/site/about/guidelines.xhtml

the medical institutions, after being alerted by patients that symptoms are present.

· Presence or absence of hematoma and/or seroma

· Wound infection (date and severity of wound infection)

Visit 5 (3 months after surgery via clinic visit or telephone follow-up)

· SF-36 score

· Medical costs

· Hernia recurrence (date and severity of recurrence). Recurrence will be diagnosed by the medical institutions, after being alerted by patients that symptoms are present.

· Presence or absence of hematoma and/or seroma

· Wound infection (date and severity of wound infection)

Visit 6 (1 year after surgery via telephone follow-up)

· VAS pain score

· SF-36 score

· Medical costs· Hernia recurrence (date and severity of recurrence). Recurrence will be diagnosed by the medical institutions, after being alerted by patients that symptoms are present.

· Presence or absence of hematoma and/or seroma

For peer review only - http://bmjopen.bmj.com/site/about/guidelines.xhtml

· Wound infection (date and severity of wound infection)

Visit 7 (2 years after surgery via telephone follow-up)

· VAS pain score

· SF-36 score

· Medical costs

· Hernia recurrence (date and severity of recurrence). Recurrence will be diagnosed by the medical institutions, after being alerted by patients that symptoms are present.

· Presence or absence of hematoma and/or seroma

· Wound infection (date and severity of wound infection)

Outcome measures

·Primary outcome measure

The incidence of chronic inguinal pain at 1 and 2 years after surgery. To reduce the outcome bias of a single evaluation, chronic pain at 1 and 2 years after surgery will be evaluated. According to International Association for the Study of Pain, VAS pain score > 0 for over 3 successive months indicatates chronic inguinal pain[20].

·Secondary outcome measures

For peer review only - http://bmjopen.bmj.com/site/about/guidelines.xhtml

For peer review only

Postoperative complications including wound infection, rates of hematoma, seroma, hernia recurrence, intestinal obstruction, intestinal fistula, entocele, mesenteric thrombosis, femoral vein thrombosis, ischemic orchitis, painful ejaculation, varicocele and scrotal edema at postoperative 1 week, 1 and 3 months, and 1 and 2 years.

·Other outcome measures

Quality of life as evaluated by the SF-36[21]. The SF-36 is a 36-item, patient-reported survey of patient health. It consists of eight scaled scores, including vitality, physical functioning, bodily pain, general health perceptions, physical functioning, emotional functioning, social functioning, and mental health. The score of each scale is summed and then standardized according to the formula: standardized score = (actual raw score- lowest possible raw score)/possible raw score range ×100. The total SF-36 score is the standardized score based on the sum of the eight scaled scores. A higher score indicates better quality of life.

The cost-utility analysis of therapeutic regimens involving different surgical approaches will be analyzed. Medical costs consist of direct medical and non-medical costs. The direct medical costs include drug charges, inspection fees, laboratory fees, treatment fees, nursing fees, and bed charges. The direct medical costs during hospitalization will be calculated according to the hospital information system. Direct medical costs during

For peer review only - http://bmjopen.bmj.com/site/about/guidelines.xhtml

the follow-up period will be reported by patients and/or their relatives. Direct non-medical costs include payments for transportation to receive medical care, cost of nutritional supplementation, and the costs for accompanying family members during the treatment period.

All outcome evaluations will be independently performed by an experienced assessor blinded to the treatment regimens. The schedule of outcome measurement assessment is shown in Table 3.

Table 3 Timing of outcome measurement assessment

	Before surgery		During surgery		Follow up		
	Visit 1 (at admission, day 0)	Visit 2 (at discharge)	Visit 3 (1 week after surgery)	Visit 4 (1 month after surgery)	Visit 5 (3 months after surgery)	Visit 6 (1 year after surgery)	Visit 7 (3 years after surgery)
Signed informed consent	X						
Inclusion/exclusion criteria	X						
Demographic data	X						
Medical insurance type	X						

For peer review only - http://bmjopen.bmj.com/site/about/guidelines.xhtml

Delayed visit and admission	X						
Previous history of diseases	X						
Previous history of drug	X						
Risk factors	X						
Disease attack and admission [a]	X						
Type of hernia (indirect hernia, direct hernia)	X						
Vital sign[b]	X						
Laboratory examination [c]	X	X					
Imaging examination [d]	X						
Electrocardiography	X						
Treatment regimen [e]		X					
VAS score [f]						X	X
SF-36 score			X	X	X	X	X
Medical cost		X	X	X	X	X	X
Concomitant	X	X					

For peer review only - http://bmjopen.bmj.com/site/about/guidelines.xhtml

treatment							
Adverse events<br>Wound infection<br>Hematoma<br>Seroma<br>Hernia<br>recurrence		X	X	X	X	X	X

[a] indicates the interval from the first appearance of the lump or main symptoms; [b] indicates body temperature, pulse, respiration rate, and blood pressure; [c] indicates routine blood testing, coagulation testing, testing of blood glucose, lipids, and electrolytes, and hepatic and renal function; [d] indicates ultrasound, CT examination; [e] indicates inguinal hernia-specific treatment; f indicates the VAS pain score.

Adverse events and serious adverse events (SAE)

According to the study protocol and clinical judgment, AE/SAE occurring after herniorrhaphy will be reported to the Department of General Surgery, the Fourth Affiliated Hospital of China Medical University.

AE refer to any adverse medical events occurring after herniorrhaphy in patients or clinical subjects. SAE refer to any adverse postoperative medical events involving one or more of the following criteria:

○ Death, irrespective of the cause

For peer review only

For peer review only - http://bmjopen.bmj.com/site/about/guidelines.xhtml

○ Life-threatening event

○ Severe or permanent disability or organ dysfunction

○ Hemorrhage

○ Malformation, birth defect

○ Hospitalization or extended hospital stay

○ Re-admission to hospital

○ Recurrence (symptomatic) of inguinal hernia

Causal relationship between surgery and adverse events

The causal relationship between the drugs used and AE will be evaluated by the researchers as: certainly relevant, probably relevant, likely relevant, unlikely relevant and irrelevant (Table 4).

Table 4 Causal relationship between surgery and adverse events

	Certainly relevant	Probably relevant	Likely relevant	Unlikely relevant	Irrelevant
Adverse events are obviously caused by external factors	-	-	-	-	+
Adverse events are correlated with	+	+	+	-	-

For peer review only - http://bmjopen.bmj.com/site/about/guidelines.xhtml

surgical treatment at rational time					
Adverse events are correlated with patient diseases	-	-	+	+	+
Adverse events are correlated with suspected postoperative response patterns	+	+/-	+	-	-
After relief of related surgical factors, adverse events alleviate or disappear	+	+	-	-	-
After surgery-related factors worsen, adverse events recur	+	+	-	-	-

Note: To minimize the surgical risk and meet the requirements of laws and regulations, the sponsor will manage the correlations as follows: “Irrelevant” belongs to the irrelevant category, and “certainly relevant”, “probably relevant”, “likely relevant” and “unlikely relevant” belong to the relevant category.

Evaluation criteria for the severity of adverse events

For peer review only - http://bmjopen.bmj.com/site/about/guidelines.xhtml

Mild: The patient is aware of symptoms, but symptoms can be tolerated. Symptoms are causing mild discomfort, but not interfering with daily activities.

Moderate: Not affecting daily activities.

Severe: Very painful, causing significant functional impairment or loss of self-care ability, and prohibiting the patient from carrying out daily activities.

The researcher will evaluate the severity of AE according to clinical indices such as laboratory and inspection outcomes, not just based on the subject's direct feelings.

Adverse event reporting

Reporting time limit for AE will be within 24 hours of onset. In the clinical research period (from the time of signing informed consent to 1–2 years postoperatively), any AE occurring in any patient who received either surgical approach will be properly treated. According to the requirements of this study protocol and clinical evaluation, researchers will fill in the table of *Adverse Events After Herniorrhaphy* and submit this within 24 hours to the Clinical Research Center of Abdominal Wall Hernia, the Fourth Affiliated Hospital of China Medical University, China.

Serious adverse event emergency reporting

Reporting time limit for SAE will be within 24 hours of onset. Any SAE will be

For peer review only - http://bmjopen.bmj.com/site/about/guidelines.xhtml

continuously monitored and reported until it is healed, stabilizes or recovers to near baseline conditions, irrespective of whether patients have terminated or completed treatment. Any follow-up information regarding SAE will be reported within 24 hours.

Patient completion/withdrawal from clinical study

Patients for whom the whole clinical data of at least 1 year are collected will be considered as complete cases. Patients with any one or more of the following criteria will be considered withdrawn from the study: mistakenly recruited, withdrawal of informed consent, upon the request of the sponsor for safety reasons or patient conflicts, or lost to follow-up. The date and reasons for withdrawal will be recorded on the eCRF. After termination of the study, the data collected at the last visit will be evaluated, except for data from those lost to follow-up.

Statistical analysis

Statistical analysis will be performed by a statician using SPSS 19.0 software (SPSS, Chicago, IL, USA). Continous variables will be statistically described using the mean, standard deviation, median, minimum and maximum. The categorical variables will be expressed using numbers and percentages.

The analysis will be performed on the basis of the intention-to-treat principle.

For peer review only - http://bmjopen.bmj.com/site/about/guidelines.xhtml

Descriptive statistics of baseline information will be performed. The chi-squared test or Fisher's exact test will be used for analysis of categorical variables, such as the incidence of postoperative chronic pain (primary outcome measure) and the incidence of postoperative complications (secondary outcome measure) between groups. Independent sample t-test or Mann-Whitney U test will be used for comparisons of continous variables, such as SF-36 score, between groups. Cost-utility analysis will be used for economic evaluation, and sensitivity analysis of cost and utility will be performed.

Interim analysis

When an adequate number of patients are enrolled and followed-up, interim analysis will be performed. When data are included for the full analysis set and recorded in the database, the first interim analysis during the management period will be performed to check whether the core data collected are suitable for preliminary significant data analysis. According to research progression, subsequent interim analysis of all data included in the database will then be designated. After acquiring approval from the Department of General Surgery and Scientific Construction Committee, the Fourth Affiliated Hospital of China Medical University, the data collected in the Department of General Surgery, the Fourth Affiliated Hospital of China Medical University are likely to

For peer review only - http://bmjopen.bmj.com/site/about/guidelines.xhtml

be analyzed together with the data collected from the other research centers. In accordance with applicable laws and regulations, the information on the subjects in the study will be kept confidential. The data for interim analysis will be precisely described in a statistical analysis plan. The interim analysis results will be submitted to an independent Data Monitoring Committee in the form of a statistical analysis report and as slides.

Sample size

According to previous reports[22,23], we hypothesized that the incidence of chronic inguinal pain after anterior transversalis fascia repair and preperitoneal space approach was 10% and 3.4%, respectively. Taking α = 0.05 and power = 90%, the final effective sample size of n = 600 was calculated. Assuming a patient loss rate of 20%, we require 720 patients.

Ethical Approval

Before study commencement, the following files will be provided to the Independent ethics committee (IEC):

○ Final draft of study protocol (and supplements)

○ Sponsor-approved informed consent and other documents provided to the subjects

For peer review only - http://bmjopen.bmj.com/site/about/guidelines.xhtml

(such as participation card and diary card)

○ Materials assisting patients to be included

○ Materials regarding study-related injury compensations or rewards for patient participation in the study

○ Researcher résumé or equivalent (unless the IEC states that this is not needed)

○ Sponsor name, funds, potential competing interests, and information that affects patient participation in the study

○ Any other documents required by the IEC

Trials cannot be started until the IEC completely approves the study protocol, informed consent, materials assisting patients to be included, and compensation measures for the patients, and the sponsor receives a copy of the IEC approval document. The IEC approval document should include the trial title (registration number), name of the study file (including edition code) and date of approval.

During the study period, it is likely that researchers will submit the following files to the IEC for approval at appropriate time-points:

○ Supplement of study protocol

○ Informed consent forms and documents regarding rewards for patient participation in

For peer review only - http://bmjopen.bmj.com/site/about/guidelines.xhtml

the study

○ New information that is likely to negatively affect participant safety and study progression

○ Files regarding bias and alterations of the study protocol made to avoid immediate injury to patients

○ Reports regarding dead patients

○ Notification of change of project manager

○ Other requirements of the IEC

If the supplemented study protocol increases the risk to patients, the supplemented study protocol and corresponding modified informed consent form will be submitted to the IEC for consideration. The supplemented study protocol will not be performed until approval from the IEC is obtained. The major study protocol was approved by the IEC, the Fourth Affiliated Hospital of China Medical University (approval number 2015-027) on November 27, 2015.The study protocol should be reviewed by the IEC at least once every year, and the reviewed suggestion will be recorded on paper. At the end of the study, the researchers should inform the IEC of its completion.

Informed Consent to participate

For peer review only - http://bmjopen.bmj.com/site/about/guidelines.xhtml

Each patient (or his/her legal representative) will provide signed and dated informed consent before surgery after fully understanding the objective and contents of the study. The researcher or his/her authorized staff members will fully explain the objective, methods, possible benefits, potential risks and any possible discomforts of the study to the potential patients before inclusion. Participants will be informed that participation in the study is voluntary and that they can withdraw from the study at any time. The participants will know that their identifying information will be recorded for long-term follow-up, and will be read by personnel from the related institutions and the sponsor within the permit of relevant laws and regulations. The right to privacy of the participant will be protected.

Confidentiality

○ Only data required to investigate the effectiveness and safety of herniorrhaphy will be collected and analyzed.

○ Data collection and use will not be disclosed to any non-authorized persons, and will be performed in accordance with the laws and regulations regarding protection of the participant's privacy.

○ The process of data collection will be fair and lawful.

○ The purpose of data collection will be specific, identified and legitimate, and the

For peer review only - http://bmjopen.bmj.com/site/about/guidelines.xhtml

collected data will not be used for other unrelated objectives.

○ The data collected will be adequate, related and not redundant relative to the study objective.

○ The data collected will be accurate and updated when necessary.

○ Before collection of personal data, researchers will obtain participant consent, which should include lay emphasis on the transfer of data to other institutional entities and countries.

○ The participants have the right to obtain their data and can request to modify mistaken or incomplete data.

○ During the study period, participant's personal information will not be obtained or disclosed to non-authorized persons, and will not be illegally destroyed, lost or altered unexpectedly. During the entire study period, the sponsors who have the right to read the participant's personal information will keep the data confidential.

Data management

Protocol modification

Study protocol modification will be signed, dated, and published by the Department of General Surgery, the Fourth Affiliated Hospital of China Medical University. The study protocol will not be put into clinical practice until IEC approval is received, unless this is

For peer review only - http://bmjopen.bmj.com/site/about/guidelines.xhtml

necessary to avoid risk to participants, or to modify the study protocol regarding logistics and administration (for example, typographical errors and contradictions).

The study protocol should not be deviated from during clinical practice. When deviation exists, corresponding management will be performed. The causes and deviated contents will be recorded in the eCRF and original medical case notes. The study protocol deviation table and eCRF will be preserved in the research center and sponsor institute.

Participant identity registration and screening records

Participants must agree to fill in identification registration to enable individual identification of each participant. The monitor will recheck the integrity of this registration. The participant identity registration form will be confidential, and will be preserved in the research center. To ensure confidentiality, duplication of participant identity registration will be not permitted. All reports and letters relating to this study will be tagged with the relevant acronyms and serial number. The participant screening record form will be completed by doctors. The doctors will determine whether participants are eligible for admission to this study.

Electronic case report form

For peer review only - http://bmjopen.bmj.com/site/about/guidelines.xhtml

In this study, the EDC will be used for data collection and management. All data relating to this study will be recorded on the eCRF provided by the sponsor. The researchers will fill in the eCRF after each participant visit, unless some clinical results cannot be acquired immediately. This ensures that the information recorded on the eCRF reflects the participant's latest outcome. Data accuracy will be performed by the researcher. Data recording, alteration and substitution will be performed by researchers or other authorized persons. All data will be inputted into the EDC, and data queries will be made by researchers online via the EDC. The final data will not be altered, and will be password-protected.

Data quality assurance

To ensure data accuracy and reliability, eligible researchers and appropriate research centers will be selected before study commencement. The monitor in the co-sponsor research center will monitor the study progression periodically. The co-sponsor will advise the researchers how to fill in the eCRF. The monitor in the co-sponsor research center will visit the EDC to check the integrity and accuracy of the eCRF. Data recorded in the eCRF that is inconsistent with original data recorded will be altered by researchers or authorized persons.

For peer review only - http://bmjopen.bmj.com/site/about/guidelines.xhtml

Auditing

Regular on-site inspection visits will be made by the co-sponsors. The co-sponsors monitor will date the inspection on an inspection form, which will then be preserved in the research center. After study commencement, the first on-site inspection visit will be performed as soon as possible after participant recruitment. During on-site inspection, the monitor will check the consistency of data recorded in the eCRF with original data recorded in medical notes from the research center. The nature and preservation place of original data documents will be confirmed to enable clinical researchers to know the source of all original data required in the eCRF, thus the monitor of the co-sponsor can recheck these data.

If original data are electronically preserved, the monitor of the co-sponsor will discuss the recheck method with clinical researchers. The original data document will include participant identity, eligibility for inclusion, informed consent, dates of visits, execution of study protocol, curative effects, safety index, AE reporting and follow-up, medication, and date of study completion.

The monitor of the co-sponsor will discuss the detailed requirements for original data recording with clinical researchers. To recheck whether the data recorded in eCRF is consistent with original data, the monitor of the co-sponsor will be provided with the required original data. The monitor will discuss any problems found during rechecking

For peer review only - http://bmjopen.bmj.com/site/about/guidelines.xhtml

of data consistency with clinical researchers. The clinical researchers will regularly discuss the information feedback.

Study completion/termination

Study completion

When the last visit of the last participant is completed, the research center will inform the sponsor, and study completion will be designated. The sponsor will inform all research centers of the time of study completion. Further research after this time must be approved by the sponsor and can then be performed without protocol supplements.

Study termination

The sponsor will have the right to terminate the study at any research center at any time possibly because of, but not limited to, the following criteria:

○ The number of patients recruited reaches the predetermined requirements

○ Research cannot abide by the study protocol or GCP guidelines

○ Insufficient numbers of participants are recruited

Audit and inspection

A representative of the department of clinical quality assurance of the co-sponsor may

For peer review only - http://bmjopen.bmj.com/site/about/guidelines.xhtml

visit any of the research centers to determine whether the study protocol follows the laws and regulations. All study records, including original medical notes, will be disclosed to the representative. However, the privacy of the subject will be respected. The research center will be informed about this visit in advance to allow sufficient time for appropriate preparation.

Data use and publication

Any unpublished information provided by the sponsors, and all unpublished data relating to this study will be kept confidential and will be owned by the sponsor. This information or data will be not be used for other purposes unless written approval is acquired from the sponsor. The clinical researchers will be informed that study results will be used for further study. Therefore, the study results may be provided to other clinical researchers or related administrative departments. The study results will be disclosed in the form of a Clinical Study Report, including data collected from any research center involved. If clinical researchers publish the study outcomes, they will provide the original manuscript to the sponsor for online review 60 days before submission or presentation. A summary, posters or other promotional materials will be created to facilitate the review. The sponsor will discuss scientific and regulatory compliance issues with clinical researchers. The sponsor will not mandatorily require

For peer review only - http://bmjopen.bmj.com/site/about/guidelines.xhtml

the clinical researchers to modify the scientific contents, and has no right to hide information. The clinical researchers should consider the integrity of this multi-center study. The data from one research center can be published only under the following circumstances: the articles involving the outcomes from all research centers have been published; study in all research centers has been accomplished, abandoned or terminated for 12 months; the sponsor has stated that they will not publish the study outcomes from multiple research centers. Assignment of the author listings in articles related to this study will be performed based on the author's contribution guidelines, such as the guidelines of Uniform Requirements for Manuscripts Submitted to medical journals.

DISCUSSION

Significance of this study

This study will be the first large sample (720 patients), multi-center, randomized, controlled, clinical trial to investigate the effectiveness of inguinal hernia repair via the anterior transversalis fascia approach versus the preperitoneal approach in patients from northern China. In this study, postoperative complications will be used as the primary outcome measure, and patient quality of life and cost-utility analysis will be the secondary outcome measures.

For peer review only - http://bmjopen.bmj.com/site/about/guidelines.xhtml

Trial status

Recruitment of patients is ongoing at the time of submission.

Abbreviations

AE	adverse event
eCRF	electronic case report form
EDC	electronic data capture
IEC	independent ethics committee
SAE	serious adverse event
CUA	Cost utility analysis

For peer review only

For peer review only - http://bmjopen.bmj.com/site/about/guidelines.xhtml

References

1. Beddy P, Ridgway PF, Geoghegan T, *et al*. Inguinal hernia repair protects testicular function: a prospective study of open and laparoscopic herniorraphy. *J Am Coll Surg*. 2006;203:17-23.

2. Palermo M, Acquafresca PA, Bruno M, Tarsitano F. Hernioplasty with and without mesh: analysis of the immediate complications in a randomized controlled clinical trial. *Arq Bras Cir Dig*. 2015;28:157-160.

3. Chatzimavroudis G, Papaziogas B, Koutelidakis I*, et al.* Lichtenstein technique for inguinal hernia repair using polypropylene mesh fixed with sutures vs. self-fixating polypropylene mesh: a prospective randomized comparative study. *Hernia*. 2014;18:193-198.

4. Bittner R, Arregui ME, Bisgaard T, *et al*. Guidelines for laparoscopic (TAPP) and endoscopic (TEP) treatment of inguinal hernia [International Endohernia Society (IEHS)]. *Surg Endosc*. 2011;25:2773-843.

5. Carter J, Duh QY. Laparoscopic repair of inguinal hernias. *World J Surg*. 2011;35:1519-25.

6. Greater China Endo Hernia Institute; Laparoscopic and Endoscopic Group, Society of Surgery, Chinese Medical Association; Hernia and Abdominal Wall Surgery Group, Society of Surgery, Chinese Medical Association. Guidelines for

For peer review only - http://bmjopen.bmj.com/site/about/guidelines.xhtml

Standardization of Laparoscopic Surgery for Inguinal Hernia. *Chin J Hernia Abdominal Wall Surg* (Electronic). 2013;7:505-512..

7. Karateke F, Ozyazici S, Menekse E, *et al*. ULTRAPRO Hernia System versus lichtenstein repair in treatment of primary inguinal hernias: a prospective randomized controlled study. *Int Surg*. 2014;99:391-7.
8. Kugel RD. The Kugel repair for groin hernias. *Surg Clin North Am*. 2003;83:1119-39.
9. Zhu X, Cao H, Ma Y, *et al*. Totally extraperitoneal laparoscopic hernioplasty versus open extraperitoneal approach for inguinal hernia repair: a meta-analysis of outcomes of our current knowledge. *Surgeon*. 2014;12(2):94-105.
10. Pisanu A, Podda M, Saba A, Porceddu G, Uccheddu A. Meta-analysis and review of prospective randomized trials comparing laparoscopic and Lichtenstein techniques in recurrent inguinal hernia repair. *Hernia*. 2015;19:355-366.
11. Akhtar MS, Ali WM, Khan TA, *et al*. Laparoscopic transabdominal preperitoneal versus open mesh lichtenstein repair of inguinal hernia: a comparative analysis. *Int J Med Res Rev*. 2016;4:1119-1206.
12. Sarhan AE, Sherif T, El-Kenany Y. Open preperitoneal mesh repair versus laparoscopic transabdominal preperitoneal repair of groin hernia under spinal

For peer review only - http://bmjopen.bmj.com/site/about/guidelines.xhtml

anesthesia: results of a prospective randomized multicenter trial. *Egypt J Surg*. 2016;35:132-139

13. Kargar S, Shiryazdi SM, Zare M, Mirshamsi MH, Ahmadi S, Neamatzadeh H. Comparison of postoperative short-term complications after laparoscopic transabdominal preperitoneal (TAPP) versus Lichtenstein tension free inguinal hernia repair: a randomized trial study. *Minerva Chir*. 2015;70:83-89.
14. Salma U, Ahmed I, Ishtiaq S. A comparison of post operative pain and hospital stay between Lichtenstein's repair and Laparoscopic Transabdominal Preperitoneal (TAPP) repair of inguinal hernia: A randomized controlled trial. *Pak J Med Sci*. 2015;31:1062-1066.
15. Bahram MAL. Early and late outcomes of trans-abdominal pre-peritoneal and Lichtenstein repair for inguinal hernia, a comparative study. *Int Surg J*. 2017;4:459-464.
16. CONSORT Group (2008) CONSORT: Consolidated Standards of Reporting Trials.
17. Abdominal Wall Surgery Group, Society of Surgery, Chinese Medical Association. Guidelines for the Diagnosis and Treatment of Inguinal Hernia in Adults (2014 Edition).*Chin J Prac Surg*. 2014;52:484-486.
18. Miserez M, Peeters E, Aufenacker T, *et al*. Update with level 1 studies of the European Hernia Society guidelines on the treatment of inguinal hernia in adult

For peer review only - http://bmjopen.bmj.com/site/about/guidelines.xhtml

patients. Hernia. 2014;18:151-163.

19. The Chinese Medical Association Society of Surgery and Abdominal Wall Hernia Surgery Group. Adult inguinal hernia and femoral hernia surgery methods (revised). *Chin J Gen Surg*. 2004;19:126.
20. Nikkolo C, Lepner U. Chronic pain after open inguinal hernia repair. *Postgrad Med*. 2016;128:69-75.
21. Lins L, Carvalho FM. SF-36 total score as a single measure of health-related quality of life: Scoping review. *SAGE Open Med*. 2016;4:2050312116671725.
22. Yin WT，Li P，Zhao WS，Li RH. The Analysis and Influence of Anterior Preperitoneal Space on Chronic Pain after Herniorrhaphy. *J Kunming Med Univ*. 2011;(5):90-92.
23. Zhang J, Bai J, Li Q, Li ZH, Li L. Anterior versus posterior tension-free repair of adult inguinal hernia. *Shaanxi Med J*. 2011;40:331-332.

For peer review only - http://bmjopen.bmj.com/site/about/guidelines.xhtml

Authors' contributions

HYL, FQY, SJZ, YQW, WDA, ZSD, HYJ, FRW and SFQ conceived the study and participated in its design and coordination. LY drafted the manuscript. SBW and QF participated in the design of the study and performed the statistical analysis. DWZ and DYY participated in the study design and coordination and helped draft the manuscript. HWL participated in the design of the study and wrote the protocol for the analysis. All authors read, revised and approved the final manuscript.

Funding Statement

This research was funded by National Natural Science FoundationNO.81472302.

Competing interests Statement

The authors declare that they have no competing interests.

Data sharing statement

The results of this study will be disseminated via peer-reviewed publications and conference presentations. No data are available at the moment.

For peer review only

For peer review only - http://bmjopen.bmj.com/site/about/guidelines.xhtml

Figure 1 Flow chart of study protocol.

VAS: Visual Analogue Scale; SF-36: 36-Item Short Form Health Survey.

Tables

Table 1 Randomized controlled trials (RCTs) regarding inguinal hernia repair approaches

Table 2 Baseline information of patients with inguinal hernia

Table 3 Timing of outcome measurement assessment

Table 4 Causal relationship between surgery and adverse events

Additional file 1: SPIRIT Checklist

Additional file 2: Trial committee organization and contributions and role. Description of data: trial committee organization and contributions and role in accordance with SPIRIT (Standard Protocol Items: Recommendations for Interventional Trials) 2013 Checklist.

Additional file 3: Informed Consent to participate

Additional file 4: List of ethical approval documents

For peer review only - http://bmjopen.bmj.com/site/about/guidelines.xhtml

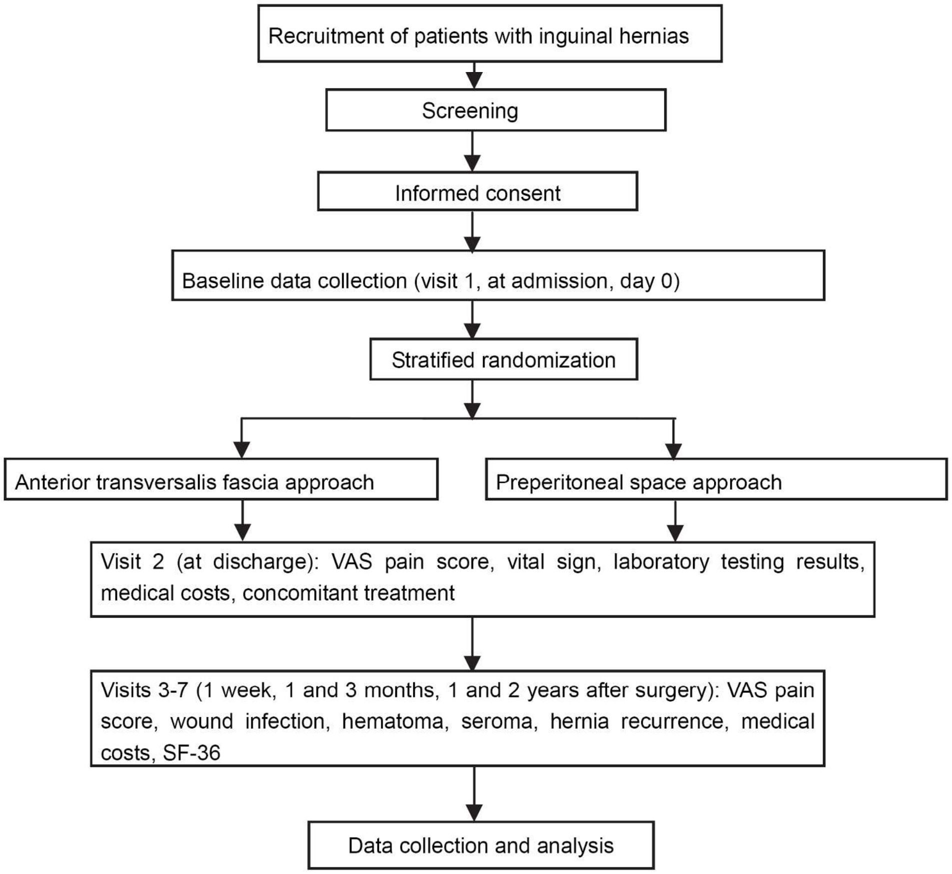

Flow chart of study protocol

184x169mm (300 x 300 DPI)

For peer review only - http://bmjopen.bmj.com/site/about/guidelines.xhtml

Additional file 1: SPIRIT Checklist

SPIRIT
STANDARD PROTOCOL ITEMS: RECOMMENDATIONS FOR INTERVENTIONAL TRIALS

SPIRIT 2013 Checklist: Recommended items to address in a clinical trial protocol and related documents*

Section/item	Item No	Description	Addressed on page number
**Administrative information**			
Title	1	Descriptive title identifying the study design, population, interventions, and, if applicable, trial acronym	p. 1
Trial registration	2a	Trial identifier and registry name. If not yet registered, name of intended registry	p. 5
	2b	All items from the World Health Organization Trial Registration Data Set	N/A
Protocol version	3	Date and version identifier	p. 37
Funding	4	Sources and types of financial, material, and other support	p. 49
Roles and responsibilities	5a	Names, affiliations, and roles of protocol contributors	p. 49
	5b	Name and contact information for the trial sponsor	p. 49
	5c	Role of study sponsor and funders, if any, in study design; collection, management, analysis, and interpretation of data; writing of the report; and the decision to submit the report for publication, including whether they will have ultimate authority over any of these activities	p. 49
	5d	Composition, roles, and responsibilities of the coordinating centre, steering committee, endpoint adjudication committee, data management team, and other individuals or groups overseeing the trial, if applicable (see Item 21a for data monitoring committee)	Additional file 2
**Introduction**			

For peer review only

Background and rationale	6a	Description of research question and justification for undertaking the trial, including summary of relevant studies (published and unpublished) examining benefits and harms for each intervention	p. 7
	6b	Explanation for choice of comparators	p. 11
Objectives	7	Specific objectives or hypotheses	p. 10-11
Trial design	8	Description of trial design including type of trial (eg, parallel group, crossover, factorial, single group), allocation ratio, and framework (eg, superiority, equivalence, noninferiority, exploratory)	p. 11-12
**Methods: Participants, interventions, and outcomes**			
Study setting	9	Description of study settings (eg, community clinic, academic hospital) and list of countries where data will be collected. Reference to where list of study sites can be obtained	p. 11
Eligibility criteria	10	Inclusion and exclusion criteria for participants. If applicable, eligibility criteria for study centres and individuals who will perform the interventions (eg, surgeons, psychotherapists)	p. 14
Interventions	11a	Interventions for each group with sufficient detail to allow replication, including how and when they will be administered	p. 15
	11b	Criteria for discontinuing or modifying allocated interventions for a given trial participant (eg, drug dose change in response to harms, participant request, or improving/worsening disease)	p. 15
	11c	Strategies to improve adherence to intervention protocols, and any procedures for monitoring adherence (eg, drug tablet return, laboratory tests)	p. 15
	11d	Relevant concomitant care and interventions that are permitted or prohibited during the trial	p. 17-18
Outcomes	12	Primary, secondary, and other outcomes, including the specific measurement variable (eg, systolic blood pressure), analysis metric (eg, change from baseline, final value, time to event), method of aggregation (eg, median, proportion), and time point for each outcome. Explanation of the clinical relevance of chosen efficacy and harm outcomes is strongly recommended	p. 23-25
Participant timeline	13	Time schedule of enrolment, interventions (including any run-ins and washouts), assessments, and visits for participants. A schematic diagram is highly recommended (see Figure)	p. 18-23, Figure 1

For peer review only - http://bmjopen.bmj.com/site/about/guidelines.xhtml

Sample size	14	Estimated number of participants needed to achieve study objectives and how it was determined, including clinical and statistical assumptions supporting any sample size calculations	p. 33
Recruitment	15	Strategies for achieving adequate participant enrolment to reach target sample size	p. 13

Methods: Assignment of interventions (for controlled trials)

Allocation:			
Sequence generation	16a	Method of generating the allocation sequence (eg, computer-generated random numbers), and list of any factors for stratification. To reduce predictability of a random sequence, details of any planned restriction (eg, blocking) should be provided in a separate document that is unavailable to those who enrol participants or assign interventions	p. 15
Allocation concealment mechanism	16b	Mechanism of implementing the allocation sequence (eg, central telephone; sequentially numbered, opaque, sealed envelopes), describing any steps to conceal the sequence until interventions are assigned	p. 15
Implementation	16c	Who will generate the allocation sequence, who will enrol participants, and who will assign participants to interventions	p.15
Blinding (masking)	17a	Who will be blinded after assignment to interventions (eg, trial participants, care providers, outcome assessors, data analysts), and how	p. 15
	17b	If blinded, circumstances under which unblinding is permissible, and procedure for revealing a participant's allocated intervention during the trial	p. 15

Methods: Data collection, management, and analysis

Data collection methods	18a	Plans for assessment and collection of outcome, baseline, and other trial data, including any related processes to promote data quality (eg, duplicate measurements, training of assessors) and a description of study instruments (eg, questionnaires, laboratory tests) along with their reliability and validity, if known. Reference to where data collection forms can be found, if not in the protocol	p. 37-38
	18b	Plans to promote participant retention and complete follow-up, including list of any outcome data to be collected for participants who discontinue or deviate from intervention protocols	p. 36-37

For peer review only - http://bmjopen.bmj.com/site/about/guidelines.xhtml

Data management	19	Plans for data entry, coding, security, and storage, including any related processes to promote data quality (eg, double data entry; range checks for data values). Reference to where details of data management procedures can be found, if not in the protocol	p. 37-38
Statistical methods	20a	Statistical methods for analysing primary and secondary outcomes. Reference to where other details of the statistical analysis plan can be found, if not in the protocol	p. 31-32
	20b	Methods for any additional analyses (eg, subgroup and adjusted analyses)	p. 32-33
	20c	Definition of analysis population relating to protocol non-adherence (eg, as randomised analysis), and any statistical methods to handle missing data (eg, multiple imputation)	p. 32-33
**Methods: Monitoring**			
Data monitoring	21a	Composition of data monitoring committee (DMC); summary of its role and reporting structure; statement of whether it is independent from the sponsor and competing interests; and reference to where further details about its charter can be found, if not in the protocol. Alternatively, an explanation of why a DMC is not needed	p. 36-39
	21b	Description of any interim analyses and stopping guidelines, including who will have access to these interim results and make the final decision to terminate the trial	p. 41
Harms	22	Plans for collecting, assessing, reporting, and managing solicited and spontaneously reported adverse events and other unintended effects of trial interventions or trial conduct	p. 30-31
Auditing	23	Frequency and procedures for auditing trial conduct, if any, and whether the process will be independent from investigators and the sponsor	p. 40
**Ethics and dissemination**			
Research ethics approval	24	Plans for seeking research ethics committee/institutional review board (REC/IRB) approval	p. 33-35, Additional file 4
Protocol amendments	25	Plans for communicating important protocol modifications (eg, changes to eligibility criteria, outcomes, analyses) to relevant parties (eg, investigators, REC/IRBs, trial participants, trial registries, journals, regulators)	p. 37-38

For peer review only - http://bmjopen.bmj.com/site/about/guidelines.xhtml

Consent or assent	26a	Who will obtain informed consent or assent from potential trial participants or authorised surrogates, and how (see Item 32)	p. 36
	26b	Additional consent provisions for collection and use of participant data and biological specimens in ancillary studies, if applicable	N/A
Confidentiality	27	How personal information about potential and enrolled participants will be collected, shared, and maintained in order to protect confidentiality before, during, and after the trial	p. 36-37
Declaration of interests	28	Financial and other competing interests for principal investigators for the overall trial and each study site	p. 49
Access to data	29	Statement of who will have access to the final trial dataset, and disclosure of contractual agreements that limit such access for investigators	p. 49
Ancillary and post-trial care	30	Provisions, if any, for ancillary and post-trial care, and for compensation to those who suffer harm from trial participation	p. 31
Dissemination policy	31a	Plans for investigators and sponsor to communicate trial results to participants, healthcare professionals, the public, and other relevant groups (eg, via publication, reporting in results databases, or other data sharing arrangements), including any publication restrictions	p. 41-42
	31b	Authorship eligibility guidelines and any intended use of professional writers	N/A
	31c	Plans, if any, for granting public access to the full protocol, participant-level dataset, and statistical code	N/A
**Appendices**			
Informed consent materials	32	Model consent form and other related documentation given to participants and authorised surrogates	Additional file 3
Biological specimens	33	Plans for collection, laboratory evaluation, and storage of biological specimens for genetic or molecular analysis in the current trial and for future use in ancillary studies, if applicable	p. 11

*It is strongly recommended that this checklist be read in conjunction with the SPIRIT 2013 Explanation & Elaboration for important clarification on the items. Amendments to the protocol should be tracked and dated. The SPIRIT checklist is copyrighted by the SPIRIT Group under the Creative Commons "Attribution-NonCommercial-NoDerivs 3.0 Unported" license.

For peer review only - http://bmjopen.bmj.com/site/about/guidelines.xhtml

Additional file 2: Trial committee organization and contributions and role.

Principal investigator (PI) and research physicians at main study site

Organization: the Fourth Affiliated Hospital of China Medical University

Contributions and role: Principal investigator, original study design: Hang-yu Li (Email:li_hangyu@ 126.com)

Organization of Steering committee and member appointment: Qing Fan, De-wei Zhang, Da-ye Yang, Hong-wu Li, Shi-bo Wei, Liang Yang, Hang-yu Li, Fu-quan Yang, Shao-jun Zhang, Yao-qiang Wu, Wei-de An, Zhong-shu Dai, Hui-yong Jiang, Fu-rong Wang, Shi-feng Qiao

Communication and exchange of opinion with PI at each site

Preparation of IRB documents and CRF

Trial management (randomized allocation management, AE data collection at each site, participant enrollment supervision, study site inspection and visits, budget allocation and management)

Cooperation with CRO in data collection, quality control, monitoring, and analysis

Steering committee (SC)

Organization and role: All authors of this manuscript

Contributions: Protocol revision and decision on final protocol

Organization of Trial Management Committee and member appointment

Designation of participant recruitment study sites

Inspection of study progress, and decision on protocol revision, if needed

Determination of study result publication timing and method

Decision on authorship in accordance with Authorship eligibility guidelines

Trial Management Committee

Organization: PI and investigators at each clinical trial participant enrollment site

Fu-quan Yang, Shengjing Hospital of China Medical University

Shao-jun Zhang, Fengtian Hospital of Shenyang Medical College

Yao-qiang Wu, The First Hospital of Dandong City

Wei-de An, The First Affiliated Hospital of Dalian Medical University

Zhong-shu Dai, General Hospital of Benxi Steel and Iron (Group) Co., LTD, Fifth Clinical College of China Medical University

Hui-yong Jiang, General Hospital of Shenyang Military Area

Fu-rong Wang, The 202nd Hospital of PLA

Shi-feng Qiao, The First Affiliated Hospital of Liaoning Medical University

Hang-yu Li*, The Fourth Affiliated Hospital of China Medical University

Organization and role: Submission and obtaining study protocol approval from relevant IRB of each study site

Clinical trial execution following protocol (e.g. participant recruitment, enrollment, data collection, CRF

For peer review only - http://bmjopen.bmj.com/site/about/guidelines.xhtml

entry)

Collection and report of AEs

For peer review only

For peer review only - http://bmjopen.bmj.com/site/about/guidelines.xhtml

Ethics Committee of the Fourth Affiliated Hospital of China Medical University

4# CHONG-SHAN EAST ROAD, SHENYANG, P.R.CHINA 110032

Informed Consent Form for Clinical Studies

[Name of Principal Investigator] Hang-yu Li
[Name of Organization] The Fourth Affiliated Hospital of China Medical University, China
[Name of Proposal and version] Anterior transversalis fascia approach versus preperitoneal space approach for inguinal hernia repair in residents in Northern China: study protocol for a prospective, multi-center, randomized, controlled trial (Version 1.0)

This Informed Consent Form has two parts:

- **Information Sheet (to share information about the research with you)**
- **Certificate of Consent (for signatures if you agree to take part)**

You will be given a copy of the full Informed Consent Form

PART I: Information Sheet

Introduction

I am Dr. Hang-yu Li, working for the Fourth Affiliated Hospital of China Medical University. We are doing research on different surgical techniques used to repair abnormal wall defects. I am going to give you information and invite you to be part of this research. You do not have to decide today whether or not you will participate in the research. Before you decide, you can talk to anyone you feel comfortable with about the research.

There may be some words that you do not understand. Please ask me to stop as we go through the information and I will take time to explain. If you have questions later, you can ask them of me, the study doctor or the staff.

Purpose of the research

There are few reports on the effects of different inguinal hernia repair approaches on postoperative complications, particularly regarding severe postoperative complications, in patients from northern China. The incidence of severe complications after inguinal hernia repair is relatively low, but this surgery can lead to physical impairment or organ dysfunction that greatly decreases patient quality of life and places a heavy burden on the patient's family and society. This study will compare the effectiveness of inguinal hernia repair via the anterior transversalis fascia approach versus the preperitoneal approach in patients from northern China in terms of postoperative complications, quality of life, and cost-effectiveness. This study aims to determine the optimal method for inguinal hernia repair that is suitable for the anatomic features of the inguinal region for local patients.

Type of Research Intervention

Briefly state the type of intervention that will be undertaken. This will be expanded upon in the procedures section but it may be helpful and less confusing to the participant if they

For peer review only - http://bmjopen.bmj.com/site/about/guidelines.xhtml

know from the very beginning whether, for example, the research involves a vaccine, an interview, a biopsy or a series of finger pricks.
You will be undergone inguinal hernia repair via the anterior transversalis fascia approach or the preperitoneal approach. Also you will be followed-up at the following time-points: at baseline (at admission), pre-discharge, and at 1 week, 1 month, 3 months (hospital visit or telephone follow-up), 1 year (telephone follow-up), and 2 years after surgery (telephone follow-up).

Participant selection
We are inviting all male adults with inguinal hernia who attend our hospital to participate in the research.

- ***Example of question to elucidate understanding:*** *Do you know why we are asking you to take part in this study? Do you know what the study is about?*

Voluntary Participation
Your participation in this research is entirely voluntary. It is your choice whether to participate or not. Whether you choose to participate or not, all the services you receive at this hospital will continue and nothing will change. If you choose not to participate in this research project, you will offered the treatment that is routinely offered in this hospital for inguinal hernia repair, and we will tell you more about it later. You may change your mind later and stop participating even if you agreed earlier.

- ***Examples of question to elucidate understanding:*** *If you decide not to take part in this research study, do you know what your options are? Do you know that you do not have to take part in this research study, if you do not wish to? Do you have any questions?*

Include the following section only if the protocol is for a clinical trial:

Information on the different approaches for inguinal hernia repair
1) What is the anterior transversalis fascia repair?
An oblique skin incision will be made parallel to the inguinal groove from the inner to the outer inguinal ring. Subcutaneous tissue will be dissected until the external oblique aponeurosis is reached. Hemostasis will be achieved by electric coagulation. A4 – 6 cm long skin incision will be made on the external oblique aponeurosis, starting from the pubic tubercle, to fully expose the pubic tubercle and the inner ring.
The lower part of the external oblique aponeurosis will be separated from the spermatic cord. The upper part of the external oblique aponeurosis will then be separated from the internal oblique aponeurosis and dissociated to a point 3 cm above the inguinal canal wall. The overlying spermatic cord and cremaster muscle will be lifted up and disassociated about 2 cm above the pubic tubercle. The ilioinguinal nerve, external spermatic vessels, and genital nerves concomitant with the spermatic cord will be protected while the spermatic cord is lifted.
The inguinal canal will then be dissected and the herniasac will be dissociated from the surrounding tissue. The cremaster muscle fibers will be separated longitudinally and the hernia sac will be separated from the spermatic cord. The hernia sac will be dissociated until the neck of the hernia sac is reached. All abdominal contents in the hernia sac will be returned to the abdominal cavity. In cases with a small hernia sac, the sac will also be returned to the anterior peritoneal cavity to minimize postoperative pain. In cases with a large hernia sac, the distal end will be transected and opened, and the proximal part will be ligated at a high position.
A patch designed with an arc-shaped head and a swallow tail-like end that comprises two pieces (a wide top piece and a narrow bottom piece) will be sutured and fixed with non-absorbable

For peer review only - http://bmjopen.bmj.com/site/about/guidelines.xhtml

polypropylene sutures. The inner side of the patch will be sutured to the pubic tubercle and the lateral boundary of the rectus abdominis muscle. The upper border of the patch will be sutured to the conjoined tendon and its border with the inguinal ligament and iliopubic tract. The wound will then be closed using layered sutures according to anatomic position.

2) What is the preperitoneal space repair?

The skin will be cut and subcutaneous tissue will be dissected. The abdominal external oblique aponeurosis will be dissected along the fibers. The abdominal internal oblique aponeurosis and transversus abdominis muscle will be bluntly separated to expose the transverse fascia. Dissociation of the hernia sac will be done as described above in the anterior transversalis fascia repair.

Transabdominal preperitoneal or totally extraperitoneal laparoscopic techniques were used to dissociate preperitoneal space, strip spermatic cord peritoneum, anatomize and expose the pubic pore. A polypropylene patch will be placed in the prevesical space and Bogros' space to achieve repair of the pubic pore-containing area.

3) Why we compare these two approaches?

Many surgical repair methods involving patches (of varying types and materials) are available for inguinal hernia repair. At present, there is a scarcity of clinical evidence from large-sample, randomized, controlled trials investigating the effectiveness of inguinal hernia repair via the anterior transversalis fascia approach versus the preperitoneal approach. Our outcomes will provide trial-based evidence for selection of rational therapeutic regimen in the clinic.

4) What are the side-effects of the repairs?

There are few reports on the effects of different inguinal hernia repair approaches on postoperative complications, particularly regarding severe postoperative complications, in patients in northern China. The incidence of severe complications after inguinal hernia repair is relatively low, but this surgery can lead to physical impairment or organ dysfunction that greatly decreases patient quality of life and places a heavy burden on the patient's family and society.

Procedures and Protocol

You will receive the treatment of inguinal hernia repair according to national guidelines, i.e. Guidelines for the Diagnosis and Treatment of Inguinal Hernia in Adults (2014 Edition) formulated by Chinese national society and the Adult Inguinal Hernia Treatment Guidelines (Updated Edition in 2014) developed by the European Hernia Society. This means that you will be treated either by the anterior transversalis fascia approach or the preperitoneal approach during the surgery.

In strict accordance with the Consolidated Standards of Reporting Trials (CONSORT) standards, the baseline data (preoperative data), therapeutic regimen, therapeutic outcome, and medical costs during hospitalization of patients with inguinal hernia will be recorded. Patient data will be collected using an electronic data capture system (EDC).

Description of the Process

During the research, you make seven visits to the hospital.

Visit 1 (at admission, day 0):: you will be requested to

- Sign informed consent
- Recheck inclusion/exclusion criteria
- Demographic data (sex, age, body height, body mass index) and medical insurance type
- Type of hernia (indirect or direct)
- Inguinal hernia classification as type I, II, III, or IV according to the adult inguinal hernia and femoral hernia classification (revised in 2003) developed by the Hernia and Abdominal Wall Surgery Group, Society of Surgery, Chinese Medical Association [12]

For peer review only - http://bmjopen.bmj.com/site/about/guidelines.xhtml

- ASA classification
- History of diseases and risk factors* (diabetes mellitus, cardiovascular disease, lung disease, peripheral vascular disease, dementia, hypertension, smoking history and alcohol use history) (*optional evaluation items)
- Disease onset and admission (interval from first appearance of the lump, main symptoms)
- Imaging examination (ultrasound, CT)
- Laboratory examination (routine blood testing, coagulation testing, testing of blood glucose, lipids and electrolytes, and hepatic and renal function)
- Vital signs (body temperature, pulse, respiration rate, blood pressure)
- VAS pain score
- Electrocardiography
- Concomitant treatment

Visit 2 (Pre-discharge): you will be tested

- Vital signs (body temperature, pulse, respiration rate, blood pressure)
- VAS pain score
- Laboratory examination (routine blood testing, coagulation testing, testing of blood glucose, lipids and electrolytes, and hepatic and renal function)
- Treatment regimen (inguinal hernia-specific treatment)

Surgical approach and patches (type and fixation method); anesthesia method; drug name (generic preferred), dosage, route, start date, length of drug use will be recorded.

- Concomitant treatment: Inguinal hernia-specific treatment, maintenance therapy, and drugs used for concomitant treatment.

Visit 3 (1 week after surgery via clinic visit or telephone follow-up): you will be tested

- VAS pain score
- 36-Item Short Form Health Survey (SF-36) score
- Hernia recurrence
- Presence or absence of hematoma and/or seroma
- Wound infection

Visit 4 (1 month after surgery via clinic visit or telephone follow-up): you will be tested

- VAS pain score
- SF-36 score
- Presence or absence of hematoma and/or seroma
- Wound infection

Visit 5 (3 months after surgery via clinic visit or telephone follow-up): you will be tested

- VAS pain score
- SF-36 score
- Hernia recurrence
- Presence or absence of hematoma and/or seroma
- Wound infection

For peer review only

Visit 6 (1 year after surgery via telephone follow-up): you will be tested

- VAS pain score
- SF-36 score
- Hernia recurrence
- Presence or absence of hematoma and/or seroma
- Wound infection

Visit 7 (2 years after surgery via telephone follow-up): you will be tested

- VAS pain score
- SF-36 score
- Hernia recurrence
- Presence or absence of hematoma and/or seroma
- Wound infection

Duration

In total, you will be asked to come 7 times to the hospital in 2 years. At the end of 2 years, the research will be finished.

- ***Examples of question to elucidate understanding:*** *Can you tell me if you remember the number of times that we are asking you to come to the hospital to complete the treatment? The research project? How many injections will you be given? How many tablets? How much blood will be taken from your veins, using a syringe and needle? Over how many weeks? Etc. Do you have any other questions? Do you want me to go through the procedures again?*

Side Effects

The incidence of severe complications after inguinal hernia repair is relatively low, any adverse medical events occurring after herniorrhaphy in patients or clinical subjects. This surgery could lead to physical impairment or organ dysfunction that decreases patient quality of life.

Serious adverse postoperative medical events involving one or more of the following criteria:

- Death, irrespective of the cause
- Life-threatening event
- Severe or permanent disability or organ dysfunction
- Hemorrhage
- Malformation, birth defect
- Hospitalization or extended hospital stay
- Re-admission to hospital
- Recurrence (symptomatic) of inguinal hernia

Risks

History of diabetes mellitus, cardiovascular disease, lung disease, peripheral vascular disease, dementia, hypertension, smoking history and alcohol use history could cause possible risks, including wound infection, rates of hematoma, seroma, hernia recurrence, intestinal obstruction, intestinal fistula, entocele, mesenteric thrombosis, femoral vein thrombosis, ischemic orchitis, painful ejaculation, varicocele, scrotal edema and etc.

While the possibility of this happening is very low, you should still be aware of the possibility. We will try to decrease the chances of this event occurring, but if something unexpected happens

For peer review only

- ***Examples of question to elucidate understanding:*** *Do you understand that these risks can happen whether or not you are in the research study? Etc. Do you have any other questions?*

Benefits
There may not be any benefit for you but your participation is likely to help us find the answer to the research question.

Reimbursements
We will waive the medical cost associated during your follow-up visit. You will not be given any other money or gifts to take part in this research.

- ***Examples of question to elucidate understanding:*** *Can you tell me if you have understood correctly the benefits that you will have if you take part in the study? Do you have any other questions?*

Confidentiality
We will not be sharing the identity of those participating in the research. The information that we collect from this research project will be kept confidential. Information about you that will be collected during the research will be put away and no-one but the researchers will be able to see it. Any information about you will have a number on it instead of your name. Only the researchers will know what your number is and we will lock that information up in the computer. It will not be shared with or given to anyone except your clinician.

- ***Example of question to elucidate understanding:*** *Did you understand the procedures that we will be using to make sure that any information that we as researchers collect about you will remain confidential? Do you have any questions about them?*

Sharing the Results
The results of this study will be disseminated via peer-reviewed publications and conference presentations. No data are available at the moment. Confidential information will not be shared.

Right to Refuse or Withdraw
You do not have to take part in this research if you do not wish to do so and refusing to participate will not affect your treatment at this hospital in any way. You will still have all the benefits that you would otherwise have at this hospital. You may stop participating in the research at any time that you wish without losing any of your rights as a patient here. Your treatment at this hospital will not be affected in any way.

Alternatives to Participating
If you do not wish to take part in the research, you will be provided with the anterior transversalis fascia repair at the hospital.

Who to Contact
If you have any questions you may ask them now or later, even after the study has started. If you wish to ask questions later, you may contact any of the following:
Name: Dr. Yang
Address: 4# Chong-shan East Road, Shenyang
Telephone number: (024)62255001

For peer review only - http://bmjopen.bmj.com/site/about/guidelines.xhtml

E-mail: 529687607@qq.com

This proposal has been reviewed and approved by Ethics Committee of the Fourth Affiliated Hospital of China Medical University, which is a committee whose task it is to make sure that research participants are protected from harm. If you wish to find about more about the IRB, contact [Dr. Yuan-zhe Jin, 4# Chong-shan East Road, Shenyang, 024-62043027.]).

- ***Example of question to elucidate understanding:*** *Do you know that you do not have to take part in this study if you do not wish to? You can say No if you wish to? Do you know that you can ask me questions later, if you wish to? Do you know that I have given the contact details of the person who can give you more information about the study?*

You can ask me any more questions about any part of the research study, if you wish to. Do you have any questions?

PART II: Certificate of Consent

I have read the foregoing information, or it has been read to me. I have had the opportunity to ask questions about it and any questions that I have asked have been answered to my satisfaction. I consent voluntarily to participate as a participant in this research.

Print Name of Participant__________________

Signature of Participant ___________________

Date ___________________________

Day/month/year

If illiterate

A literate witness must sign (if possible, this person should be selected by the participant and should have no connection to the research team). Participants who are illiterate should include their thumb-print as well.

I have witnessed the accurate reading of the consent form to the potential participant, and the individual has had the opportunity to ask questions. I confirm that the individual has given consent freely.

Print name of witness______________________ **OR** **Thumb print of participant**

Signature of witness ______________________

Date _________________________

Day/month/year

Statement by the researcher/person taking consent

I have accurately read out the information sheet to the potential participant, and to the best of my ability made sure that the participant understands that the inguinal hernia repair will be performed and a few follow-up visits requested to finish the study.

For peer review only - http://bmjopen.bmj.com/site/about/guidelines.xhtml

I confirm that the participant was given an opportunity to ask questions about the study, and all the questions asked by the participant have been answered correctly and to the best of my ability. I confirm that the individual has not been coerced into giving consent, and the consent has been given freely and voluntarily.

A copy of this ICF has been provided to the participant.

Print Name of Researcher/person taking the consent________________________

Signature of Researcher /person taking the consent__________________________

Date ____________________________
Day/month/year

For peer review only

Additional file 4: List of ethical approval documents

Center	Name of ethical bodies	Ethical approval reference number
Department of General Surgery, the Fourth Affiliated Hospital of China Medical University	The Ethics Committee of the Fourth Affiliated Hospital of China Medical University	2015-027
Department of General Surgery, Branch 3, First Hospital of Dalian Medical University	The Ethics Committee of First Hospital of Dalian Medical University	2016-034
Department of General Surgery, the 202 Hospital of Chinese PLA	The Ethics Committee of the 202 Hospital of Chinese PLA	2015-033
Ward of Hernia, Department of General and Gastrointestinal Surgery, First Affiliated Hospital of Liaoning Medical University	The Ethics Committee of First Affiliated Hospital of Liaoning Medical University	2016-022
Department of General Surgery, General Hospital of Shenyang Military	The Ethics Committee of General Hospital of Shenyang Military	2015-015
Second Department of General Surgery, General Hospital of Benxi Iron and Steel Co., Ltd.	The Ethics Committee of General Hospital of Benxi Iron and Steel Co., Ltd.	2015-011
First Department of General	The Ethics Committee of Affiliated	2015-022

For peer review only

Surgery, Affiliated Central Hospital of Shenyang Medical University	Central Hospital of Shenyang Medical University	
Department of General Surgery, First Hospital of Dandong	The Ethics Committee of First Hospital of Dandong	2016-016
Shengjing Hospital of China Medical University	The Ethics Committee of Shengjing Hospital of China Medical University	2016-010

For peer review only

BMJ Open

Anterior transversalis fascia approach versus preperitoneal space approach for inguinal hernia repair in residents in Northern China: study protocol for a prospective, multi-center, randomized, controlled trial

Journal:	*BMJ Open*
Manuscript ID	bmjopen-2017-016481.R2
Article Type:	Protocol
Date Submitted by the Author:	04-Jul-2017

Complete List of Authors:	Fan, Qing; The Fourth Affiliated Hospital of China Medical University<br>Zhang, Dewei; The Fourth Affiliated Hospital of China Medical University<br>Yang, Daye; The Fourth Affiliated Hospital of China Medical University<br>Li, Hongwu; The Fourth Affiliated Hospital of China Medical University<br>Wei , Shibo; The Fourth Affiliated Hospital of China Medical University<br>Yang, Liang ; The Fourth Affiliated Hospital of China Medical University<br>Yang, Fuquan ; Shengjing Hospital of China Medical University<br>Zhang, Shaojun ; Fengtian Hospital of Shenyang Medical College<br>Wu, Yaoqiang ; The First Hospital of Dandong City<br>An, Weide ; The First Affiliated Hospital of Dalian Medical University<br>Dai, Zhongshu ; General Hospital of Benxi Steel and Iron (Group) Co., LTD, Fifth Clinical College of China Medical University<br>Jiang, Huiyong ; General Hospital of Shenyang Military Area<br>Wang, Furong ; The 202nd Hospital of PLA<br>Qiao, Shifeng ; The First Affiliated Hospital of Liaoning Medical University<br>Li, Hangyu; The Fourth Affiliated Hospital of China Medical University

**Primary Subject Heading**:	Surgery
Secondary Subject Heading:	Renal medicine
Keywords:	inguinal hernia, anterior transversalis fascia repair, preperitoneal repair, quality of life, cost-utility analysis, randomized

SCHOLARONE™ Manuscripts

Anterior transversalis fascia approach versus preperitoneal space approach for inguinal hernia repair in residents in Northern China: study protocol for a prospective, multi-center, randomized, controlled trial

Qing Fan[1], De-wei Zhang[1], Da-ye Yang[1], Hong-wu Li[1], Shi-bo Wei[1], Liang Yang[1], Fu-quan Yang[2], Shao-jun Zhang[3], Yao-qiang Wu[4], Wei-de An[5], Zhong-shu Dai[6], Hui-yong Jiang[7], Fu-rong Wang[8], Shi-feng Qiao[9], Hang-yu Li[1*]

Qing Fan The Fourth Affiliated Hospital of China Medical University, Shenyang, Liaoning Province, China Email: 18900913745@163.com

De-wei Zhang The Fourth Affiliated Hospital of China Medical University, Shenyang, Liaoning Province, China Email: syzhangdewei@sohu.com

Da-ye Yang The Fourth Affiliated Hospital of China Medical University, Shenyang,Liaoning Province, China Email:yangdaye73@126.com

Hong-wu Li The Fourth Affiliated Hospital of China Medical University, Shenyang, Liaoning Province, China Email: 13998201180@163.com

Shi-bo Wei The Fourth Affiliated Hospital of China Medical University, Shenyang, Liaoning Province, China Email: 13898111626@163.com

Liang Yang The Fourth Affiliated Hospital of China Medical University, Shenyang, Liaoning Province, China Email: 529687607@qq.com

For peer review only - http://bmjopen.bmj.com/site/about/guidelines.xhtml

Fu-quan Yang Shengjing Hospital of China Medical University, Shenyang, Liaoning Province, China Email: yangfq@sj-hospital.org

Shao-jun Zhang Fengtian Hospital of Shenyang Medical College, Shenyang, Liaoning Province, China Email: shaojunzhang@163.com

Yao-qiang Wu The First Hospital of Dandong City, Dandong, Liaoning Province, China Email:wuyaoqiang@sina.com

Wei-de An The First Affiliated Hospital of Dalian Medical University, Dalian, Liaoning Province, China Email: anweide@sina.com

Zhong-shu Dai General Hospital of Benxi Steel and Iron (Group) Co., LTD, Fifth Clinical College of China Medical University, Benxi, Liaoning Province, China Email: dzs20092009@163.com

Hui-yong Jiang General Hospital of Shenyang Military Area, Shenyang, Liaoning Province, China Email: huiyongj@126.com

Fu-rong Wang The 202nd Hospital of PLA, Shenyang, Liaoning Province, China Email: furong_jiyu@126.com

Shi-feng Qiao The First Affiliated Hospital of Liaoning Medical University, Jinzhou, Liaoning Province, China Email: shifengqiao2013@163.com

Hang-yu Li[*] The Fourth Affiliated Hospital of China Medical University, Shenyang, Liaoning Province, China Email:li_hangyu@126.com

For peer review only - http://bmjopen.bmj.com/site/about/guidelines.xhtml

***Correspondence to**: Hang-yu Li, li_hangyu@126.com

For peer review only

For peer review only - http://bmjopen.bmj.com/site/about/guidelines.xhtml

Abstract

Introduction: Many surgical techniques have been used to repair abdominal wall defects in the inguinal region based on the anatomic characteristics of this region, and can be categorized as 'tension' repair or 'tension-free' repair. Tension-free repair is the preferred technique for inguinal hernia repair. Tension-free repair of inguinal hernia can be performed through either the anterior transversalis fascia approach or the preperitoneal space approach. There are few large-sample, randomized, controlled trials investigating the curative effects of the anterior transversalis fascia approach versus the preperitoneal space approach for inguinal hernia repair in patients in northern China.

Methods and analysis: This will be a prospective, large-sample, multicenter, randomized, controlled trial. Registration date is December 1, 2016. Actual Study Start Date is February 6, 2017. Estimated Study Completion Date is June 2020.A cohort of over 720 patients with inguinal hernias will be recruited from 9 institutions in Liaoning Province, China. Patient randomization will be stratified by center to undergo inguinal hernia repair via the anterior transversalis fascia approach or the preperitoneal approach. Primary and secondary outcome assessments will be performed at baseline (prior to surgery), pre-discharge, and at postoperative 1 week, 1 month, 3 months, 1 year and 2 years. The primary outcome is the incidence of postoperative chronic

For peer review only - http://bmjopen.bmj.com/site/about/guidelines.xhtml

inguinal pain. The secondary outcome is postoperative complications (including rates of wound infection, hematoma, seroma, and hernia recurrence).

Ethics and dissemination: This trial will be conducted in accordance with the Declaration of Helsinki and supervised by the institutional review board of the Fourth Affiliated Hospital of China Medical University (approval number 2015-027). All patients will receive information about the trial in verbal and written forms and will give informed consent before enrollment. The results will be published in peer-reviewed journals or disseminated through conference presentations.

Trial registration number NCT02984917; Pre-results.

Keywords: inguinal hernia; anterior transversalis fascia repair; preperitoneal repair; quality of life; cost-utility analysis; randomized controlled trial

Strengths and limitations of this study

● This trial will be the first prospective multicenter randomized controlled study involving 9 institutions in Liaoning province of Northern China to provide reliable results from a representative study population.

● This trial will compare postoperative complications after anterior transversalis fascia approach versus preperitoneal space approach for inguinal hernia repair in residents in Northern China.

● Based on ethical and economic considerations, interim analysis will be performed during the trial to reduce unnecessary waste of manpower and materials (lower cost).

For peer review only - http://bmjopen.bmj.com/site/about/guidelines.xhtml

● The limited time for studying postoperative recurrence of hernia likely influences the judgment of long-term recurrence of inguinal hernia.

● Variability of surgeons from each study center could be a major confounder to bias the results of this trial.

For peer review only

For peer review only - http://bmjopen.bmj.com/site/about/guidelines.xhtml

BACKGROUND

History and current related studies

Inguinal hernia is a common surgical disease that manifests as protrusion of abdominal cavity contents through the inguinal canal because of an abdominal wall defect. It is more common in males than in females, with an overall incidence of 5–10%[1]. Methods for surgical repair of abdominal wall defects in the inguinal region are classified as either ‘tension’ repairs or ‘tension-free’ repairs. Herniorrhaphy through repair of the posterior wall of the inguinal canal was first described by Bassini in 1887, and is regarded as a classic surgical method[2].

As understanding of the anatomic location and pathophysical characteristics of inguinal hernia developed, the American surgeon Lichtenstein proposed a new concept of tension-free herniorrhaphy[3]. This technique was quickly adopted worldwide because of its advantages including minimal invasion, technical ease, effectiveness, low complication rate, low recurrence rate, and allowance of resumption of unrestricted physical activity. The most common technique is open tension-free herniorrhaphy.

Tension-free herniorrhaphy methods include anterior transversalis fascia repair, preperitoneal repair, abdominal cavity patch repair and combined repair approaches[4-6]. Lichtenstein herniorrhaphy is the representative technique of anterior transversalis fascia repair. Preperitoneal repair techniques include transabdominal preperitoneal,

For peer review only - http://bmjopen.bmj.com/site/about/guidelines.xhtml

total extraperitoneal, and Kugel repair techniques. The combined repair approaches refer to tension-free herniorrhaphy using a modified Kugel patch and the Ultrapro hernia system[7-8].

Many surgical repair methods involving patches (of varying types and materials) are available for inguinal hernia repair. Zhu et al.[9] performed a Meta analysis regarding open extraperitoneal approachand extraperitoneal laparoscopic hernioplasty for inguinal hernia repair. They found that these two approaches exhibited basically similar clinical outcomes. Patients receiving extraperitoneal laparoscopic hernioplasty needed shorter hospital stays and exhibited lower incidence of postoperative complications. Patients receiving open extraperitoneal approach exhibited lower incidence of peritoneal tears. Pisanu et al.[10] analyzed the clinical efficacy of laparoscopic and Lichtenstein techniques in recurrent inguinal hernia repair. They found that laparoscopicyshowed lower indicience of chronic inguinal pain and an earlier return to normal daily activities but greatly longer operative time. There are many randomized controlled trials[11-15] on the clinical efficacy of inguinal hernia repair approaches (Table 1), but little is reported on anterior transversalis fascia approach and preperitoneal space approach for inguinal hernia repair in residents in Northern China.

Table 1 Randomized controlled trials (RCTs) regarding inguinal hernia repair approaches

For peer review only - http://bmjopen.bmj.com/site/about/guidelines.xhtml

Study	Design	Subjects	Disease	Follow-up time	Outcome measures	Conclusion
Akhtar et al.[11]	RCT	TAPP (n =30) Lichtenstein (n =50)	Unilateral inguinal hernia	6 months	Average operation, pain score, analgesics, admission days, days required to return to work	Laparoscopic hernia surgery is better than Lichtenstein repair in terms of postoperative pain, hospital stay and return to daily activity.
Sarhan et al.[12]	RCT	A total of 200 patients scheduled for unilateral inguinal hernia repair were randomly divided intotwo groups to undergo either laparoscopic TAPP (group A) or open modified Kugel procedure	Unilateral inguinal hernia	32 months	Recurrence and short-term and long-term complications	Both open modified Kugel and laparoscopic TAPP preperitoneal repair techniques for inguinal hernia are safe andeffective with low recurrence rates. Laparoscopic approach has better outcome in terms of chronic pain, short operative time, and short duration of hospital stays.
Kargar et al.[13]	RCT	TAPP (n =60) Lichtenstein (n =60)	Inguinal hernia	Follow-up occurred within 6 weeks.	Pain score (VAS), hematoma/seroma, urinary retention, wound infection, hospital stay	The laparoscopic TAPP repair is safer and less complicated approach for inguinal hernia repair. The two main short-term advantages of the laparoscopic TAPP repair with the tension free Lichtenstein repair were less postoperative pain and earlier return to the normal life activities. No difference was seen in overall complications.
Salma et al.[14]	RCT	TAPP (n =30) Lichtenstein (n =30)	Direct inguinal hernia	Post-operative pain intensity assessed by VAS and hospital stay measured in hours.	Hospital stay, immediate post-operative pain	There is less postoperative pain after laparoscopic repair but hospital stay is same in both the procedures but laparoscopic procedure does increase the cost.
Bahram[15]	RCT	TAPP (n =150) Lichtenstein (n =150)	Inguinal hernia	Three hundred patients with inguinal hernia were enrolled in this study, divided into two equal groups: Group [illegible]	Operative time, intra-operative visceral injury, ileus, hospital stay or wound complications, post-operative pain, groin	TAPP technique is an excellent approach for treatment of inguinal hernia in comparison to LR either unilateral or bilateral, primary or

For peer review only - http://bmjopen.bmj.com/site/about/guidelines.xhtml

				managed by trans-abdominal pre-peritoneal laparoscopic repair (TAPP) and Group II managed by open lichtenstein repair.	hypothesia, return to activities, recurrence	recurrent inguinal hernia with low morbidity and recurrence comparable to that oflichtenstein repair with advantages of less post-operative pain and early return to activities.

TAPP: transabdominal preperitoneal.

There are few reports on the effects of different inguinal hernia repair approaches on postoperative complications, particularly regarding severe postoperative complications, in patients in northern China. The incidence of severe complications after inguinal hernia repair is relatively low, but this surgery can lead to physical impairment or organ dysfunction that greatly decreases patient quality of life and places a heavy burden on the patient's family and society. At present, there is a scarcity of clinical evidence from large-sample, randomized, controlled trials investigating the effectiveness of inguinal hernia repair via the anterior transversalis fascia approach versus the preperitoneal approach.

Main objectives

In this study, we will investigate the advantages and disadvantages of the anterior transversalis fascia approach versus the preperitoneal approach for inguinal hernia repair in residents from northern China regarding common postoperative complications

For peer review only - http://bmjopen.bmj.com/site/about/guidelines.xhtml

(including acute and chronic pain, wound infection, rates of wound infection, hematoma, seroma, and hernia recurrence) and severe postoperative complications. These outcomes will provide trial-based evidence for selection of rational therapeutic regimen in the clinic.

Distinguishing features from related studies

(1) This study will use center-based stratification to investigate the effects of anterior transversalis fascia approach versus preperitoneal space approach for inguinal hernia repair on postoperative chronic inguigal pain and other common complications. This study will determine the optimal surgical hernia repair approach that is suitable for the anatomic characteristics of the inguinal region of residents in northern China and corresponds to the regional economic conditions. (2) Cost-utility analysis will be analyzed using center-based stratification. (3) To analyze the effects of different surgical repair approaches (involving various patch types and materials) on postoperative quality of life.

Methods/Design

Study design

This is a prospective, large-sample, multi-center, randomized, controlled trial that will

For peer review only - http://bmjopen.bmj.com/site/about/guidelines.xhtml

include a cohort of over 720 patients with inguinal hernia. The Trial committee organization and contributions and role are provided in the additional file 1 (Additional file 1). In strict accordance with the Consolidated Standards of Reporting Trials (CONSORT) standards[16], the baseline data (preoperative data), therapeutic regimen, therapeutic outcome, and medical costs during hospitalization of patients with inguinal hernia will be recorded. Patient data will be collected using an electronic data capture system (EDC).

According to recommendations for treatment and follow-up of inguinal hernia repair in adults in the Guidelines for the Diagnosis and Treatment of Inguinal Hernia in Adults (2014 Edition) formulated by Chinese scholars[17] and the Adult Inguinal Hernia Treatment Guidelines (Updated Edition in 2014) developed by the European Hernia Society[18], patients who undergo herniorrhaphy will be followed-up at seven time-points: at baseline (at admission, visit 1), pre-discharge, and at 1 week (visit 2), 1 month (visit 3), 3 months (visit 4, clinic visit or telephone follow-up), 1 year (visit 5, telephone follow-up), and 2 years after surgery (visit 6, telephone follow-up). The flow chart of the study protocol is shown in Figure 1. Prior to surgery: patients will be re-screened against inclusion and exclusion criteria. Signed informed consent will be obtained. Patient's demographic data, history of disease and medication, and admission condition and vital signs will be recorded. Clinical examination data will be collected

For peer review only - http://bmjopen.bmj.com/site/about/guidelines.xhtml

from each center, including history of disease, physical examination, laboratory testing results, imaging findings, preoperative VAS pain score, intraoperative findings, and details of occurrence and management methods of intraoperative injury to the intestinal tract and bladder, spermatic cord, and vascular system.

· Pre-discharge, and 1 week, 1 and 3 months, 1 and 2 years after surgery: pain, wound infection, hematoma and seroma in the inguinal region, and hernia recurrence will be recorded. Medical costs during hospitalization and patient quality of life after discharge will be also recorded. The flow chart of study protocol is shown in Figure 1. And The Standard Protocol Items: Recommendations for Interventional trials (SPIRIT checklist) was followed in designing the study protocol (Additional file 2).

Patients

Patients with inguinal hernia will be recruited from nine trial centers in northern China: the Department of General Surgery, the Fourth Affiliated Hospital of China Medical University; Department of General Surgery, Branch 3, First Hospital of Dalian Medical University; Department of General Surgery, the 202 Hospital of Chinese PLA; Ward of Hernia, Department of General and Gastrointestinal Surgery, First Affiliated Hospital of Liaoning Medical University; Department of General Surgery, General Hospital of Shenyang Military; Second Department of General Surgery, General Hospital of Benxi

For peer review only - http://bmjopen.bmj.com/site/about/guidelines.xhtml

Iron and Steel Co., Ltd.; First Department of General Surgery, Affiliated Central Hospital of Shenyang Medical University; Department of General Surgery, First Hospital of Dandong; Shengjing Hospital of China Medical University.

Inclusion criteria

Male patients presenting with all of the following conditions will be considered for study admission:

· Diagnosed with primary unilateral inguinal hernia

· Aged 18–80 years

· American Society of Anesthesiologists (ASA) classification I–II

· Provision of informed consent

Exclusion criteria

Patients with any one or more of the following will be excluded from this study:

· Severe organ dysfunction or inability to tolerate surgery

· Hernia recurrence

· Giant hernia (inner size of the hernia > 4 cm)

· Scrotal hernia

· Incarcerated inguinal hernia

For peer review only - http://bmjopen.bmj.com/site/about/guidelines.xhtml

For peer review only

· Inability to complete follow-up or questionnaire because of mental disorder or other reasons

· History of preperitoneal surgery, such as radical prostatectomy

Randomization and blinding

This study is a multi-center trial, so stratified block randomization will be performed in each center. A randomization sequence table will be generated by a statician who will not be involved in the trial using Statistical Analysis System (SAS 9.1). The serial numbers assigned to each patient will be preserved in opaque sealed envelopes. The sealedenvelopes will be subsequently given to the trial center. All patients will not know the surgical regimen until after the surgery. The surgeons will not be blinded to the surgical regimen. Outcome assessors will be blinded to the surgical records in the electronic case report form (eCRF).

Interventions

Based on recommendations for treatment and follow-up of inguinal hernias in adults made by the Chinese Medical Association and Chinese Medical Doctor Association[17] and the European Hernia Society[18].

For peer review only - http://bmjopen.bmj.com/site/about/guidelines.xhtml

Anterior transversalis fascia repair

An oblique skin incision will be made parallel to the inguinal groove from the inner to the outer inguinal ring. Subcutaneous tissue will be dissected until the external oblique aponeurosis is reached. Hemostasis will be achieved by electric coagulation. A4–6 cm long skin incision will be made on the external oblique aponeurosis, starting from the pubic tubercle, to fully expose the pubic tubercle and the inner ring.

The lower part of the external oblique aponeurosis will be separated from the spermatic cord. The upper part of the external oblique aponeurosis will then be separated from the internal oblique aponeurosis and dissociated to a point 3 cm above the inguinal canal wall. The overlying spermatic cord and cremaster muscle will be lifted up and disassociated about 2 cm above the pubic tubercle. The ilioinguinal nerve, external spermatic vessels, and genital nerves concomitant with the spermatic cord will be protected while the spermatic cord is lifted.

The inguinal canal will then be dissected and the herniasac will be dissociated from the surrounding tissue. The cremaster muscle fibers will be separated longitudinally and the hernia sac will be separated from the spermatic cord. The hernia sac will be dissociated until the neck of the hernia sac is reached. All abdominal contents in the hernia sac will be returned to the abdominal cavity. In cases with a small hernia sac, the sac will also be returned to the anterior peritoneal cavity to minimize postoperative

For peer review only - http://bmjopen.bmj.com/site/about/guidelines.xhtml

pain. In cases with a large hernia sac, the distal end will be transected and opened, and the proximal part will be ligated at a high position.

A patch designed with an arc-shaped head and a swallow tail-like end that comprises two pieces (a wide top piece and a narrow bottom piece) will be sutured and fixed with non-absorbable polypropylene sutures. The inner side of the patch will be sutured to the pubic tubercle and the lateral boundary of the rectus abdominis muscle. The upper border of the patch will be sutured to the conjoined tendon and its border with the inguinal ligament and iliopubic tract. The wound will then be closed using layered sutures according to anatomic position.

Preperitoneal space repair

The skin will be cut and subcutaneous tissue will be dissected. The abdominal external oblique aponeurosis will be dissected along the fibers. The abdominal internal oblique aponeurosis and transversus abdominis muscle will be bluntly separated to expose the transverse fascia. Dissociation of the hernia sac will be done as described above in the anterior transversalis fascia repair.

Transabdominal preperitoneal or totally extraperitoneal laparoscopic techniques were used to dissociate preperitoneal space, strip spermatic cord peritoneum, anatomize and expose the pubic pore. A polypropylene patch will be placed in the prevesical

For peer review only - http://bmjopen.bmj.com/site/about/guidelines.xhtml

space and Bogros' space to achieve repair of the pubic pore-containing area.

Concomitant treatment

Any medications, with the exception of inguinal hernia-specific treatments, administered during hospitalization will be recorded. Before starting the trial (i.e., at the first visit), detailed information will be recorded regarding concomitant diseases, combined medication and measures to be taken. At discharge, changes in medications and measures to be taken will be recorded. For every combination of medication and measures to be taken, a minimum of the following information will be recorded: drug name (generic preferred), dosage, start date, stop date or continuing use, and indications.

Study flowchart

Before surgery

Visit 1 (at admission, day 0)

· Sign informed consent

· Recheck inclusion/exclusion criteria

·Demographic data (sex, age, body height, body mass index) and medical insurance type

For peer review only - http://bmjopen.bmj.com/site/about/guidelines.xhtml

· Type of hernia (indirect or direct)

· Inguinal hernia classification as type I, II, III, or IV according to the adult inguinal hernia and femoral hernia classification (revised in 2003) developed by the Hernia and Abdominal Wall Surgery Group, Society of Surgery, Chinese Medical Association[19]

· ASA classification

· History of diseases and risk factors* (diabetes mellitus, cardiovascular disease, lung disease, peripheral vascular disease, dementia, hypertension, smoking history and alcohol use history) (*optional evaluation items)

·Disease onset and admission (interval from first appearance of the lump, main symptoms)

· Imaging examination (ultrasound, CT)

· Laboratory examination (routine blood testing, coagulation testing, testing of blood glucose, lipids and electrolytes, and hepatic and renal function)

· Vital signs (body temperature, pulse, respiration rate, blood pressure)

·VAS pain score

· Electrocardiography

· Concomitant treatment

The baseline information of patients with inguinal hernia included in this study is shown in Table 2.

For peer review only - http://bmjopen.bmj.com/site/about/guidelines.xhtml

Table 2 Baseline information of patients with inguinal hernia

Sex	Smoking history
Age	History of alcohol use
Body height	Disease attack and admission
Body mass index	Laboratory examination
Medical insurance type	Imaging examination
Type of hernia	Vital sign
Inguinal hernia classification	Visual Analogue Scale (VAS) pain score
Treatment time	Electrocardiography
American Society of Anesthesiologist	Concomitant therapy
Classification	
History of diseases	
Diabetes mellitus	
Cardiovascular disease	
Lung disease	
Peripheral vascular disease	
Dementia	
Hypertension	

For peer review only

Pre-discharge

Visit 2

· Vital signs (body temperature, pulse, respiration rate, blood pressure)

· Laboratory examination (routine blood testing, coagulation testing, testing of blood

For peer review only - http://bmjopen.bmj.com/site/about/guidelines.xhtml

glucose, lipids and electrolytes, and hepatic and renal function)

· Treatment regimen (inguinal hernia-specific treatment)

Surgical approach and patches (type and fixation method); anesthesia method; drug name (generic preferred), dosage, route, start date, length of drug use will be recorded.

· Medical costs

·Concomitant treatment

Inguinal hernia-specific treatment, maintenance therapy, and drugs used for concomitant treatment.

· Medical costs because of adverse events (AE)

Follow-up

Visit 3 (1 week after surgery via clinic visit or telephone follow-up)

·36-Item Short Form Health Survey (SF-36) score

· Medical costs

· Hernia recurrence (date and severity of recurrence).Recurrence will be diagnosed by the medical institutions, after being alerted by patients that symptoms are present.

· Presence or absence of hematoma and/or seroma.

· Wound infection (date and severity of wound infection).

For peer review only - http://bmjopen.bmj.com/site/about/guidelines.xhtml

Visit 4 (1 month after surgery via clinic visit or telephone follow-up)

·SF-36 score

· Medical costs

· Hernia recurrence (date and severity of recurrence). Recurrence will be diagnosed by the medical institutions, after being alerted by patients that symptoms are present.

· Presence or absence of hematoma and/or seroma

· Wound infection (date and severity of wound infection)

Visit 5 (3 months after surgery via clinic visit or telephone follow-up)

·SF-36 score

· Medical costs

· Hernia recurrence (date and severity of recurrence). Recurrence will be diagnosed by the medical institutions, after being alerted by patients that symptoms are present.

· Presence or absence of hematoma and/or seroma

· Wound infection (date and severity of wound infection)

Visit 6 (1 year after surgery via telephone follow-up)

· VAS pain score

·SF-36 score

For peer review only - http://bmjopen.bmj.com/site/about/guidelines.xhtml

· Medical costs· Hernia recurrence (date and severity of recurrence). Recurrence will be diagnosed by the medical institutions, after being alerted by patients that symptoms are present.

· Presence or absence of hematoma and/or seroma

· Wound infection (date and severity of wound infection)

Visit 7 (2 years after surgery via telephone follow-up)

· VAS pain score

·SF-36 score

· Medical costs

· Hernia recurrence (date and severity of recurrence). Recurrence will be diagnosed by the medical institutions, after being alerted by patients that symptoms are present.

· Presence or absence of hematoma and/or seroma

· Wound infection (date and severity of wound infection)

Outcome measures

·Primary outcome measure

The incidence of chronic inguinal pain at 1 and 2 years after surgery. To reduce the outcome bias of a single evaluation, chronic pain at 1 and 2 years after surgery will be

For peer review only - http://bmjopen.bmj.com/site/about/guidelines.xhtml

evaluated. According to International Association for the Study of Pain, VAS pain score >0 for over 3 successive months indicatates chronic inguinal pain[20].

·Secondary outcome measures

Postoperative complications including wound infection, rates of hematoma, seroma, hernia recurrence, intestinal obstruction, intestinal fistula, entocele, mesenteric thrombosis, femoral vein thrombosis, ischemic orchitis, painful ejaculation, varicocele and scrotal edema at postoperative 1 week, 1 and 3 months, and 1 and 2 years.

·Other outcome measures

Quality of life as evaluated by the SF-36[21].The SF-36 is a 36-item, patient-reported survey of patient health. It consists of eight scaled scores, including vitality, physical functioning, bodily pain, general health perceptions, physical functioning, emotional functioning, social functioning, and mental health. The score of each scale is summed and then standardized according to the formula: standardized score = (actual raw score- lowest possible raw score)/possible raw score range ×100. The total SF-36 score is the standardized score based on the sum of the eight scaled scores. A higher score indicates better quality of life.

The cost-utility analysis of therapeutic regimens involving different surgical approaches

For peer review only - http://bmjopen.bmj.com/site/about/guidelines.xhtml

will be analyzed. Medical costs consist of direct medical and non-medical costs. The direct medical costs include drug charges, inspection fees, laboratory fees, treatment fees, nursing fees, and bed charges. The direct medical costs during hospitalization will be calculated according to the hospital information system. Direct medical costs during the follow-up period will be reported by patients and/or their relatives. Direct non-medical costs include payments for transportation to receive medical care, cost of nutritional supplementation, and the costs for accompanying family members during the treatment period.

All outcome evaluations will be independently performed by an experienced assessor blinded to the treatment regimens. The schedule of outcome measurement assessment is shown in Table 3.

Table 3 Timing of outcome measurement assessment

	Before surgery		During surgery		Follow up		
	Visit 1 (at admission, day 0)	Visit 2 (at discharge)	Visit 3 (1 week after surgery)	Visit 4 (1 month after surgery)	Visit 5 (3 months after surgery)	Visit 6 (1 year after surgery)	Visit 7 (3 years after surgery)
Signed informed consent	X						
Inclusion/exclusi	X						

For peer review only - http://bmjopen.bmj.com/site/about/guidelines.xhtml

on criteria							
Demographic data	X						
Medical insurance type	X						
Delayed visit and admission	X						
Previous history of diseases	X						
Previous history of drug	X						
Risk factors	X						
Disease attack and admission [a]	X						
Type of hernia (indirect hernia, direct hernia)	X						
Vital sign[b]	X						
Laboratory examination [c]	X	X					
Imaging examination [d]	X						
Electrocardiography	X						
Treatment		X					

For peer review only

For peer review only - http://bmjopen.bmj.com/site/about/guidelines.xhtml

regimen [e]							
VAS score [f]						X	X
SF-36 score			X	X	X	X	X
Medical cost		X	X	X	X	X	X
Concomitant treatment	X	X					
Adverse events<br>Wound infection<br>Hematoma<br>Seroma<br>Hernia recurrence		X	X	X	X	X	X

[a] indicates the interval from the first appearance of the lump or main symptoms; [b] indicates body temperature, pulse, respiration rate, and blood pressure; [c] indicates routine blood testing, coagulation testing, testing of blood glucose, lipids, and electrolytes, and hepatic and renal function; [d] indicates ultrasound, CT examination; [e] indicates inguinal hernia-specific treatment; f indicates the VAS pain score.

Adverse events and serious adverse events (SAE)

According to the study protocol and clinical judgment, AE/SAE occurring after herniorrhaphy will be reported to the Department of General Surgery, the Fourth Affiliated Hospital of China Medical University.

For peer review only

For peer review only - http://bmjopen.bmj.com/site/about/guidelines.xhtml

AE refer to any adverse medical events occurring after herniorrhaphy in patients or clinical subjects. SAE refer to any adverse postoperative medical events involving one or more of the following criteria:

○ Death, irrespective of the cause

○ Life-threatening event

○ Severe or permanent disability or organ dysfunction

○ Hemorrhage

○ Malformation, birth defect

○Hospitalization or extended hospital stay

○ Re-admission to hospital

○ Recurrence (symptomatic) of inguinal hernia

Causal relationship between surgery and adverse events

The causal relationship between the drugs used and AE will be evaluated by the researchers as: certainly relevant, probably relevant, likely relevant, unlikely relevant and irrelevant (Table 4).

Table 4 Causal relationship between surgery and adverse events

	Certainly relevant	Probably relevant	Likely relevant	Unlikely relevant	Irrelevant

For peer review only - http://bmjopen.bmj.com/site/about/guidelines.xhtml

Adverse events are obviously caused by external factors	-	-	-	-	+
Adverse events are correlated with surgical treatment at rational time	+	+	+	-	-
Adverse events are correlated with patient diseases	-	-	+	+	+
Adverse events are correlated with suspected postoperative response patterns	+	+/-	+	-	-
After relief of related surgical factors, adverse events alleviate or disappear	+	+	-	-	-
After surgery-related factors worsen, adverse events recur	+	+	-	-	-

Note: To minimize the surgical risk and meet the requirements of laws and regulations, the sponsor will manage the correlations as follows: "Irrelevant" belongs to the

For peer review only - http://bmjopen.bmj.com/site/about/guidelines.xhtml

irrelevant category, and “certainly relevant”, “probably relevant”, “likely relevant” and “unlikely relevant” belong to the relevant category.

Evaluation criteria for the severity of adverse events

Mild: The patient is aware of symptoms, but symptoms can be tolerated. Symptoms are causing mild discomfort, but not interfering with daily activities.

Moderate: Not affecting daily activities.

Severe: Very painful, causing significant functional impairment or loss of self-care ability, and prohibiting the patient from carrying out daily activities.

The researcher will evaluate the severity of AE according to clinical indices such as laboratory and inspection outcomes, not just based on the subject’s direct feelings.

Adverse event reporting

Reporting time limit for AE will be within 24 hours of onset. In the clinical research period (from the time of signing informed consent to 1–2 years postoperatively), any AE occurring in any patient who received either surgical approach will be properly treated. According to the requirements of this study protocol and clinical evaluation, researchers will fill in the table of *Adverse Events After Herniorrhaphy* and submit this within 24 hours to the Clinical Research Center of Abdominal Wall Hernia, the Fourth

For peer review only - http://bmjopen.bmj.com/site/about/guidelines.xhtml

Affiliated Hospital of China Medical University, China.

Serious adverse event emergency reporting

Reporting time limit for SAE will be within 24 hours of onset. Any SAE will be continuously monitored and reported until it is healed, stabilizes or recovers to near baseline conditions, irrespective of whether patients have terminated or completed treatment. Any follow-up information regarding SAE will be reported within 24 hours.

Patient completion/withdrawal from clinical study

Patients for whom the whole clinical data of at least 1 year are collected will be considered as complete cases. Patients with any one or more of the following criteria will be considered withdrawn from the study: mistakenly recruited, withdrawal of informed consent, upon the request of the sponsor for safety reasons or patient conflicts, or lost to follow-up. The date and reasons for withdrawal will be recorded on the eCRF. After termination of the study, the data collected at the last visit will be evaluated, except for data from those lost to follow-up.

Statistical analysis

Statistical analysis will be performed by a statician using SPSS 19.0 software (SPSS,

For peer review only - http://bmjopen.bmj.com/site/about/guidelines.xhtml

Chicago, IL, USA). Continous variables will be statistically described using the mean, standard deviation, median, minimum and maximum. The categorical variables will be expressed using numbers and percentages.

The analysis will be performed on the basis of the intention-to-treat principle. Descriptive statistics of baseline information will be performed. The chi-squared test or Fisher's exact test will be used for analysis of categorical variables, such as the incidence of postoperative chronic pain (primary outcome measure) and the incidence of postoperative complications (secondary outcome measure) between groups. Independent sample t-test or Mann-Whitney U test will be used for comparisons of continous variables, such as SF-36 score, between groups. Cost-utility analysis will be used for economic evaluation, and sensitivity analysis of cost and utility will be performed.

Interim analysis

When an adequate number of patients are enrolled and followed-up, interim analysis will be performed. When data are included for the full analysis set and recorded in the database, the first interim analysis during the management period will be performed to check whether the core data collected are suitable for preliminary significant data analysis. According to research progression, subsequent interim analysis of all data

For peer review only - http://bmjopen.bmj.com/site/about/guidelines.xhtml

included in the database will then be designated. After acquiring approval from the Department of General Surgery and Scientific Construction Committee, the Fourth Affiliated Hospital of China Medical University, the data collected in the Department of General Surgery, the Fourth Affiliated Hospital of China Medical University are likely to be analyzed together with the data collected from the other research centers. In accordance with applicable laws and regulations, the information on the subjects in the study will be kept confidential. The data for interim analysis will be precisely described in a statistical analysis plan. The interim analysis results will be submitted to an independent Data Monitoring Committee in the form of a statistical analysis report and as slides.

Sample size

According to previous reports[22,23], we hypothesized that the incidence of chronic inguinal pain after anterior transversalis fascia repair and preperitoneal space approach was 10% and 3.4%, respectively. Taking $\alpha = 0.05$ and power = 90%, the final effective sample size of $n = 600$ was calculated. Assuming a patient loss rate of 20%, we require 720 patients.

Ethics and dissemination

For peer review only - http://bmjopen.bmj.com/site/about/guidelines.xhtml

For peer review only

Ethical approval

Before study commencement, the following files will be provided to the Independent ethics committee (IEC):

○ Final draft of study protocol (and supplements)

○ Sponsor-approved informed consent and other documents provided to the subjects (such as participation card and diary card)

○ Materials assisting patients to be included

○ Materials regarding study-related injury compensations or rewards for patient participation in the study

○ Researcher résumé or equivalent (unless the IEC states that this is not needed)

○ Sponsor name, funds, potential competing interests, and information that affects patient participation in the study

○Any other documents required by the IEC

Trials cannot be started until the IEC completely approves the study protocol, informed consent, materials assisting patients to be included, and compensation measures for the patients, and the sponsor receives a copy of the IEC approval document (Additional file 3). The IEC approval document should include the trial title (registration number), name of the study file (including edition code) and date of approval.

For peer review only - http://bmjopen.bmj.com/site/about/guidelines.xhtml

During the study period, it is likely that researchers will submit the following files to the IEC for approval at appropriate time-points:

○ Supplement of study protocol

○ Informed consent forms and documents regarding rewards for patient participation in the study

○ New information that is likely to negatively affect participant safety and study progression

○ Files regarding bias and alterations of the study protocol made to avoid immediate injury to patients

○ Reports regarding dead patients

○ Notification of change of project manager

○ Other requirements of the IEC

If the supplemented study protocol increases the risk to patients, the supplemented study protocol and corresponding modified informed consent form will be submitted to the IEC for consideration. The supplemented study protocol will not be performed until approval from the IEC is obtained. The major study protocol was approved by the IEC, the Fourth Affiliated Hospital of China Medical University (approval number 2015-027)

For peer review only - http://bmjopen.bmj.com/site/about/guidelines.xhtml

on November 27, 2015.The study protocol should be reviewed by the IEC at least once every year, and the reviewed suggestion will be recorded on paper. At the end of the study, the researchers should inform the IEC of its completion.

We began recruitment in February 2017 and expect to have completed recruitment by December 2019 and completed data collection by June 2020. The final results of the trial will be published in international peer-reviewed journals.

Informed consent to participate

Each patient (or his/her legal representative) will provide signed and dated informed consent before surgery after fully understanding the objective and contents of the study (Additional file 4). The researcher or his/her authorized staff members will fully explain the objective, methods, possible benefits, potential risks and any possible discomforts of the study to the potential patients before inclusion. Participants will be informed that participation in the study is voluntary and that they can withdraw from the study at any time. The participants will know that their identifying information will be recorded for long-term follow-up, and will be read by personnel from the related institutions and the sponsor within the permit of relevant laws and regulations. The right to privacy of the participant will be protected.

For peer review only - http://bmjopen.bmj.com/site/about/guidelines.xhtml

Confidentiality

○ Only data required to investigate the effectiveness and safety of herniorrhaphy will be collected and analyzed.

○ Data collection and use will not be disclosed to any non-authorized persons, and will be performed in accordance with the laws and regulations regarding protection of the participant's privacy.

○ The process of data collection will be fair and lawful.

○ The purpose of data collection will be specific, identified and legitimate, and the collected data will not be used for other unrelated objectives.

○ The data collected will be adequate, related and not redundant relative to the study objective.

○ The data collected will be accurate and updated when necessary.

○ Before collection of personal data, researchers will obtain participant consent, which should include lay emphasis on the transfer of data to other institutional entities and countries.

○ The participants have the right to obtain their data and can request to modify mistaken or incomplete data.

○ During the study period, participant's personal information will not be obtained or disclosed to non-authorized persons, and will not be illegally destroyed, lost or altered

For peer review only - http://bmjopen.bmj.com/site/about/guidelines.xhtml

unexpectedly. During the entire study period, the sponsors who have the right to read the participant's personal information will keep the data confidential.

Dissemination

Any unpublished information provided by the sponsors, and all unpublished data relating to this study will be kept confidential and will be owned by the sponsor. This information or data will be not be used for other purposes unless written approval is acquired from the sponsor. The clinical researchers will be informed that study results will be used for further study. Therefore, the study results may be provided to other clinical researchers or related administrative departments. The study results will be disclosed in the form of a Clinical Study Report, including data collected from any research center involved. If clinical researchers publish the study outcomes, they will provide the original manuscript to the sponsor for online review 60 days before submission or presentation. A summary, posters or other promotional materials will be created to facilitate the review. The sponsor will discuss scientific and regulatory compliance issues with clinical researchers. The sponsor will not mandatorily require the clinical researchers to modify the scientific contents, and has no right to hide information. The clinical researchers should consider the integrity of this multi-center study. The data from one research center can be published only under the following

For peer review only - http://bmjopen.bmj.com/site/about/guidelines.xhtml

circumstances: the articles involving the outcomes from all research centers have been published; study in all research centers has been accomplished, abandoned or terminated for 12 months; the sponsor has stated that they will not publish the study outcomes from multiple research centers. Assignment of the author listings in articles related to this study will be performed based on the author's contribution guidelines, such as the guidelines of Uniform Requirements for Manuscripts Submitted to medical journals.

Data management

Protocol modification

Study protocol modification will be signed, dated, and published by the Department of General Surgery, the Fourth Affiliated Hospital of China Medical University. The study protocol will not be put into clinical practice until IEC approval is received, unless this is necessary to avoid risk to participants, or to modify the study protocol regarding logistics and administration (for example, typographical errors and contradictions).

The study protocol should not be deviated from during clinical practice. When deviation exists, corresponding management will be performed. The causes and deviated contents will be recorded in the eCRF and original medical case notes. The study protocol deviation table and eCRF will be preserved in the research center and sponsor

For peer review only - http://bmjopen.bmj.com/site/about/guidelines.xhtml

institute.

Participant identity registration and screening records

Participants must agree to fill in identification registration to enable individual identification of each participant. The monitor will recheck the integrity of this registration. The participant identity registration form will be confidential, and will be preserved in the research center. To ensure confidentiality, duplication of participant identity registration will be not permitted. All reports and letters relating to this study will be tagged with the relevant acronyms and serial number. The participant screening record form will be completed by doctors. The doctors will determine whether participants are eligible for admission to this study.

Electronic case report form

In this study, the EDC will be used for data collection and management. All data relating to this study will be recorded on the eCRF provided by the sponsor. The researchers will fill in the eCRF after each participant visit, unless some clinical results cannot be acquired immediately. This ensures that the information recorded on the eCRF reflects the participant's latest outcome. Data accuracy will be performed by the researcher. Data recording, alteration and substitution will be performed by

For peer review only - http://bmjopen.bmj.com/site/about/guidelines.xhtml

researchers or other authorized persons. All data will be inputted into the EDC, and data queries will be made by researchers online via the EDC. The final data will not be altered, and will be password-protected.

Data quality assurance

To ensure data accuracy and reliability, eligible researchers and appropriate research centers will be selected before study commencement. The monitor in the co-sponsor research center will monitor the study progression periodically. The co-sponsor will advise the researchers how to fill in the eCRF. The monitor in the co-sponsor research center will visit the EDC to check the integrity and accuracy of the eCRF. Data recorded in the eCRF that is inconsistent with original data recorded will be altered by researchers or authorized persons.

Auditing

Regular on-site inspection visits will be made by the co-sponsors. The co-sponsors monitor will date the inspection on an inspection form, which will then be preserved in the research center. After study commencement, the first on-site inspection visit will be performed as soon as possible after participant recruitment. During on-site inspection, the monitor will check the consistency of data recorded in the eCRF with original data

For peer review only - http://bmjopen.bmj.com/site/about/guidelines.xhtml

For peer review only

recorded in medical notes from the research center. The nature and preservation place of original data documents will be confirmed to enable clinical researchers to know the source of all original data required in the eCRF, thus the monitor of the co-sponsor can recheck these data.

If original data are electronically preserved, the monitor of the co-sponsor will discuss the recheck method with clinical researchers. The original data document will include participant identity, eligibility for inclusion, informed consent, dates of visits, execution of study protocol, curative effects, safety index, AE reporting and follow-up, medication, and date of study completion.

The monitor of the co-sponsor will discuss the detailed requirements for original data recording with clinical researchers. To recheck whether the data recorded in eCRF is consistent with original data, the monitor of the co-sponsor will be provided with the required original data. The monitor will discuss any problems found during rechecking of data consistency with clinical researchers. The clinical researchers will regularly discuss the information feedback.

Study completion/termination

Study completion

When the last visit of the last participant is completed, the research center will inform

For peer review only - http://bmjopen.bmj.com/site/about/guidelines.xhtml

the sponsor, and study completion will be designated. The sponsor will inform all research centers of the time of study completion. Further research after this time must be approved by the sponsor and can then be performed without protocol supplements.

Study termination

The sponsor will have the right to terminate the study at any research center at any time possibly because of, but not limited to, the following criteria:

○ The number of patients recruited reaches the predetermined requirements

○ Research cannot abide by the study protocol or GCP guidelines

○Insufficient numbers of participants are recruited

Audit and inspection

A representative of the department of clinical quality assurance of the co-sponsor may visit any of the research centers to determine whether the study protocol follows the laws and regulations. All study records, including original medical notes, will be disclosed to the representative. However, the privacy of the subject will be respected. The research center will be informed about this visit in advance to allow sufficient time for appropriate preparation.

For peer review only

DISCUSSION

Significance of this study

This study will be the first large sample (720 patients), multi-center, randomized, controlled, clinical trial to investigate the effectiveness of inguinal hernia repair via the anterior transversalis fascia approach versus the preperitoneal approach in patients from northern China. In this study, postoperative complications will be used as the primary outcome measure, and patient quality of life and cost-utility analysis will be the secondary outcome measures.

However, this study is also limited: (1) Although all surgeons involved in the study will receive training of standard surgical procedure, confounding variables including surgeon's clinical experiences and surgical conditions should be considered to reduce the bias in the estimate of the study outcomes. (2) The time taken for studying postoperative recurrence of inguinal hernia is limited, which likely influences the judgment of long-term recurrence of inguinal hernia.

Contribution to future studies

Based on study results, we aim to effectively reduce physical and psychological pain, ensure high quality medical care (including safety), and achieve the best rehabilitation

For peer review only - http://bmjopen.bmj.com/site/about/guidelines.xhtml

in the treatment of inguinal hernia. We aim to determine how to reduce medical resources (including shortening treatment time and reducing labor service strength) and medical costs, and improve the efficiency of medical work and other issues. This study will provide important clinical guidance as to the method of inguinal hernia repair that is most suitable for the anatomic characteristics of patients in northern China and adaptive to the regional economic situation.

Trial status

Recruitment of patients is ongoing at the time of submission.

Abbreviations

AE	adverse event
eCRF	electronic case report form
EDC	electronic data capture
IEC	independent ethics committee
SAE	serious adverse event
CUA	Cost utility analysis

For peer review only

For peer review only - http://bmjopen.bmj.com/site/about/guidelines.xhtml

References

1. Beddy P, Ridgway PF, Geoghegan T, *et al*. Inguinal hernia repair protects testicular function: a prospective study of open and laparoscopic herniorraphy. *J Am Coll Surg*. 2006;203:17-23.

2. Palermo M, Acquafresca PA, Bruno M, Tarsitano F. Hernioplasty with and without mesh: analysis of the immediate complications in a randomized controlled clinical trial. *Arq Bras Cir Dig*. 2015;28:157-160.

3. Chatzimavroudis G, Papaziogas B, Koutelidakis I, *et al.* Lichtenstein technique for inguinal hernia repair using polypropylene mesh fixed with sutures vs. self-fixating polypropylene mesh: a prospective randomized comparative study. *Hernia*. 2014;18:193-198.

4. Bittner R, Arregui ME, Bisgaard T, *et al*. Guidelines for laparoscopic (TAPP) and endoscopic (TEP) treatment of inguinal hernia [International Endohernia Society (IEHS)]. *Surg Endosc*. 2011;25:2773-843.

5. Carter J, Duh QY. Laparoscopic repair of inguinal hernias. *World J Surg*. 2011;35:1519-25.

6. Greater China Endo Hernia Institute; Laparoscopic and Endoscopic Group, Society of Surgery, Chinese Medical Association; Hernia and Abdominal Wall Surgery Group, Society of Surgery, Chinese Medical Association.Guidelines for

For peer review only - http://bmjopen.bmj.com/site/about/guidelines.xhtml

Standardization of Laparoscopic Surgery for Inguinal Hernia.*Chin J Hernia Abdominal Wall Surg* (Electronic). 2013;7:505-512..

7. Karateke F, Ozyazici S, Menekse E, *et al*. ULTRAPRO Hernia System versus lichtenstein repair in treatment of primary inguinal hernias: a prospective randomized controlled study. *Int Surg*. 2014;99:391-7.
8. Kugel RD. The Kugel repair for groin hernias. *Surg Clin North Am*. 2003;83:1119-39.
9. Zhu X, Cao H, Ma Y, *et al*. Totally extraperitoneal laparoscopic hernioplasty versus open extraperitoneal approach for inguinal hernia repair: a meta-analysis of outcomes of our current knowledge. *Surgeon*. 2014;12(2):94-105.
10. Pisanu A, Podda M, Saba A, Porceddu G, Uccheddu A. Meta-analysis and review of prospective randomized trials comparing laparoscopic and Lichtenstein techniques in recurrent inguinal hernia repair. *Hernia*. 2015;19:355-366.
11. Akhtar MS, Ali WM, Khan TA, *et al*. Laparoscopic transabdominal preperitoneal versus open mesh lichtenstein repair of inguinal hernia: a comparative analysis. *Int J Med Res Rev*. 2016;4:1119-1206.
12. Sarhan AE, Sherif T, El-Kenany Y. Open preperitoneal mesh repair versus laparoscopic transabdominal preperitoneal repair of groin hernia under spinal

For peer review only - http://bmjopen.bmj.com/site/about/guidelines.xhtml

For peer review only

anesthesia: results of a prospective randomized multicenter trial. *Egypt J Surg*. 2016;35:132-139

13. Kargar S, Shiryazdi SM, Zare M, Mirshamsi MH, Ahmadi S, Neamatzadeh H. Comparison of postoperative short-term complications after laparoscopic transabdominal preperitoneal (TAPP) versus Lichtenstein tension free inguinal hernia repair: a randomized trial study. *Minerva Chir*. 2015;70:83-89.
14. Salma U, Ahmed I, Ishtiaq S. A comparison of post operative pain and hospital stay between Lichtenstein's repair and Laparoscopic Transabdominal Preperitoneal (TAPP) repair of inguinal hernia: A randomized controlled trial. *Pak J Med Sci*. 2015;31:1062-1066.
15. Bahram MAL. Early and late outcomes of trans-abdominal pre-peritoneal and Lichtenstein repair for inguinal hernia, a comparative study. *Int Surg J*. 2017;4:459-464.
16. CONSORT Group (2008) CONSORT: Consolidated Standards of Reporting Trials.
17. Abdominal Wall Surgery Group, Society of Surgery, Chinese Medical Association. Guidelines for the Diagnosis and Treatment of Inguinal Hernia in Adults (2014 Edition).*Chin J Prac Surg*. 2014;52:484-486.
18. Miserez M, Peeters E, Aufenacker T, *et al*. Update with level 1 studies of the European Hernia Society guidelines on the treatment of inguinal hernia in adult

For peer review only - http://bmjopen.bmj.com/site/about/guidelines.xhtml

patients. *Hernia*. 2014;18:151-163.

19. The Chinese Medical Association Society of Surgery and Abdominal Wall Hernia Surgery Group. Adult inguinal hernia and femoral hernia surgery methods (revised). *Chin J Gen Surg*. 2004;19:126.
20. Nikkolo C, Lepner U. Chronic pain after open inguinal hernia repair. *Postgrad Med*. 2016;128:69-75.
21. Lins L, Carvalho FM. SF-36 total score as a single measure of health-related quality of life: Scoping review. *SAGE Open Med*. 2016;4:2050312116671725.
22. Yin WT，Li P，Zhao WS，Li RH. The Analysis and Influence of Anterior Preperitoneal Space on Chronic Pain after Herniorrhaphy. *J Kunming Med Univ*. 2011;(5):90-92.
23. Zhang J, Bai J, Li Q, Li ZH, Li L. Anterior versus posterior tension-free repair of adult inguinal hernia. *Shaanxi Med J*. 2011;40:331-332.

For peer review only - http://bmjopen.bmj.com/site/about/guidelines.xhtml

Authors' contributions

HYL, FQY, SJZ, YQW, WDA, ZSD, HYJ, FRW and SFQ conceived the study and participated in its design and coordination. LY drafted the manuscript. SBW and QF participated in the design of the study and performed the statistical analysis. DWZ and DYY participated in the study design and coordination and helped draft the manuscript. HWL participated in the design of the study and wrote the protocol for the analysis. All authors read, revised and approved the final manuscript.

Funding Statement

This research was funded by National Natural Science Foundation NO.81472302.

Competing interests Statement

The authors declare that they have no competing interests.

Data sharing statement

The results of this study will be disseminated via peer-reviewed publications and conference presentations. No data are available at the moment.

For peer review only

For peer review only - http://bmjopen.bmj.com/site/about/guidelines.xhtml

Figure 1 Flow chart of study protocol.

VAS: Visual Analogue Scale; SF-36: 36-Item Short Form Health Survey.

Tables

Table 1 Randomized controlled trials (RCTs) regarding inguinal hernia repair approaches

Table 2 Baseline information of patients with inguinal hernia

Table 3 Timing of outcome measurement assessment

Table 4 Causal relationship between surgery and adverse events

Additional file 1: Trial committee organization and contributions and role. Description of data: trial committee organization and contributions and role in accordance with SPIRIT (Standard Protocol Items: Recommendations for Interventional Trials) 2013 Checklist.

Additional file 2: SPIRIT Checklist

Additional file 3: List of ethical approval documents

Additional file 4: Informed Consent to participate

For peer review only - http://bmjopen.bmj.com/site/about/guidelines.xhtml

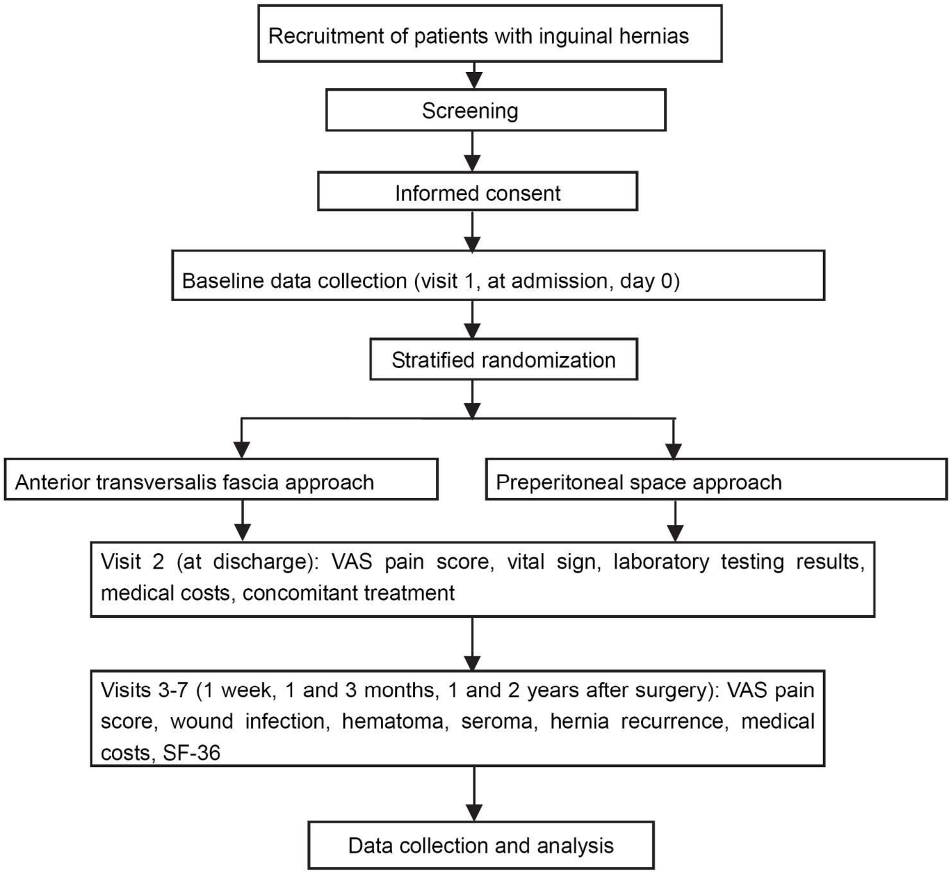

Flow chart of study protocol

184x169mm (300 x 300 DPI)

only

Additional file 1: Trial committee organization and contributions and role.

Principal investigator (PI) and research physicians at main study site

Organization: the Fourth Affiliated Hospital of China Medical University

Contributions and role: Principal investigator, original study design: Hang-yu Li (Email:li_hangyu@126.com)

Organization of Steering committee and member appointment: Qing Fan, De-wei Zhang, Da-ye Yang, Hong-wu Li, Shi-bo Wei, Liang Yang, Hang-yu Li, Fu-quan Yang, Shao-jun Zhang, Yao-qiang Wu, Wei-de An, Zhong-shu Dai, Hui-yong Jiang, Fu-rong Wang, Shi-feng Qiao

Communication and exchange of opinion with PI at each site

Preparation of IRB documents and CRF

Trial management (randomized allocation management, AE data collection at each site, participant enrollment supervision, study site inspection and visits, budget allocation and management)

Cooperation with CRO in data collection, quality control, monitoring, and analysis

Steering committee (SC)

Organization and role: All authors of this manuscript

Contributions: Protocol revision and decision on final protocol

Organization of Trial Management Committee and member appointment

Designation of participant recruitment study sites

Inspection of study progress, and decision on protocol revision, if needed

Determination of study result publication timing and method

Decision on authorship in accordance with Authorship eligibility guidelines

Trial Management Committee

Organization: PI and investigators at each clinical trial participant enrollment site

Fu-quan Yang, Shengjing Hospital of China Medical University

Shao-jun Zhang, Fengtian Hospital of Shenyang Medical College

Yao-qiang Wu, The First Hospital of Dandong City

Wei-de An, The First Affiliated Hospital of Dalian Medical University

Zhong-shu Dai, General Hospital of Benxi Steel and Iron (Group) Co., LTD, Fifth Clinical College of China Medical University

Hui-yong Jiang, General Hospital of Shenyang Military Area

Fu-rong Wang, The 202nd Hospital of PLA

Shi-feng Qiao, The First Affiliated Hospital of Liaoning Medical University

Hang-yu Li*, The Fourth Affiliated Hospital of China Medical University

Organization and role: Submission and obtaining study protocol approval from relevant IRB of each study site

Clinical trial execution following protocol (e.g. participant recruitment, enrollment, data collection, CRF

For peer review only

1 entry)

2 Collection and report of AEs

For peer review only

SPIRIT

STANDARD PROTOCOL ITEMS: RECOMMENDATIONS FOR INTERVENTIONAL TRIALS

SPIRIT 2013 Checklist: Recommended items to address in a clinical trial protocol and related documents*

Section/item	Item No	Description	Addressed on page number
**Administrative information**			
Title	1	Descriptive title identifying the study design, population, interventions, and, if applicable, trial acronym	p. 1
Trial registration	2a	Trial identifier and registry name. If not yet registered, name of intended registry	p. 5
	2b	All items from the World Health Organization Trial Registration Data Set	N/A
Protocol version	3	Date and version identifier	p. 41
Funding	4	Sources and types of financial, material, and other support	p. 50
Roles and responsibilities	5a	Names, affiliations, and roles of protocol contributors	p. 50
	5b	Name and contact information for the trial sponsor	p. 50
	5c	Role of study sponsor and funders, if any, in study design; collection, management, analysis, and interpretation of data; writing of the report; and the decision to submit the report for publication, including whether they will have ultimate authority over any of these activities	p. 50
	5d	Composition, roles, and responsibilities of the coordinating centre, steering committee, endpoint adjudication committee, data management team, and other individuals or groups overseeing the trial, if applicable (see Item 21a for data monitoring committee)	Additional file 2
**Introduction**			

For peer review only

Background and rationale	6a	Description of research question and justification for undertaking the trial, including summary of relevant studies (published and unpublished) examining benefits and harms for each intervention	p. 7
	6b	Explanation for choice of comparators	p. 11
Objectives	7	Specific objectives or hypotheses	p. 11
Trial design	8	Description of trial design including type of trial (eg, parallel group, crossover, factorial, single group), allocation ratio, and framework (eg, superiority, equivalence, noninferiority, exploratory)	p. 11-13
**Methods: Participants, interventions, and outcomes**			
Study setting	9	Description of study settings (eg, community clinic, academic hospital) and list of countries where data will be collected. Reference to where list of study sites can be obtained	p. 12-13
Eligibility criteria	10	Inclusion and exclusion criteria for participants. If applicable, eligibility criteria for study centres and individuals who will perform the interventions (eg, surgeons, psychotherapists)	p. 14
Interventions	11a	Interventions for each group with sufficient detail to allow replication, including how and when they will be administered	p. 15
	11b	Criteria for discontinuing or modifying allocated interventions for a given trial participant (eg, drug dose change in response to harms, participant request, or improving/worsening disease)	p. 15-16
	11c	Strategies to improve adherence to intervention protocols, and any procedures for monitoring adherence (eg, drug tablet return, laboratory tests)	p. 15
	11d	Relevant concomitant care and interventions that are permitted or prohibited during the trial	p. 17-18
Outcomes	12	Primary, secondary, and other outcomes, including the specific measurement variable (eg, systolic blood pressure), analysis metric (eg, change from baseline, final value, time to event), method of aggregation (eg, median, proportion), and time point for each outcome. Explanation of the clinical relevance of chosen efficacy and harm outcomes is strongly recommended	p. 23-25
Participant timeline	13	Time schedule of enrolment, interventions (including any run-ins and washouts), assessments, and visits for participants. A schematic diagram is highly recommended (see Figure)	p. 18-23, Figure 1

For peer review only - http://bmjopen.bmj.com/site/about/guidelines.xhtml

Sample size	14	Estimated number of participants needed to achieve study objectives and how it was determined, including clinical and statistical assumptions supporting any sample size calculations	p. 33
Recruitment	15	Strategies for achieving adequate participant enrolment to reach target sample size	p. 13
**Methods: Assignment of interventions (for controlled trials)**			
Allocation:			
Sequence generation	16a	Method of generating the allocation sequence (eg, computer-generated random numbers), and list of any factors for stratification. To reduce predictability of a random sequence, details of any planned restriction (eg, blocking) should be provided in a separate document that is unavailable to those who enrol participants or assign interventions	p. 15
Allocation concealment mechanism	16b	Mechanism of implementing the allocation sequence (eg, central telephone; sequentially numbered, opaque, sealed envelopes), describing any steps to conceal the sequence until interventions are assigned	p. 15
Implementation	16c	Who will generate the allocation sequence, who will enrol participants, and who will assign participants to interventions	p.15
Blinding (masking)	17a	Who will be blinded after assignment to interventions (eg, trial participants, care providers, outcome assessors, data analysts), and how	p. 15
	17b	If blinded, circumstances under which unblinding is permissible, and procedure for revealing a participant's allocated intervention during the trial	p. 15
**Methods: Data collection, management, and analysis**			
Data collection methods	18a	Plans for assessment and collection of outcome, baseline, and other trial data, including any related processes to promote data quality (eg, duplicate measurements, training of assessors) and a description of study instruments (eg, questionnaires, laboratory tests) along with their reliability and validity, if known. Reference to where data collection forms can be found, if not in the protocol	p. 39-42
	18b	Plans to promote participant retention and complete follow-up, including list of any outcome data to be collected for participants who discontinue or deviate from intervention protocols	p. 39

For peer review only - http://bmjopen.bmj.com/site/about/guidelines.xhtml

Data management	19	Plans for data entry, coding, security, and storage, including any related processes to promote data quality (eg, double data entry; range checks for data values). Reference to where details of data management procedures can be found, if not in the protocol	p. 41
Statistical methods	20a	Statistical methods for analysing primary and secondary outcomes. Reference to where other details of the statistical analysis plan can be found, if not in the protocol	p. 31-32
	20b	Methods for any additional analyses (eg, subgroup and adjusted analyses)	p. 32-33
	20c	Definition of analysis population relating to protocol non-adherence (eg, as randomised analysis), and any statistical methods to handle missing data (eg, multiple imputation)	p. 32-33
**Methods: Monitoring**			
Data monitoring	21a	Composition of data monitoring committee (DMC); summary of its role and reporting structure; statement of whether it is independent from the sponsor and competing interests; and reference to where further details about its charter can be found, if not in the protocol. Alternatively, an explanation of why a DMC is not needed	p. 41-42
	21b	Description of any interim analyses and stopping guidelines, including who will have access to these interim results and make the final decision to terminate the trial	p. 42
Harms	22	Plans for collecting, assessing, reporting, and managing solicited and spontaneously reported adverse events and other unintended effects of trial interventions or trial conduct	p. 41
Auditing	23	Frequency and procedures for auditing trial conduct, if any, and whether the process will be independent from investigators and the sponsor	p. 41
**Ethics and dissemination**			
Research ethics approval	24	Plans for seeking research ethics committee/institutional review board (REC/IRB) approval	p. 33-36 Additional file 4
Protocol amendments	25	Plans for communicating important protocol modifications (eg, changes to eligibility criteria, outcomes, analyses) to relevant parties (eg, investigators, REC/IRBs, trial participants, trial registries, journals, regulators)	p. 36-37

For peer review only - http://bmjopen.bmj.com/site/about/guidelines.xhtml

Consent or assent	26a	Who will obtain informed consent or assent from potential trial participants or authorised surrogates, and how (see Item 32)	p. 36
	26b	Additional consent provisions for collection and use of participant data and biological specimens in ancillary studies, if applicable	N/A
Confidentiality	27	How personal information about potential and enrolled participants will be collected, shared, and maintained in order to protect confidentiality before, during, and after the trial	p. 36-37
Declaration of interests	28	Financial and other competing interests for principal investigators for the overall trial and each study site	p. 50
Access to data	29	Statement of who will have access to the final trial dataset, and disclosure of contractual agreements that limit such access for investigators	p. 50
Ancillary and post-trial care	30	Provisions, if any, for ancillary and post-trial care, and for compensation to those who suffer harm from trial participation	p. 30
Dissemination policy	31a	Plans for investigators and sponsor to communicate trial results to participants, healthcare professionals, the public, and other relevant groups (eg, via publication, reporting in results databases, or other data sharing arrangements), including any publication restrictions	p. 41-42
	31b	Authorship eligibility guidelines and any intended use of professional writers	N/A
	31c	Plans, if any, for granting public access to the full protocol, participant-level dataset, and statistical code	N/A
**Appendices**			
Informed consent materials	32	Model consent form and other related documentation given to participants and authorised surrogates	Additional file 3
Biological specimens	33	Plans for collection, laboratory evaluation, and storage of biological specimens for genetic or molecular analysis in the current trial and for future use in ancillary studies, if applicable	p. 11-13

*It is strongly recommended that this checklist be read in conjunction with the SPIRIT 2013 Explanation & Elaboration for important clarification on the items. Amendments to the protocol should be tracked and dated. The SPIRIT checklist is copyrighted by the SPIRIT Group under the Creative Commons "Attribution-NonCommercial-NoDerivs 3.0 Unported" license.

For peer review only

Additional file 4: List of ethical approval documents

Center	Name of ethical bodies	Ethical approval reference number
Department of General Surgery, the Fourth Affiliated Hospital of China Medical University	The Ethics Committee of the Fourth Affiliated Hospital of China Medical University	2015-027
Department of General Surgery, Branch 3, First Hospital of Dalian Medical University	The Ethics Committee of First Hospital of Dalian Medical University	2016-034
Department of General Surgery, the 202 Hospital of Chinese PLA	The Ethics Committee of the 202 Hospital of Chinese PLA	2015-033
Ward of Hernia, Department of General and Gastrointestinal Surgery, First Affiliated Hospital of Liaoning Medical University	The Ethics Committee of First Affiliated Hospital of Liaoning Medical University	2016-022
Department of General Surgery, General Hospital of Shenyang Military	The Ethics Committee of General Hospital of Shenyang Military	2015-015
Second Department of General Surgery, General Hospital of Benxi Iron and Steel Co., Ltd.	The Ethics Committee of General Hospital of Benxi Iron and Steel Co., Ltd.	2015-011
First Department of General	The Ethics Committee of Affiliated	2015-022

For peer review only

Surgery, Affiliated Central Hospital of Shenyang Medical University	Central Hospital of Shenyang Medical University	
Department of General Surgery, First Hospital of Dandong	The Ethics Committee of First Hospital of Dandong	2016-016
Shengjing Hospital of China Medical University	The Ethics Committee of Shengjing Hospital of China Medical University	2016-010

For peer review only

Ethics Committee of the Fourth Affiliated Hospital of China Medical University
4# CHONG-SHAN EAST ROAD, SHENYANG, P.R.CHINA 110032

Informed Consent Form for Clinical Studies

[Name of Principal Investigator] Hang-yu Li
[Name of Organization] The Fourth Affiliated Hospital of China Medical University, China
[Name of Proposal and version] Anterior transversalis fascia approach versus preperitoneal space approach for inguinal hernia repair in residents in Northern China: study protocol for a prospective, multi-center, randomized, controlled trial (Version 1.0)

This Informed Consent Form has two parts:

- **Information Sheet (to share information about the research with you)**
- **Certificate of Consent (for signatures if you agree to take part)**

You will be given a copy of the full Informed Consent Form

PART I: Information Sheet

Introduction
I am Dr. Hang-yu Li, working for the Fourth Affiliated Hospital of China Medical University. We are doing research on different surgical techniques used to repair abnormal wall defects. I am going to give you information and invite you to be part of this research. You do not have to decide today whether or not you will participate in the research. Before you decide, you can talk to anyone you feel comfortable with about the research.

There may be some words that you do not understand. Please ask me to stop as we go through the information and I will take time to explain. If you have questions later, you can ask them of me, the study doctor or the staff.

Purpose of the research
There are few reports on the effects of different inguinal hernia repair approaches on postoperative complications, particularly regarding severe postoperative complications, in patients from northern China. The incidence of severe complications after inguinal hernia repair is relatively low, but this surgery can lead to physical impairment or organ dysfunction that greatly decreases patient quality of life and places a heavy burden on the patient's family and society. This study will compare the effectiveness of inguinal hernia repair via the anterior transversalis fascia approach versus the preperitoneal approach in patients from northern China in terms of postoperative complications, quality of life, and cost-effectiveness. This study aims to determine the optimal method for inguinal hernia repair that is suitable for the anatomic features of the inguinal region for local patients.

Type of Research Intervention
Briefly state the type of intervention that will be undertaken. This will be expanded upon in the procedures section but it may be helpful and less confusing to the participant if they

For peer review only - http://bmjopen.bmj.com/site/about/guidelines.xhtml

know from the very beginning whether, for example, the research involves a vaccine, an interview, a biopsy or a series of finger pricks.

You will be undergone inguinal hernia repair via the anterior transversalis fascia approach or the preperitoneal approach. Also you will be followed-up at the following time-points: at baseline (at admission), pre-discharge, and at 1 week, 1 month, 3 months (hospital visit or telephone follow-up), 1 year (telephone follow-up), and 2 years after surgery (telephone follow-up).

Participant selection

We are inviting all male adults with inguinal hernia who attend our hospital to participate in the research.

- ***Example of question to elucidate understanding:*** *Do you know why we are asking you to take part in this study? Do you know what the study is about?*

Voluntary Participation

Your participation in this research is entirely voluntary. It is your choice whether to participate or not. Whether you choose to participate or not, all the services you receive at this hospital will continue and nothing will change. If you choose not to participate in this research project, you will offered the treatment that is routinely offered in this hospital for inguinal hernia repair, and we will tell you more about it later. You may change your mind later and stop participating even if you agreed earlier.

- ***Examples of question to elucidate understanding:*** *If you decide not to take part in this research study, do you know what your options are? Do you know that you do not have to take part in this research study, if you do not wish to? Do you have any questions?*

Include the following section only if the protocol is for a clinical trial:

Information on the different approaches for inguinal hernia repair

1) What is the anterior transversalis fascia repair?

An oblique skin incision will be made parallel to the inguinal groove from the inner to the outer inguinal ring. Subcutaneous tissue will be dissected until the external oblique aponeurosis is reached. Hemostasis will be achieved by electric coagulation. A4－6 cm long skin incision will be made on the external oblique aponeurosis, starting from the pubic tubercle, to fully expose the pubic tubercle and the inner ring.

The lower part of the external oblique aponeurosis will be separated from the spermatic cord. The upper part of the external oblique aponeurosis will then be separated from the internal oblique aponeurosis and dissociated to a point 3 cm above the inguinal canal wall. The overlying spermatic cord and cremaster muscle will be lifted up and disassociated about 2 cm above the pubic tubercle. The ilioinguinal nerve, external spermatic vessels, and genital nerves concomitant with the spermatic cord will be protected while the spermatic cord is lifted.

The inguinal canal will then be dissected and the herniasac will be dissociated from the surrounding tissue. The cremaster muscle fibers will be separated longitudinally and the hernia sac will be separated from the spermatic cord. The hernia sac will be dissociated until the neck of the hernia sac is reached. All abdominal contents in the hernia sac will be returned to the abdominal cavity. In cases with a small hernia sac, the sac will also be returned to the anterior peritoneal cavity to minimize postoperative pain. In cases with a large hernia sac, the distal end will be transected and opened, and the proximal part will be ligated at a high position.

A patch designed with an arc-shaped head and a swallow tail-like end that comprises two pieces (a wide top piece and a narrow bottom piece) will be sutured and fixed with non-absorbable

For peer review only - http://bmjopen.bmj.com/site/about/guidelines.xhtml

polypropylene sutures. The inner side of the patch will be sutured to the pubic tubercle and the lateral boundary of the rectus abdominis muscle. The upper border of the patch will be sutured to the conjoined tendon and its border with the inguinal ligament and iliopubic tract. The wound will then be closed using layered sutures according to anatomic position.

2) What is the preperitoneal space repair?

The skin will be cut and subcutaneous tissue will be dissected. The abdominal external oblique aponeurosis will be dissected along the fibers. The abdominal internal oblique aponeurosis and transversus abdominis muscle will be bluntly separated to expose the transverse fascia. Dissociation of the hernia sac will be done as described above in the anterior transversalis fascia repair.

Transabdominal preperitoneal or totally extraperitoneal laparoscopic techniques were used to dissociate preperitoneal space, strip spermatic cord peritoneum, anatomize and expose the pubic pore. A polypropylene patch will be placed in the prevesical space and Bogros' space to achieve repair of the pubic pore-containing area.

3) Why we compare these two approaches?

Many surgical repair methods involving patches (of varying types and materials) are available for inguinal hernia repair. At present, there is a scarcity of clinical evidence from large-sample, randomized, controlled trials investigating the effectiveness of inguinal hernia repair via the anterior transversalis fascia approach versus the preperitoneal approach. Our outcomes will provide trial-based evidence for selection of rational therapeutic regimen in the clinic.

4) What are the side-effects of the repairs?

There are few reports on the effects of different inguinal hernia repair approaches on postoperative complications, particularly regarding severe postoperative complications, in patients in northern China. The incidence of severe complications after inguinal hernia repair is relatively low, but this surgery can lead to physical impairment or organ dysfunction that greatly decreases patient quality of life and places a heavy burden on the patient's family and society.

Procedures and Protocol

You will receive the treatment of inguinal hernia repair according to national guidelines, i.e. Guidelines for the Diagnosis and Treatment of Inguinal Hernia in Adults (2014 Edition) formulated by Chinese national society and the Adult Inguinal Hernia Treatment Guidelines (Updated Edition in 2014) developed by the European Hernia Society. This means that you will be treated either by the anterior transversalis fascia approach or the preperitoneal approach during the surgery.

In strict accordance with the Consolidated Standards of Reporting Trials (CONSORT) standards, the baseline data (preoperative data), therapeutic regimen, therapeutic outcome, and medical costs during hospitalization of patients with inguinal hernia will be recorded. Patient data will be collected using an electronic data capture system (EDC).

Description of the Process

During the research, you make seven visits to the hospital.

Visit 1 (at admission, day 0):: you will be requested to

- Sign informed consent
- Recheck inclusion/exclusion criteria
- Demographic data (sex, age, body height, body mass index) and medical insurance type
- Type of hernia (indirect or direct)
- Inguinal hernia classification as type I, II, III, or IV according to the adult inguinal hernia and femoral hernia classification (revised in 2003) developed by the Hernia and Abdominal Wall Surgery Group, Society of Surgery, Chinese Medical Association [12]

For peer review only - http://bmjopen.bmj.com/site/about/guidelines.xhtml

- ASA classification
- History of diseases and risk factors* (diabetes mellitus, cardiovascular disease, lung disease, peripheral vascular disease, dementia, hypertension, smoking history and alcohol use history) (*optional evaluation items)
- Disease onset and admission (interval from first appearance of the lump, main symptoms)
- Imaging examination (ultrasound, CT)
- Laboratory examination (routine blood testing, coagulation testing, testing of blood glucose, lipids and electrolytes, and hepatic and renal function)
- Vital signs (body temperature, pulse, respiration rate, blood pressure)
- VAS pain score
- Electrocardiography
- Concomitant treatment

<u>Visit 2 (Pre-discharge): you will be tested</u>

- Vital signs (body temperature, pulse, respiration rate, blood pressure)
- VAS pain score
- Laboratory examination (routine blood testing, coagulation testing, testing of blood glucose, lipids and electrolytes, and hepatic and renal function)
- Treatment regimen (inguinal hernia-specific treatment)

Surgical approach and patches (type and fixation method); anesthesia method; drug name (generic preferred), dosage, route, start date, length of drug use will be recorded.

- Concomitant treatment: Inguinal hernia-specific treatment, maintenance therapy, and drugs used for concomitant treatment.

<u>Visit 3 (1 week after surgery via clinic visit or telephone follow-up): you will be tested</u>

- VAS pain score
- 36-Item Short Form Health Survey (SF-36) score
- Hernia recurrence
- Presence or absence of hematoma and/or seroma
- Wound infection

<u>Visit 4 (1 month after surgery via clinic visit or telephone follow-up): you will be tested</u>

- VAS pain score
- SF-36 score
- Presence or absence of hematoma and/or seroma
- Wound infection

<u>Visit 5 (3 months after surgery via clinic visit or telephone follow-up): you will be tested</u>

- VAS pain score
- SF-36 score
- Hernia recurrence
- Presence or absence of hematoma and/or seroma
- Wound infection

For peer review only - http://bmjopen.bmj.com/site/about/guidelines.xhtml

Visit 6 (1 year after surgery via telephone follow-up): you will be tested

- VAS pain score
- SF-36 score
- Hernia recurrence
- Presence or absence of hematoma and/or seroma
- Wound infection

Visit 7 (2 years after surgery via telephone follow-up): you will be tested

- VAS pain score
- SF-36 score
- Hernia recurrence
- Presence or absence of hematoma and/or seroma
- Wound infection

Duration

In total, you will be asked to come 7 times to the hospital in 2 years. At the end of 2 years, the research will be finished.

- ***Examples of question to elucidate understanding:*** *Can you tell me if you remember the number of times that we are asking you to come to the hospital to complete the treatment? The research project? How many injections will you be given? How many tablets? How much blood will be taken from your veins, using a syringe and needle? Over how many weeks? Etc. Do you have any other questions? Do you want me to go through the procedures again?*

Side Effects

The incidence of severe complications after inguinal hernia repair is relatively low, any adverse medical events occurring after herniorrhaphy in patients or clinical subjects. This surgery could lead to physical impairment or organ dysfunction that decreases patient quality of life.
Serious adverse postoperative medical events involving one or more of the following criteria:

- Death, irrespective of the cause
- Life-threatening event
- Severe or permanent disability or organ dysfunction
- Hemorrhage
- Malformation, birth defect
- Hospitalization or extended hospital stay
- Re-admission to hospital
- Recurrence (symptomatic) of inguinal hernia

Risks

History of diabetes mellitus, cardiovascular disease, lung disease, peripheral vascular disease, dementia, hypertension, smoking history and alcohol use history could cause possible risks, including wound infection, rates of hematoma, seroma, hernia recurrence, intestinal obstruction, intestinal fistula, entocele, mesenteric thrombosis, femoral vein thrombosis, ischemic orchitis, painful ejaculation, varicocele, scrotal edema and etc.

While the possibility of this happening is very low, you should still be aware of the possibility. We will try to decrease the chances of this event occurring, but if something unexpected happens

For peer review only - http://bmjopen.bmj.com/site/about/guidelines.xhtml

- ***Examples of question to elucidate understanding:*** *Do you understand that these risks can happen whether or not you are in the research study? Etc. Do you have any other questions?*

Benefits

There may not be any benefit for you but your participation is likely to help us find the answer to the research question.

Reimbursements

We will waive the medical cost associated during your follow-up visit. You will not be given any other money or gifts to take part in this research.

- ***Examples of question to elucidate understanding:*** *Can you tell me if you have understood correctly the benefits that you will have if you take part in the study? Do you have any other questions?*

Confidentiality

We will not be sharing the identity of those participating in the research. The information that we collect from this research project will be kept confidential. Information about you that will be collected during the research will be put away and no-one but the researchers will be able to see it. Any information about you will have a number on it instead of your name. Only the researchers will know what your number is and we will lock that information up in the computer. It will not be shared with or given to anyone except your clinician.

- ***Example of question to elucidate understanding:*** *Did you understand the procedures that we will be using to make sure that any information that we as researchers collect about you will remain confidential? Do you have any questions about them?*

Sharing the Results

The results of this study will be disseminated via peer-reviewed publications and conference presentations. No data are available at the moment. Confidential information will not be shared.

Right to Refuse or Withdraw

You do not have to take part in this research if you do not wish to do so and refusing to participate will not affect your treatment at this hospital in any way. You will still have all the benefits that you would otherwise have at this hospital. You may stop participating in the research at any time that you wish without losing any of your rights as a patient here. Your treatment at this hospital will not be affected in any way.

Alternatives to Participating

If you do not wish to take part in the research, you will be provided with the anterior transversalis fascia repair at the hospital.

Who to Contact

If you have any questions you may ask them now or later, even after the study has started. If you wish to ask questions later, you may contact any of the following:

Name: Dr. Yang

Address: 4# Chong-shan East Road, Shenyang

Telephone number: (024)62255001

For peer review only - http://bmjopen.bmj.com/site/about/guidelines.xhtml

E-mail: 529687607@qq.com

This proposal has been reviewed and approved by Ethics Committee of the Fourth Affiliated Hospital of China Medical University, which is a committee whose task it is to make sure that research participants are protected from harm. If you wish to find about more about the IRB, contact [Dr. Yuan-zhe Jin, 4# Chong-shan East Road, Shenyang, 024-62043027.]).

- ***Example of question to elucidate understanding:*** *Do you know that you do not have to take part in this study if you do not wish to? You can say No if you wish to? Do you know that you can ask me questions later, if you wish to? Do you know that I have given the contact details of the person who can give you more information about the study?*

You can ask me any more questions about any part of the research study, if you wish to. Do you have any questions?

PART II: Certificate of Consent

I have read the foregoing information, or it has been read to me. I have had the opportunity to ask questions about it and any questions that I have asked have been answered to my satisfaction. I consent voluntarily to participate as a participant in this research.

Print Name of Participant__________________

Signature of Participant ___________________

Date ___________________________

Day/month/year

If illiterate

A literate witness must sign (if possible, this person should be selected by the participant and should have no connection to the research team). Participants who are illiterate should include their thumb-print as well.

I have witnessed the accurate reading of the consent form to the potential participant, and the individual has had the opportunity to ask questions. I confirm that the individual has given consent freely.

Print name of witness______________________ **OR** **Thumb print of participant**

Signature of witness ______________________

Date _________________________

Day/month/year

Statement by the researcher/person taking consent

I have accurately read out the information sheet to the potential participant, and to the best of my ability made sure that the participant understands that the inguinal hernia repair will be performed and a few follow-up visits requested to finish the study.

For peer review only - http://bmjopen.bmj.com/site/about/guidelines.xhtml

I confirm that the participant was given an opportunity to ask questions about the study, and all the questions asked by the participant have been answered correctly and to the best of my ability. I confirm that the individual has not been coerced into giving consent, and the consent has been given freely and voluntarily.

A copy of this ICF has been provided to the participant.

Print Name of Researcher/person taking the consent________________________

Signature of Researcher /person taking the consent__________________________

Date ___________________________
Day/month/year

For peer review only

For peer review only - http://bmjopen.bmj.com/site/about/guidelines.xhtml
